# Ucl fimbriae regulation and glycan receptor specificity contribute to gut colonisation by extra-intestinal pathogenic *Escherichia coli*

**DOI:** 10.1371/journal.ppat.1010582

**Published:** 2022-06-14

**Authors:** Steven J. Hancock, Alvin W. Lo, Thomas Ve, Christopher J. Day, Lendl Tan, Alejandra A. Mendez, Minh-Duy Phan, Nguyen Thi Khanh Nhu, Kate M. Peters, Amanda C. Richards, Brittany A. Fleming, Chyden Chang, Dalton H. Y. Ngu, Brian M. Forde, Thomas Haselhorst, Kelvin G. K. Goh, Scott A. Beatson, Michael P. Jennings, Matthew A. Mulvey, Bostjan Kobe, Mark A. Schembri

**Affiliations:** 1 School of Chemistry and Molecular Biosciences, The University of Queensland, Brisbane, Queensland, Australia; 2 Australian Infectious Diseases Research Centre, The University of Queensland, Brisbane, Queensland, Australia; 3 Institute for Glycomics, Griffith University Gold Coast Campus, Gold Coast, Queensland, Australia; 4 Division of Microbiology and Immunology, Department of Pathology, University of Utah School of Medicine, Salt Lake City, Utah, United States of America; 5 Institute for Molecular Bioscience, The University of Queensland, Brisbane, Queensland, Australia; Institut Pasteur, FRANCE

## Abstract

Extra-intestinal pathogenic *Escherichia coli* (ExPEC) belong to a critical priority group of antibiotic resistant pathogens. ExPEC establish gut reservoirs that seed infection of the urinary tract and bloodstream, but the mechanisms of gut colonisation remain to be properly understood. Ucl fimbriae are attachment organelles that facilitate ExPEC adherence. Here, we investigated cellular receptors for Ucl fimbriae and Ucl expression to define molecular mechanisms of Ucl-mediated ExPEC colonisation of the gut. We demonstrate differential expression of Ucl fimbriae in ExPEC sequence types associated with disseminated infection. Genome editing of strains from two common sequence types, F11 (ST127) and UTI89 (ST95), identified a single nucleotide polymorphism in the *ucl* promoter that changes fimbriae expression via activation by the global stress-response regulator OxyR, leading to altered gut colonisation. Structure-function analysis of the Ucl fimbriae tip-adhesin (UclD) identified high-affinity glycan receptor targets, with highest affinity for sialyllacto-N-fucopentose VI, a structure likely to be expressed on the gut epithelium. Comparison of the UclD adhesin to the homologous UcaD tip-adhesin from *Proteus mirabilis* revealed that although they possess a similar tertiary structure, apart from lacto-N-fucopentose VI that bound to both adhesins at low-micromolar affinity, they recognize different fucose- and glucose-containing oligosaccharides. Competitive surface plasmon resonance analysis together with co-structural investigation of UcaD in complex with monosaccharides revealed a broad-specificity glycan binding pocket shared between UcaD and UclD that could accommodate these interactions. Overall, our study describes a mechanism of adaptation that augments establishment of an ExPEC gut reservoir to seed disseminated infections, providing a pathway for the development of targeted anti-adhesion therapeutics.

## Author summary

ExPEC infection of the urinary tract and bloodstream is frequently seeded from an intestinal reservoir, necessitating an understanding of the mechanisms that promote gut colonisation. Here we employed molecular and structural approaches to define the regulation and function of ExPEC Ucl fimbriae as a gut colonisation factor. We describe how mutations in the non-coding regulatory region of the *ucl* promoter cause increased Ucl fimbriae expression and promote enhanced gut colonisation via tuned induction by a global regulator that senses oxygen stress. We further define the glycan receptor targets of Ucl fimbriae and characterise the structural features of the Ucl adhesin that facilitate these interactions. These findings explain how ExPEC can adapt to survival in the gut to seed extra-intestinal infection.

## Introduction

Extra-intestinal pathogenic *Escherichia coli* (ExPEC) are a major cause of multiple severe human infections, including infections of the urinary tract, prostate, bloodstream and meninges [1]. ExPEC is also a lead driver of current, new and emerging antibiotic resistance [2,3], underscored by its top-three ranking in the WHO list of 12 antibiotic resistant priority pathogens. The primary pathway of human ExPEC infection involves initial colonisation of the gut, leading to the formation of a reservoir that can seed dissemination to extra-intestinal sites. An enhanced capacity to colonise the gut may contribute to the success of globally disseminated ExPEC clones that can be differentiated based on their multi-locus sequence type (ST), the most common being ST131 [3,4].

Adherence to host tissue epithelial surfaces represents a critical step in ExPEC pathogenesis and disease development. ExPEC adherence is primarily mediated by fimbriae, long hair-like polymeric surface structures that facilitate colonisation by specific interaction with target receptors. The assembly of ExPEC fimbriae engages a conserved chaperone-usher (CU) pathway [5]. CU fimbriae biogenesis involves a dedicated periplasmic chaperone and outer membrane-bound usher, which serve as an assembly apparatus for the growing organelle. Individual fimbriae are typically comprised of ~1000 major subunit proteins polymerized into a right-handed helical structure, and a receptor-binding adhesin located at the distal tip of the organelle [6]. Tip adhesins are two-domain proteins; they possess a C-terminal pilin domain that connects the adhesin to the main fimbrial shaft, and an N-terminal lectin domain that mediates attachment to specific ligands, therefore dictating host range and tissue tropism [5].

Most ExPEC encode the capacity to produce multiple different types of CU fimbriae, the genes for which often lie within large genomic islands (GIs). For example, the reference strain UTI89 contains nine different CU fimbriae loci [7]. Type 1 and P fimbriae are the best-characterized CU fimbriae and contribute to colonisation of the bladder and kidney, respectively [8]. Ucl fimbriae (also previously annotated as F17-like or Uca-like fimbriae) are another type of CU fimbriae that contribute to ExPEC infection [9]. The *ucl* fimbrial operon consists of four genes (*uclABCD*), which encode a major subunit protein (UclA), chaperone (UclB), usher (UclC) and adhesin (UclD), respectively. Ucl fimbriae promote strong biofilm formation and attachment to exfoliated human uroepithelial cells [10,11]. Mutation of the *ucl* fimbrial genes in the UPEC strain UTI89 impacts intestinal colonization via interaction of the UclD adhesin with undefined *O*-linked oligosaccharides on colonic epithelial cells [12].

Ucl fimbriae are phylogenetically related to Uca fimbriae from *Proteus mirabilis* and F17/G fimbriae from enterotoxigenic *E*. *coli* (ETEC) [10]. *P*. *mirabilis* Uca fimbriae mediate adherence to desquamated uroepithelial cells and facilitate colonisation of the bladder and kidney in experimental mice [13,14]. F17/G fimbriae of ETEC, on the other hand, promote binding to *N*-acetyl-β-D-glucosamine (GlcNAc)-containing receptors on the microvilli of the intestinal epithelium of ruminants [15]. Although the binding of ExPEC Ucl fimbriae and *P*. *mirabilis* Uca fimbriae to a range of epithelial cells has been demonstrated, the precise receptor targets of these two closely related fimbriae remain unknown. In this study, we observed variation in the expression of Ucl fimbriae between different ExPEC strains, and identified a single nucleotide polymorphism in the *ucl* promoter region that affects Ucl fimbrial expression via differential activation by the global stress response regulator OxyR, which in turn alters intestinal colonisation. To define the receptor specificity of Ucl fimbriae, we determined the crystal structure of the UclD lectin binding domain (UclD^LD^) and the lectin binding domain of the related UcaD adhesin (UcaD^LD^). We show that both UclD^LD^ and UcaD^LD^ adopt a conserved tertiary structure, but bind to different glycans. Through crystallographic studies of UcaD^LD^ in complex with monosaccharides, as well as competitive surface plasmon resonance (SPR) analysis and molecular docking, we identified a broad-specificity binding pocket that can serve as an anchor point for receptor binding by interacting with fucose and glucose residues, which are common moieties in the identified glycan receptors. Together, these findings explain how ExPEC can adapt to survival in the gut and establish a persistent reservoir with the potential to seed extra-intestinal infection.

## Results

### Differential expression of the UclA major fimbrial subunit protein

**Fig 1:**
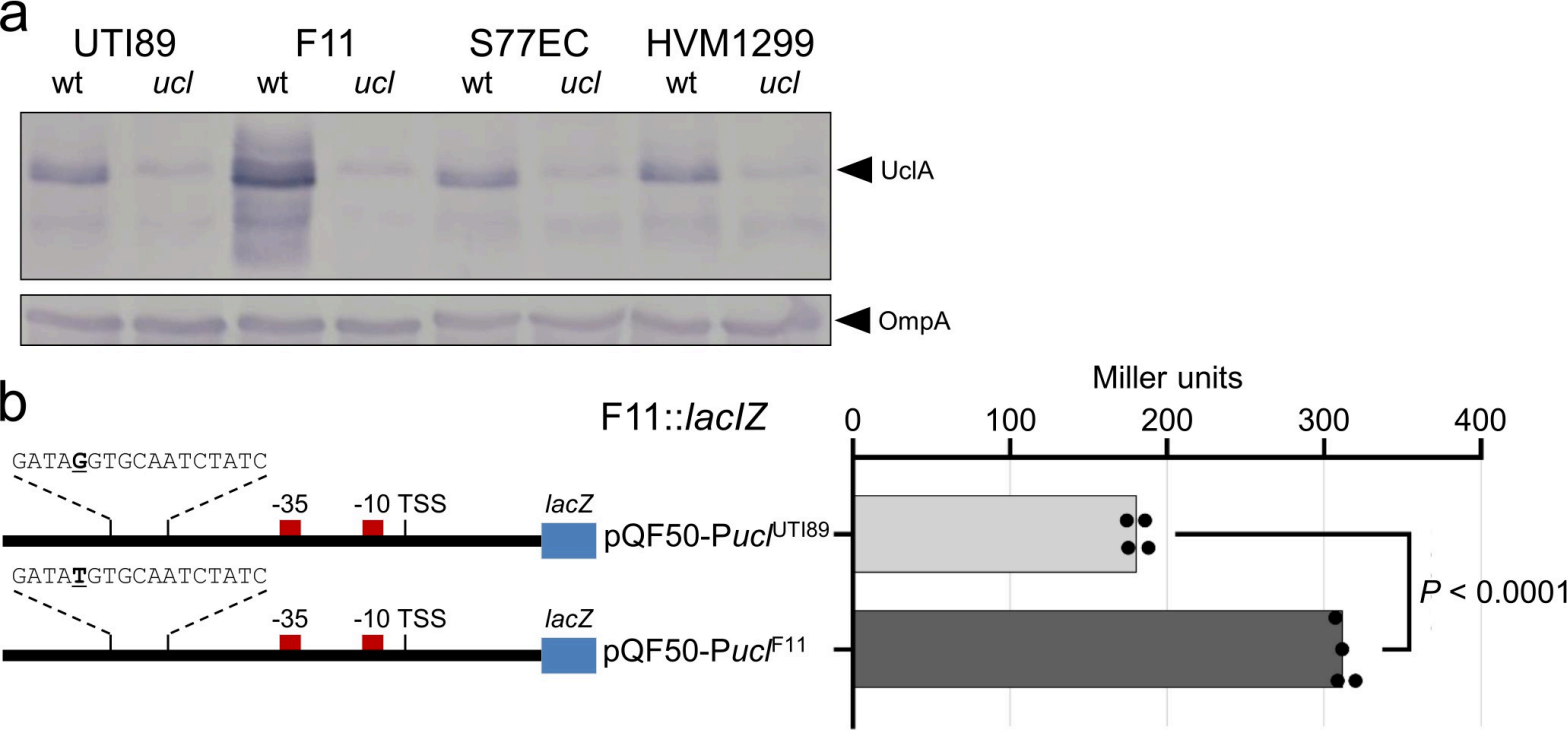
Expression of UclA differs among ExPEC strains. a) Whole-cell lysate western blot analysis of UTI89, F11, S77EC and HVM1299 and their respective *ucl* deletion mutants, using a polyclonal antibody generated against UclA^F11^ (18.9 kDa) and OmpA (loading control). b) Left, schematic diagram of the *ucl* promoter region from F11 and UTI89 cloned in the reporter plasmid pQF50. Indicated are the TSS, -10 and -35 promoter elements, and OxyR binding site. Right, β-galactosidase activity (measured in Miller units) for the pQF50-P*ucl*^F11^*-lacZ* versus pQF50-P*ucl*^UTI89^*-lacZ* fusion constructs in F11*lacZ* (p < 0.0001; one-way ANOVA with Sidak’s multiple comparisons test).

We began by comparing the expression of UclA in the ExPEC reference strains F11 (ST127), UTI89 (ST95), and two strains from the globally disseminated multidrug resistant ST131 lineage (S77EC and HVM1299) [16, 17]. These strains are part of a restricted set of ExPEC STs that we showed possess the Ucl fimbrial genes (Fig A in S1 Text), and all possess the most dominant *uclA*-10 allelic variant, one of 15 *uclA* allelic variants that we identified in our dataset of 8,247 *E*. *coli* genomes (Fig B in S1 Text). Western blot analysis using a UclA-specific antibody revealed that all strains express UclA, and that UclA expression in F11 is significantly increased compared to the other three strains (Fig 1A). In both F11 and UTI89, the two ExPEC strains used for the remainder of this study, the *uclABCD* genes reside in a conserved GI located at the *leuX* tRNA (GI-*leuX*; Fig C in S1 Text), determined based on comparative analysis of the complete F11 genome (comprising a 5,048,308 base-pair chromosome and a single 114,223 base-pair F plasmid; S1 Data) and the UTI89 genome [7].

### A single nucleotide change in the F11 *ucl* promoter region leads to increased transcription and expression of Ucl fimbriae

To understand the difference in UclA expression between F11 and UTI89, we first mapped the *ucl* transcriptional start site in F11 using 5′ RACE (indicated together with promoter elements in Fig D in S1 Text; source data in Fig E in S1 Text). We then aligned the *ucl* promoter region from F11, UTI89, S77EC and HVM1299. The *ucl* promoter region is conserved, with the exception of one change at a position 78 nucleotides upstream of the *uclA* transcription start site in F11 that differs from the other three strains (F11 T>G other strains). To examine if this single nucleotide change in F11 accounted for increased expression of UclA, the *ucl* promoter regions from F11 and UTI89 were PCR-amplified and cloned into the *lacZ* reporter plasmid pQF50, to generate plasmids pQF50-P*ucl*^F11^*-lacZ* and pQF50-P*ucl*^UTI89^*-lacZ*, respectively. The resultant plasmids were then used to transform F11 and UTI89 *lac*^*-*^ derivative strains (F11*lacI-Z* and UTI89*lacI-Z*, respectively), and β-galactosidase activity was measured following growth in Luria-Bertani (LB) broth to assess *ucl* promoter activity. Consistent with the western-blot analysis, β-galactosidase activity produced from plasmid pQF50-P*ucl*^F11^*-lacZ* was significantly higher than the corresponding activity from plasmid pQF50-P*ucl*^UTI89^*-lacZ* in both F11*lacI-Z* (Fig 1B) and UTI89*lacI-Z* (Fig D in S1 Text).

To further analyse the functional role of the F11 T>G UTI89 nucleotide in the *ucl* promoter region, we swapped the nucleotide on the chromosome of both strains using a recently described genome editing method [18]. Using this approach, we successfully exchanged the ‘T^(-78)^’ nucleotide in the *ucl* promoter of F11 (to ‘G’) and the ‘G^(-78)^’ nucleotide in the *ucl* promoter of UTI89 (to ‘T’), to generate the strains F11-P*ucl*^T-78G^ and UTI89-P*ucl*^G-78T^, respectively. Whole-genome Illumina sequencing confirmed that the F11-P*ucl*^T-78G^ and UTI89-P*ucl*^G-78T^ strains only contained the introduced single-nucleotide change. UclA-specific western-blot analysis of whole-cell lysates prepared from both sets of isogenic strains revealed that UclA expression was decreased in F11^T-78G^ compared to wild-type F11. Conversely, UclA expression was increased in UTI89^G-78T^ compared to wild-type UTI89 (Fig F in S1 Text). We also examined surface expression of Ucl fimbriae by whole-cell ELISA with our UclA-specific antibody, which reproduced the Ucl fimbriae expression profile in wild-type F11 versus F11-P*ucl*^T-78G^ and wild-type UTI89 versus UTI89-P*ucl*^G-78T^ strains (Fig G in S1 Text). Finally, transmission electron microscopy (TEM) in combination with immunogold labelling employing our UclA-specific antibody was used to directly examine the level of Ucl fimbriae produced by wild-type F11, F11-P*ucl*^T-78G^ and F11**Δ***uclA*. Gold-particle labelling was assessed from 100 randomly selected cells; 79% of wild-type F11 cells produced Ucl fimbriae compared to 12% of F11-P*ucl*^T-78G^ cells, while no labelling was observed for F11Δ*uclA* (Fig G in S1 Text). Taken together, our results demonstrate the single-nucleotide change to a ‘T’ at a position 78 nucleotides upstream of the *uclA* transcription start site in F11 is responsible for the enhanced expression of Ucl fimbriae in F11 compared to UTI89.

### OxyR regulates Ucl fimbriae expression

**Fig 2:**
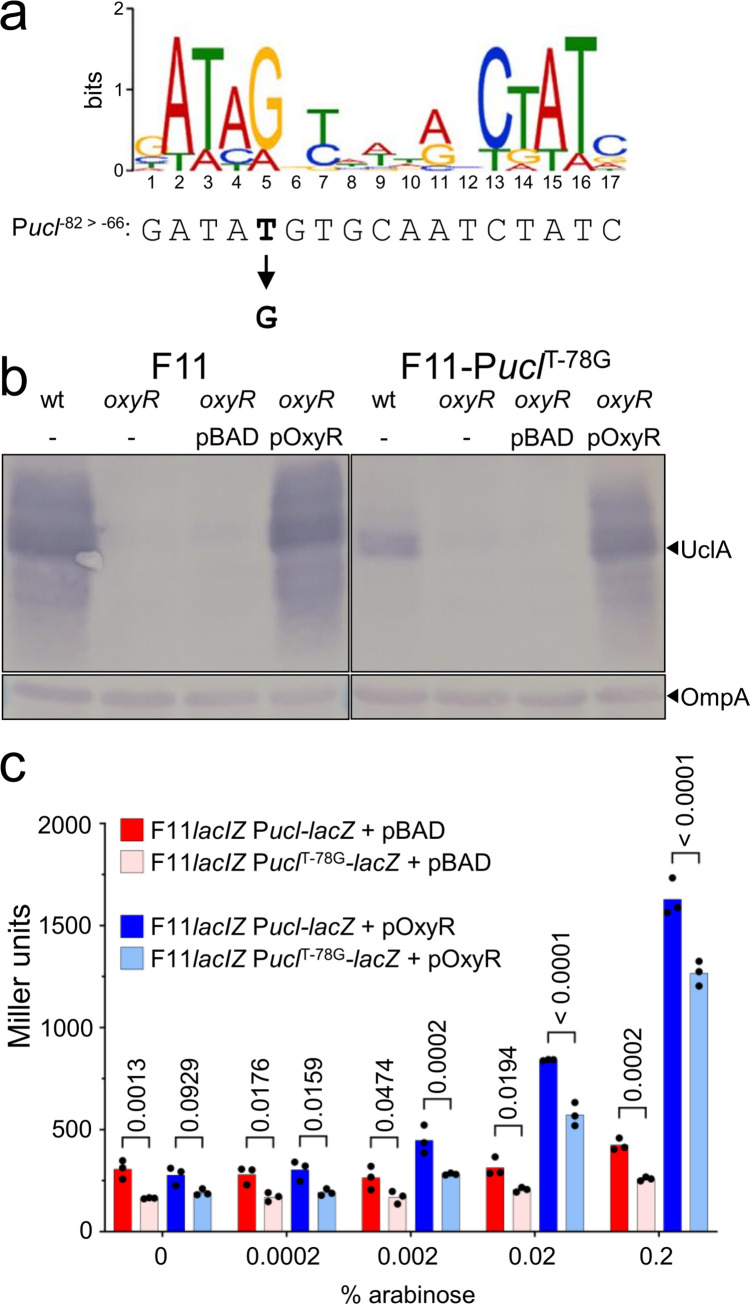
OxyR activates Ucl expression. a) Alignment of OxyR consensus binding motif [20] with the F11 P*ucl* positions -82 to -66 relative to the TSS indicated. Also indicated is the ‘T’ nucleotide at position -78, which is a ‘G’ nucleotide in UTI89 and the ST131 S77EC and HVM1299 strains. b) Whole-cell lysate western-blot analysis of F11 and F11^T-78G^ in the presence or absence of OxyR. c) Beta-galactosidase activity of F11*lacIZ* P*ucl*-*lacZ* and F11*lacIZ* P*ucl*^T-78G^-*lacZ* harbouring pBAD (vector control; red and pink shading) or pOxyR (dark blue and light blue shading). Data points show the results of triplicate biological experiments, each representative of four technical replicates, and the bar indicates the mean. P-values calculated using two-way ANOVA and Sidak’s multiple comparisons test on log_10_ transformed data.

To better understand the regulation at P*ucl*, we performed *in silico* DNA-binding protein predictions using the Virtual Footprint webserver for both the F11-P*ucl*^-78T^ and the UTI89-P*ucl*^-78G^ sequences [19]. The output showed high potential binding affinity of OxyR to both P*ucl*^-78T^ (score = 13.57) and P*ucl*^-78G^ (score = 14.38), noting this SNP occurs at position 5 of the previously defined OxyR consensus binding motif, and the P*ucl*^-78G^ sequence is a closer match to this consensus sequence [20] (Figs 1B and 2A). Based on this result, we mutated the *oxyR* gene in both F11 and F11-P*ucl*^T-78G^ and analysed UclA expression by western blotting. We observed a drastic reduction in UclA expression for both F11*oxyR* and F11-P*ucl*^T-78G^*oxyR* mutants, while complementation with the expression vector pBAD-OxyR restored UclA expression (Fig 2B). We also inserted a *lacZ* reporter as a transcriptional fusion to the P*ucl* promoter on the chromosome of F11*lacZ* and F11*lacZ*-P*ucl*^T-78G^, and transformed both strains with a plasmid containing the *oxyR* gene (pOxyR). Beta-galactosidase activity of both strains increased with increasing concentration of the inducer arabinose (Fig 2C). However, P*ucl*_F11_ was activated to a greater degree than P*ucl*^T-78G^ at all concentrations of the inducer, revealing that OxyR is required for the expression of Ucl in a dose-dependent manner and the T-78G SNP reduces OxyR mediated activation of P*ucl*.

To experimentally demonstrate that OxyR binds directly to P*ucl*^-78G^ and P*ucl*^-78T^, we expressed and purified a C-terminally 6xHis-tagged recombinant OxyR protein under non-denaturing conditions (Fig H in S1 Text). PCR-amplified Cy3-labelled DNA, representing a 261 bp region spanning the P*ucl*^-78G^/P*ucl*^-78T^ promoter (-179 to +82 relative to the TSS), was incubated with increasing concentrations of OxyR-6xHis and visualised by gel electrophoresis. Mobility of both promoter elements was retarded by the addition of OxyR, with stronger affinity observed for P*ucl*^-78G^ (first evidence of shift at 0.29 μM OxyR) compared to P*ucl*^-78T^ (first evidence of shift at 0.57 μM OxyR) (Fig H in S1 Text). The addition of excess unlabelled competitor P*ucl*^-78G^/P*ucl*^-78T^ DNA reversed binding of Cy3-labelled target DNA, demonstrating the specificity of this interaction (Fig H in S1 Text). However, binding of Cy3-labelled P*ucl*^-78G^ DNA was less effectively reversed compared to Cy3-labelled P*ucl*^-78T^ DNA, suggesting that OxyR binds to P*ucl*^-78G^ with greater affinity than P*ucl*^-78T^ in this *in vitro* binding assay. This is consistent with the P*ucl*^-78G^ sequence being a closer match to the consensus OxyR binding site, and suggests there are additional regulatory cofactors involved in the activation of Ucl fimbriae expression. Finally, to assess the broader significance of SNPs in the OxyR binding site upstream of the *uclA* gene, we compared the sequence in 698 *ucl*-positive ST127 strains downloaded from Enterobase (noting F11 belongs to ST127). In total, 217/698 (31%) of ST127 strains contained SNPs in this site, with the F11-P*ucl*^T-78G^ SNP the most common (Fig I in S1 Text). Additional examination of two *ucl*-positive strains that we previously identified from the ECOR reference collection [10, 11] revealed that one strain, ECOR-60, expressed increased UclA and possessed a P*ucl*^T-76C^ SNP (Fig J in S1 Text), demonstrating that tuning of Ucl fimbriae expression can be caused by different SNPs in the OxyR binding site.

### Increased Ucl fimbriae expression enhances colonisation of the mouse gut

**Fig 3:**
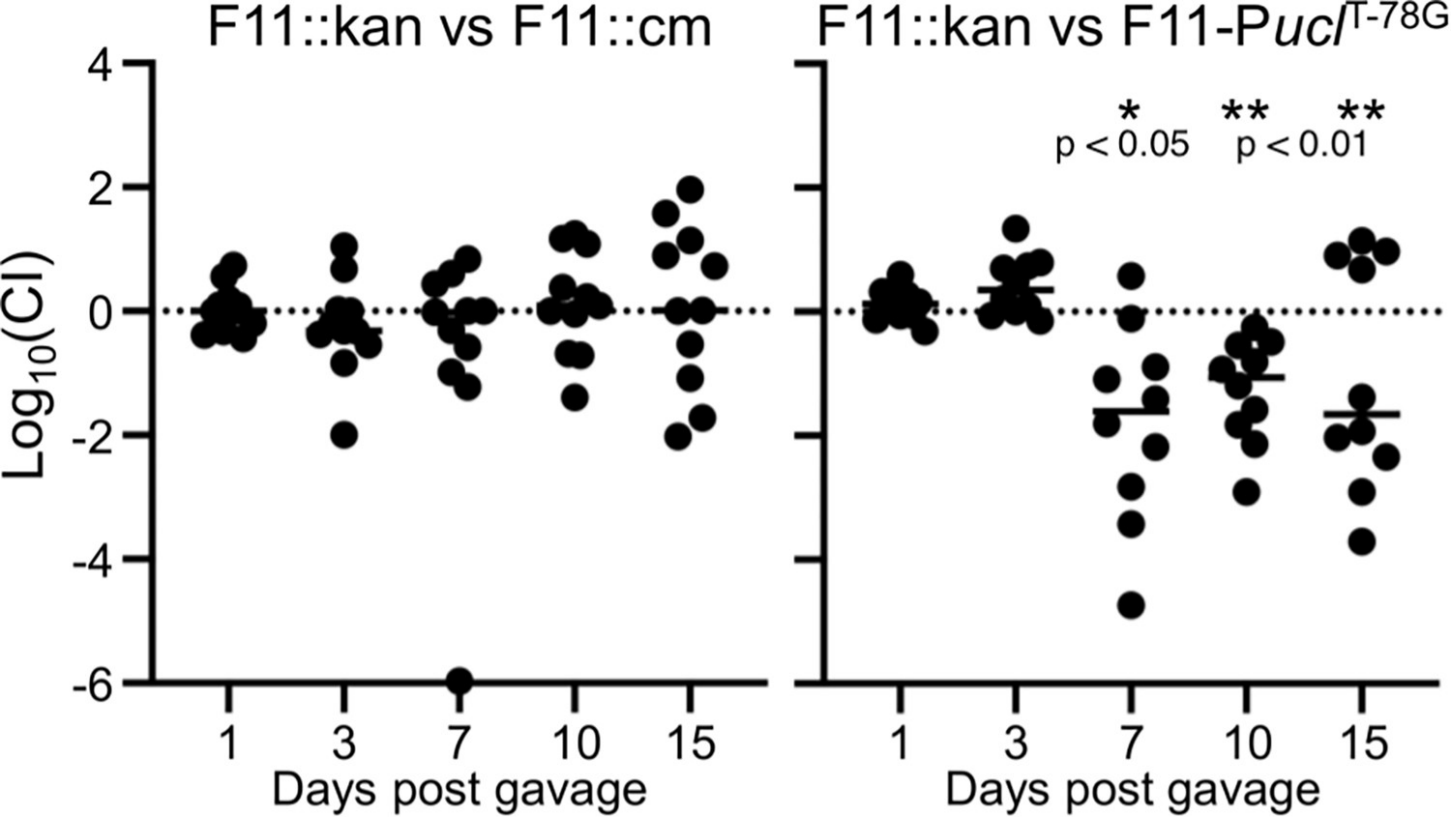
Increased Ucl fimbriae expression enhances mouse gut colonisation. Mice were inoculated with a 1:1 mixture of F11::kan vs F11::cm (control) or F11::kan vs F11-P*ucl*^T-78G^ (cm resistant). Each group contained 10–11 mice infected and monitored during two independent experiments. Differential resistance markers were used to determine bacterial loads and a competitive index was calculated for each mouse at each time point. Wilcoxon Signed Rank tests were performed on each group for each time point; * P < 0.05, ** P < 0.01.

Previous work has demonstrated that mutation of the Ucl fimbriae genes in UTI89 attenuates colonisation of the mouse gut [12]. Thus, we hypothesized that wild-type F11 would have a colonisation advantage over the F11-P*ucl*^T-78G^ mutant in similar experiments. First, we performed single infection experiments, where there was no significant difference in colonisation of the gut by wild-type F11 compared to F11-P*ucl*^T-78G^ and an F11Δ*ucl* mutant (Fig K in S1 Text). Next, we performed a more sensitive competitive experiment where mice were given a 1:1 mixture of two differentially tagged F11 strains (control) or F11 versus F11-P*ucl*^T-78G^ by oral gavage, and colonisation was monitored over time, taking advantage of kanamycin/chloramphenicol resistance markers. While there was no difference in colonisation by the differentially tagged control strains, wild-type F11 significantly outcompeted the F11-P*ucl*^T-78G^ mutant from days 7–15 post-infection (Fig 3). Thus, increased expression of Ucl fimbriae promotes enhanced colonisation of the mouse gut by F11 in a mixed infection experiment.

### Receptor specificity of the UclD tip adhesin

To extend our investigation of Ucl fimbriae beyond its regulation, we performed a detailed structure-function analysis to characterise the binding specificity of Ucl fimbriae. We initially demonstrated the tip-location of the UclD adhesin using a recombinant strain expressing Ucl fimbriae (via plasmid pUcl^F11^; MS7882) using double immuno-gold electron microscopy in combination with UclA and UclD specific antibodies (Fig L in S1 Text). Next, we determined the crystal structure of UclD^LD^ from F11 at 2.2 Å resolution (Table A in S1 Text). Given the close phylogenetic relationship between the Ucl fimbriae and the Uca fimbriae from *P*. *mirabilis* [10], in parallel we also determined the crystal structure of UcaD^LD^ (84% amino acid identity to UclD^LD^) at 1.6 Å resolution. The *ucaABCD* genes are found in ~65% of *P*. *mirabilis* genomes and are also present in other *Proteus* species (Fig M in S1 Text), and the UcaD, UclD and the related GafD adhesin from F17/G fimbriae all share significant overall amino acid identity (Fig N in S1 Text). At the time of collecting our data, there was no structural model with sufficient sequence identity available in the PDB. Thus, we used iodide SAD phasing, using a sodium iodide derivative of UclD^LD^ that diffracted to 2.85 Å resolution, to obtain phase information. The model was then used as a template for solving the structure of UcaD^LD^ by molecular replacement. The final UclD^LD^ and UcaD^LD^ models contain residues 21–215 and 21–217, respectively. Electron density was not observed for residues 43–48 (UclD^LD^) and 44–47 (UcaD^LD^), suggesting that these regions have a disordered or flexible conformation in the crystals. As expected from the high sequence identity, the two structures are similar, with r.m.s.d. of 0.6 Å for 189 equivalent Cα atoms. The UclD^LD^ and UcaD^LD^ structures are also very similar to the recently published UclD^LD^ structure from UTI89 (PDB: 5NWP) (r.m.s.d. of 0.2 Å for 189 equivalent Cα atoms), and the UcaD^LD^ structure from *P*. *mirabilis* strain HI4320 (PDB: 6H2L) (r.m.s.d. of 0.6 Å for 189 equivalent Cα atoms) [12, 21], respectively, as expected from their completely conserved protein sequences.

**Fig 4:**
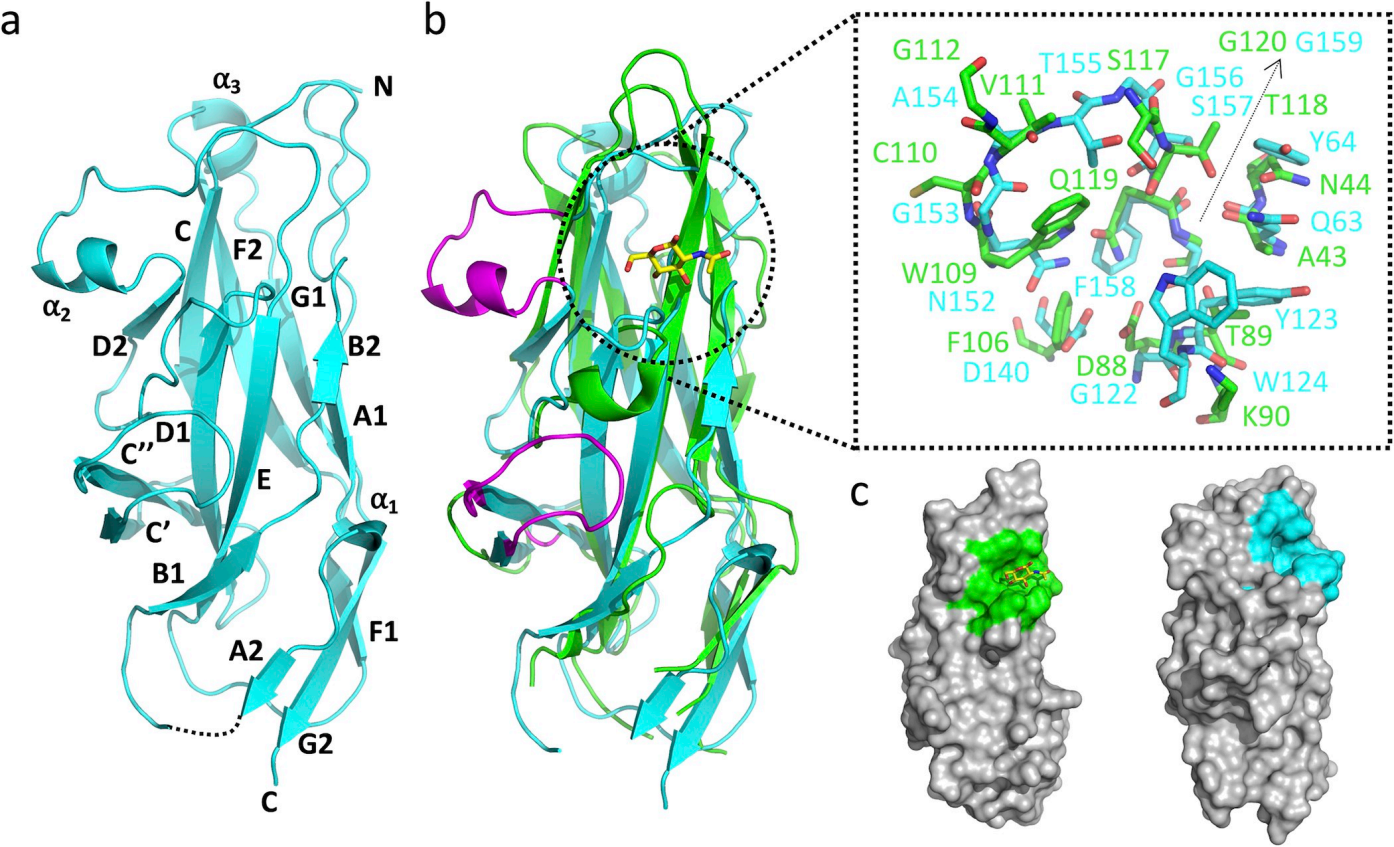
Crystal structure of UcaD^LD^. a) Cartoon representation of UcaD^LD^, with secondary-structure elements labelled A-G in accordance with previous descriptions [21]. b) Superposition of the UcaD^LD^ (cyan) and F17G (green, PDB: 1O9W) structures. The GlcNAc molecule bound to F17G is highlighted in stick representation. The insert shows the residues of the F17G GlcNAc-binding pocket, and the corresponding residues in UcaD^LD^. The two sequence insertions observed in UcaD^LD^ and UclD^LD^, compared to F17G, are highlighted in purple. c) Surface representation of F17G (left) and UcaD^LD^ (right), with the GlcNAc-binding pocket in F17G and corresponding pocket in UcaD^LD^ highlighted in green and cyan, respectively. The pockets were identified by comparing superimposed structures of F17G:GlcNAc, UcaD^LD^ and UclD^LD^. The structures were superimposed using PyMOL.

Both UclD and UcaD adopt a compact elongated Ig-like β-sandwich structure with a Greek-key topology, similar to other adhesin lectin domains (Fig 4A). A search for structures similar to UclD and UcaD in the PDB using the DALI server [22] revealed significant similarity to F17G like adhesins (Z-score of 14.3–15.8 when using UcaD^LD^ as the search model), although the sequence identity is only 14–16%. UclD^LD^ and UcaD^LD^ have a completely conserved pocket in the same region of the structure as the N-acetylglucosamine (GlcNAc)-binding site in F17G [15]. Although none of the residues in the UcaD/UclD pocket are conserved in F17G (Fig 4B and 4C) several of the residues could potentially form hydrogen bonds with a monosaccharide. The pocket also contains a tryptophan residue, which is known to mediate CH-π stacking interactions in the interaction site of other carbohydrate-binding proteins [23].

**Fig 5:**
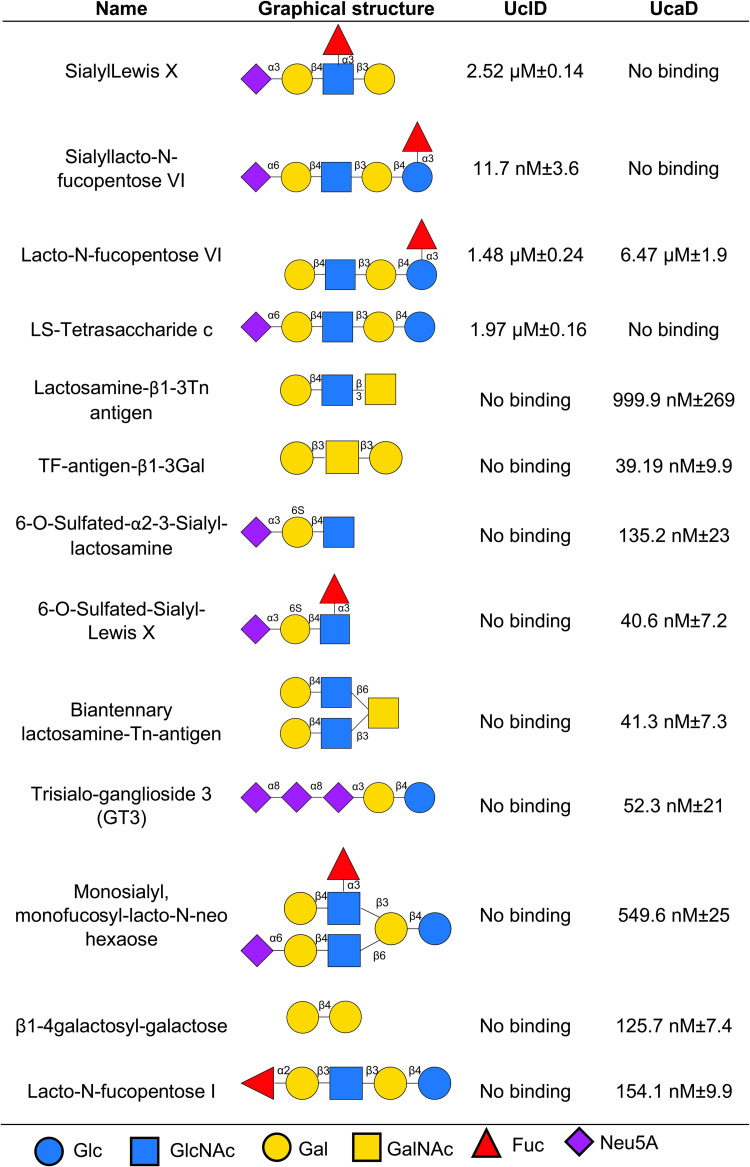
Glycan interactions and binding affinity with UclD and/or UcaD. Shown are the glycans, together with their graphical structure and binding affinity to UclD^LD^ and/or UcaD^LD^ determined by SPR.

Next, we employed a glycan array to identify potential glycans that interact with UclD^LD^ and UcaD^LD^. Of the 358 glycan structures present on the array, UclD^LD^ bound specifically to four glycans (Fig 5). Of these glycans, three were sialylated and three were α1–3 fucosylated, with two structures, sialyl-Lewis X (Neu5Acα2-3Galβ1-4(Fucα1–3)GlcNAc) and sialyllacto-N-fucopentose VI (Neu5Acα2-6Galβ1-4GlcNAcβ1-3Galβ1-4(Fucα1–3)Glc), having both sialylation and fucosylation. To further confirm and evaluate the binding affinities of UclD^LD^ with these glycans, surface plasmon resonance (SPR) analyses were undertaken (Fig 5). Overall, SPR analyses revealed the strongest binding interaction occurred between UclD^LD^ and sialyllacto-N-fucopentose VI (K_D_ = 11.72 nM ± 3.6). By contrast, UcaD^LD^ bound specifically to ten glycans from the glycan array, all but one of which, surprisingly, were different to the glycans bound by UclD^LD^ (Fig 5). SPR analysis revealed that UcaD had the highest binding affinity to Galβ1-3GalNAcβ1-3Gal-sp4, Neu5Acα2-3(6-O-Su)Galβ1-4(Fucα1–3)GlcNAcβ-sp3, Galβ1-4GlcNAcβ1-6(Galβ1-4GlcNAcβ1–3)GalNAcα-sp3 and Neu5Acα2-8Neu5Acα2-8Neu5Acα2-3Galβ1-4Glc-sp2. Both UclD and UcaD recognised lacto-N-fucopentose VI (Galβ1-4GlcNAcβ1-3Galβ1-4(Fucα1–3)Glc) at low micromolar affinities (Fig 5). Taken together, these data demonstrate that despite their high amino acid sequence identity, UclD and UcaD bind to different glycan oligosaccharides.

### Crystal structures of UcaD^LD^ bound to monosaccharides reveal an anchor point for glycan receptor binding via interaction with fucose and glucose residues

**Fig 6:**
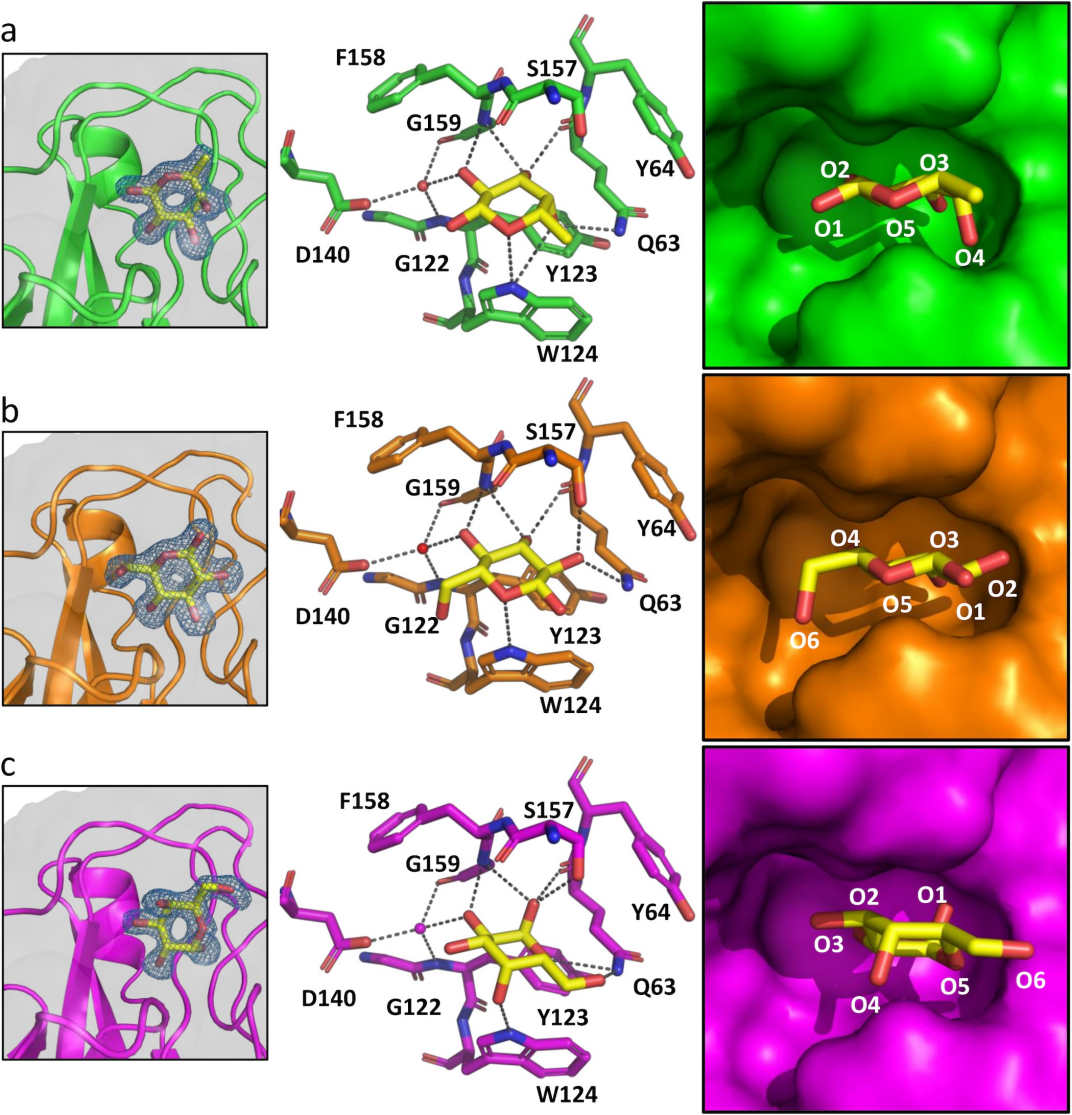
UcaD^LD^:monosaccharide complexes. Binding site details of the, a) UcaD^LD^:fucose, b) UcaD^LD^:glucose, and c) UcaD^LD^:galactose interactions. Left panel: Omit electron density maps of the ligands (m*F*_o_−D*F*_c_, blue mesh), contoured at 3.0 *σ*. Middle panel: Bound monosaccharides and the residues implicated in recognition of monosaccharides are displayed in stick representation. Interactions between the monosaccharides and residues of the binding pocket of ≤3.6 Å are shown as dashed lines. Water molecules are shown as red spheres. Right panel: Surface representation of the binding sites. In the refined structure of the UcaD^LD^:Fuc complex, positive difference density is observed adjacent to C4 (Fig P in S1 Text) suggesting that a minor fraction of the bound Fuc molecules adopt an alternate conformation in the crystal with the C4’ hydroxyl interacting with S157. This conformation was not modelled in the deposited structure.

The putative carbohydrate binding pocket in the UclD^LD^ and UcaD^LD^ structures is solvent exposed in the UcaD^LD^ crystals (Fig O [a] in S1 Text), and adopts a near-identical conformation in three different UcaD^LD^ ligand-free crystal forms (PDB: 6H1X, 6H2L [21] and 7MZO [this study]; Fig O [b] in S1 Text), suggesting that the pocket is rigid and unlikely to significantly change conformation upon ligand binding. To determine if the binding pocket corresponds to a carbohydrate receptor binding site, we soaked pre-formed UcaD^LD^ crystals with the monosaccharides fucose (Fuc), glucose (Glc), galactose (Gal), N-acetylglucosamine (GlcNAc), N-acetylgalactosamine (GalNAc), and N-acetylneuraminic acid (Neu5Ac). Clear monosaccharide electron density was observed for the UcaD^LD^ crystals soaked with either Fuc, Glc, or Gal but not with GalNAc, GlcNAc or Neu5Ac. In all three cases, the monosaccharides were observed in the same binding pocket (Fig 6 and Table B in S1 Text).

The formation of the Fuc, Glc and Gal complexes results in a similar buried surface area of the monosaccharides (77.7–82.9%; 232.6–253.2 Å^2^), and superposition of the liganded and unliganded structures reveal that neither of the three sugars affect the conformation of the protein. In all three complex structures, the carbohydrate ligand interacts directly with residues Q63, G159 and W124 of the binding pocket, but the binding mode of each ligand is different (Fig 6 and Table B in S1 Text). The Fuc molecule is bound in the β-anomer configuration (βFuc), with the C1’ hydroxyl group exposed to solvent. The Glc molecule is flipped 180° compared to Fuc and adopts the β-configuration, with the C1’ and C6’ hydroxyl groups exposed to solvent, suggesting that the binding pocket can support binding to glycans that contains a terminal Glc residue, or either a reducing end or an internal Glc residue with C1 and C6 involved in glycosidic bonds. By contrast, the Gal C1’ hydroxyl is buried; therefore, this Gal binding mode is not compatible with a glycan, because C1 cannot partake in a glycosidic bond. Furthermore, we did not observe binding of β-methyl-galactose in the UcaD^LD^ crystals, suggesting that the binding pocket cannot accommodate the β-anomer of galactose, which is the predominant configuration found in glycans.

Analysis of the UcaD residues involved in Fuc, Glc and Gal binding revealed that they are conserved in UclD. We also compared UclD, UcaD:ligand-free and UcaD:monosaccharide-bound structures, which demonstrated that the residues involved monosaccharide binding have a near identical conformation in the ligand-free structures of both UcaD and UclD (Fig O [c] in S1 Text). Thus, it is very likely that both UcaD and UclD bind in the same manner to the same set of monosaccharides.

### Competition SPR and molecular docking of lacto-N-fucopentose VI to UcaD^LD^ reveals involvement of the DE loop in receptor binding

**Fig 7:**
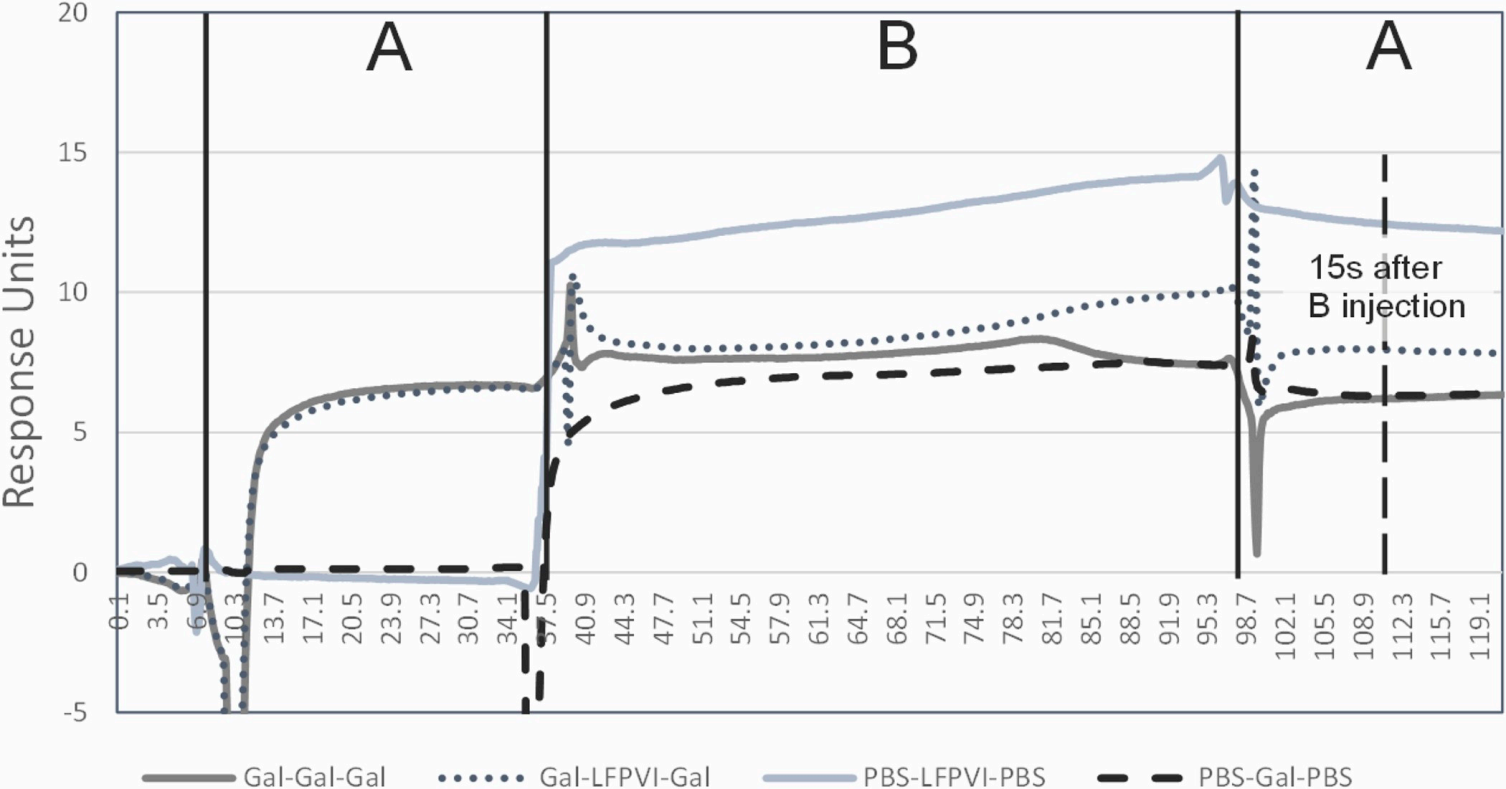
Competition between galactose and lacto-N-fucopentose VI binding to UclD. Injections are indicated as injection-A (PBS or galactose at 1 mM for 30 seconds) and injection-B (galactose at 1 mM or lacto-N-fucopentose VI at 50 μM for 60 seconds). Final responses units (RU) of injections are recorded (indicated by dashed vertical line and label), with the response of galactose equalling 6.3RU ± 0.15 at 15 seconds after injection-B and lacto-N-fucopentose VI equalling 12.4 RU ± 0.41 at 15 seconds after injection-B. The response 15 seconds after injection-B when galactose and lacto-N-fucopentose VI were competed was 7.9 RU ± 0.3. As this value sits between 6.3 and 12.4 RU, it indicates direct competition for the same site with lacto-N-fucopentose VI replacing some of the bound galactose during the 60 second injection-B.

Our original aim was to define the precise molecular interaction between the interacting glycans and UclD^LD^/UcaD^LD^; however, apart from the receptor lacto-N-fucopentose VI (Galβ1-4GlcNAcβ1-3Galβ1-4(Fucα1–3)Glc), which is recognized by both adhesins at low-micromolar affinity, we were unable to source any of the interacting glycans in sufficient amounts for further structural analysis. We were unable to observe lacto-N-fucopentose VI in crystals with either UclD^LD^ and UcaD^LD^, possibly due to steric hindrance by crystal contacts. As an alternative, we sought to determine if the monosaccharide found in the crystal structure bound to the same site on the protein as the glycans discovered using glycan array and SPR, by performing an SPR competition experiment using immobilised UcaD together with galactose and lacto-N-fucopentose VI (Fig 7). These studies revealed that pre-bound galactose (K_D_ = 162.3μM ± 12.8) could inhibit lacto-N-fucopentose VI (K_D_ = 1.48 μM ± 0.24) binding, confirming that both ligands are engaged by a common lectin site, albeit at different affinities.

**Fig 8:**
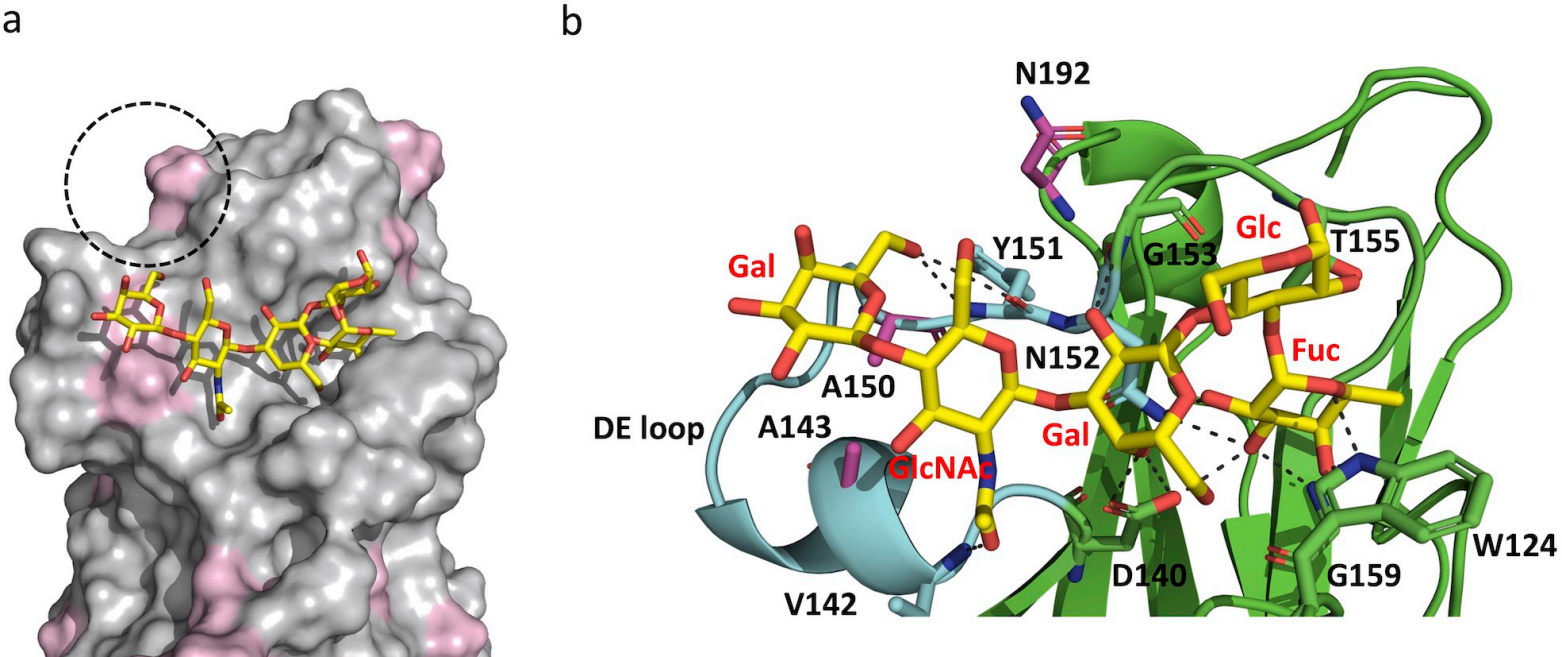
MD-derived structure of lacto-N-fucopentose VI bound to UcaD^LD^. A representative minimum-energy conformation of the glycan (yellow) shown in stick representation. a) Surface representation of UcaD^LD^. UcaD^LD^/UclD^LD^ variable residues are highlighted in purple. The dashed circle highlights a potential binding site for the sialic acid residue of sialyllacto-N-fucopentose VI, which binds to UclD^LD^ with high affinity. b) Cartoon representation of UcaD^LD^ with the DE loop highlighted in cyan. Lacto-N-fucopentose VI -interacting residues and UcaD/UclD variable residues (purple) are shown in stick representation. In this model, the glycan is stabilised by polar interactions with the side-chains of D140 (internal Gal C4’ hydroxyl) and T155 (Glc C2’ hydroxyl), and the backbone of Y151 (terminal Gal C6’ hydroxyl), V142 (GlcNAc amine group) and G153 (internal Gal C2’ hydroxyl).

Next, we combined docking with molecular dynamics (MD) simulations to obtain further insight into the structural basis of glycan-protein interactions. The lacto-N-fucopentose VI receptor was selected for this analysis, since it is recognised by both UcaD and UclD at low-micromolar affinity. We used the UcaD^LD^:βFuc structure to guide the molecular docking of lacto-N-fucopentose VI, which was further optimized by a 40 ns MD simulation. During this MD simulation, the glycan remained bound to UcaD^LD^, without dissociation for the entire duration of the simulation (Fig 8A). The αFuc moiety of the receptor remained anchored in the monosaccharide-binding pocket (Fig 8A), but underwent a ~ 90° counter clockwise rotation compared to the crystal structure (Fig Q in S1 Text). This is not surprising, as the C1’ hydroxyl group of the αFuc molecule, based on the UcaD^LD^:βFuc structure, faces the protein surface, and therefore the α-1,3 linked glucose residue of the receptor cannot be accommodated by the lectin without a change in the fucose binding mode (Fig Q in S1 Text).

The DE loop, which has a large insertion in UclD^LD^ and UcaD^LD^ relative to F17G interacts with the terminal Gal and GlcNAc residues of lacto-N-fucopentose VI in the MD derived structure. This loop contains two variable residues; A143 and A150 in UcaD^LD^ correspond to a glycine and threonine, respectively, in UclD^LD^ (Fig 8A and 8B). The differences in surface properties caused by these two amino acid substitutions could impact glycan specificity and affinity. The addition of a sialic acid residue to the lacto-N-fucopentose VI receptor via α2.6 glycosidic bond to the terminal Gal increases the binding affinity for UclD^LD^ by two orders of magnitude, while no binding is detected for UcaD^LD^ (Fig 5). Based on the docked UcaD^LD^:lacto-N-fucopentose VI complex, the sialic acid residue could reside in close proximity to another surface region with variable UclD^LD^/UcaD^LD^ regions (Fig 8A). In particular, the swap of asparagine 192 in UcaD^LD^ to a positively charged lysine residue in UclD^LD^ could be the reason for the increased binding affinity for sialyllacto-N-fucopentose VI via its negatively charged sialic acid.

In summary, our structural studies reveal a monosaccharide-binding pocket with broad specificity, recognising sugars such as Glc, Fuc and Gal, but not amino sugars such as sialic acid, GlcNAc and GalNAc, which may be due to the inability of the pocket to accommodate the more bulky N-acetyl amine group. Only fucose binding is consistent with the identified UcaD and UclD glycan receptors, but glycan anchoring to these lectins via fucose is likely to require a different binding mode compared to the UcaD:Fuc crystal structure. Our SPR competition experiments and molecular docking analysis suggests that the extended DE loop, which is a unique feature of the UcaD and UclD adhesins, plays an important role in engaging with fucose-containing glycan receptors.

## Discussion

Adherence is a pivotal initial step in bacterial colonisation and disease pathogenesis. Fimbriae of the CU secretion pathway represent one of the primary factors for mediating colonisation of specific host tissues. Here, we describe a mechanism of ExPEC adaptation to intestinal colonisation via enhanced expression of Ucl fimbriae, and complement this with a detailed description of the Ucl fimbriae glycan receptor repertoire as well as the identification of a broad-specificity glycan binding pocket in the UclD fimbrial adhesin that could accommodate these interactions.

The genes encoding Ucl fimbriae were found within a limited range of *E*. *coli* STs, and predominantly within STs from the B2 phylogenetic group. This variable prevalence extends previous studies [9, 10, 12], and complete sequencing of the F11 genome defined the precise location of the *ucl* genes within GI-*leuX*. Bioinformatic analysis also demonstrated allelic variation of the major subunit *uclA* gene, providing evidence for immune pressure driving antigenic variation, as observed for other fimbriae such as type 1 and P fimbriae [24, 25]. Comparative analysis between F11 and several other UPEC strains, including UTI89, revealed a striking difference in the level of expression of the UclA major subunit protein, and this correlated with a SNP in the regulatory region that impacts promoter activity. SNPs that alter host colonisation have mostly been described within coding regions, an example being minor pathoadaptive amino acid variations in the FimH adhesin of type 1 fimbriae that enhance UPEC colonisation of the bladder in experimental mice [26, 27]. SNPs within non-coding regulatory regions, albeit less well-characterised, can also enhance host colonisation by altering expression of key virulence determinants [28]. Here, we show the T/G^(-78)^ nucleotide in the *ucl* regulatory region impacts transcriptional activation by the global regulator OxyR, and that T^(-78)^ SNP in strain F11 drives increased colonisation of the mouse intestine. OxyR was shown to bind to both the P*ucl*^-78G^ and P*ucl*^-78T^ sequences, with higher affinity to P*ucl*^-78G^, consistent with its closer match to the consensus OxyR binding site, and suggesting the regulation of Ucl fimbriae is more complex and likely to involve other regulatory cofactors. We also show that SNPs in the OxyR binding site occur more broadly in ST127, and that at least one of these additional SNPs also causes increased UclA expression. Thus, our data provides strong evidence that mutations within this regulatory region, together with transcriptional control by OxyR and other factors yet to be identified, represent a mechanism that enables *E*. *coli* to tune Ucl fimbriae expression.

OxyR is a global transcriptional regulator that controls cellular responses to oxidative stress [29]. Our data provide the first description of OxyR regulated fimbriae expression in *E*. *coli*, an important finding given the enormous diversity of CU fimbriae. OxyR has been shown to activate the expression of fimbriae in other organisms [30, 31], and thus this mode of regulation may define a new paradigm linking fimbrial expression to oxidative stress and gut colonisation. Although oxygen availability decreases through the intestine and the large intestine is essentially anaerobic, higher levels of oxygen are found close to the gut mucosa [32]. Therefore, in line with a previous study that showed purified UcaD^LD^ binds to colonic epithelial cells in tissue sections of the mouse gut [12], it is likely that ExPEC may be exposed to oxidative stress at the gut mucosa, causing increased expression of Ucl fimbriae and thus driving enhanced colonisation. Another mechanism of OxyR activation in the gut occurs through bile, which activates stress responses that may promote interaction of *E*. *coli* with cells of the colonic epithelium [33]. Bile can also act as a mutagen, and in *Salmonella* bile exposure increases the frequency of G-C to A-T mutations, which results in enhanced expression of genes regulated by OxyR and SoxRS [34], posing an intriguing link to our observations.

The phylogenetic relatedness and role in pathogenesis of UPEC Ucl fimbriae and *P*. *mirabilis* Uca fimbriae led us to study the properties of both fimbrial adhesins. We evaluated the glycan-binding specificities of UclD^LD^ and UcaD^LD^ against glycan arrays containing 358 structures that represent host-cell surface glycoconjugates. Our analysis identified four glycans that interact with UclD and nine glycans that interact with UcaD, with all interactions confirmed and quantified by SPR. Overall, both UclD and UcaD displayed different oligosaccharide-binding specificities, with only one glycan, lacto-N-fucopentose VI, bound by both adhesins. The N-acetyllactosamine (Galβ1-4GlcNAc) motif was present in several glycans, suggesting that this oligosaccharide interacts with both UclD and UcaD. Previous studies have shown that purified Uca fimbriae bind to asialo-GM1, asialo-GM2 and lactosyl ceramide glycolipids, and that these interactions are likely to occur via the UcaA major subunit rather than the UcaD adhesin [35]. Consistent with these reports, we did not identify interactions between UclD^LD^ and these glycans.

The glycans identified for UclD are all structurally similar, featuring a sub-terminal α1–3 fucosylation and/or a terminal sialylation on a Galβ1-4GlcNAc structure. The highest affinity glycan, sialyllacto-N-fucopentose VI (Neu5Acα2-6Galβ1-4GlcNAcβ1-3Galβ1-4(Fucα1–3)Glc), contains both the fucose and sialic acid motif with a terminal α2-6Neu5Ac. Interestingly, the same structure lacking the fucose LS-tetrasaccharide c (Neu5Acα2-6Galβ1-4GlcNAcβ1-3Galβ1-4Glc) or without the sialic acid lacto-N-fucopentose VI (Galβ1-4GlcNAcβ1-3Galβ1-4(Fucα1–3)Glc) was also recognised, but both had 100-fold lower affinity compared to the complete structure (Fig 5). Sialyllacto-N-fucopentose VI was first discovered in human milk as a human milk oligosaccharide (HMO) [36]. HMOs play a key role in protecting infants from infections by mimicking host glycan cellular receptors for bacterial and viral gut pathogens, consistent with a role for sialyllacto-N-fucopentose VI as a receptor for Ucl in the human gut. Binding for UcaD showed little direct overlap with UclD, with the highest affinity binding identified for UcaD being to neutral glycans containing GalNAc on the glycan core and sulfated Galβ1-4GlcNAc structures.

Our structural data identified a monosaccharide-binding pocket in the UclD and UcaD lectin-binding domains that can act as an anchor point for interaction with glycan receptors, consistent with previous predictions [12, 21]. Although the binding pocket is localised in the same structural region as the GlcNAc-binding pocket in the F17G-like adhesins [15], the residues involved in monosaccharide binding are not conserved. Our crystal-soaking studies with various monosaccharides revealed that the pocket can accommodate multiple monosaccharide binding modes and interacts with sugars such as glucose, fucose and galactose, but not amino sugars such as sialic acid, GlcNAc, and N-acetylgalatctosesamine, which is probably due to the inability of the pocket to accommodate the more bulky N-acetyl amine group. The broad specificity is consistent with the ability of these lectins to recognise multiple glycan receptors. Our molecular docking and MD analysis suggests that the fucose residue of fucose-containing UcaD and UclD receptors can be anchored to this pocket, but has to adopt a different binding mode compared to the crystal structure in order for the additional components of these receptors to be accommodated by the lectins (Figs 8 and Q in S1 Text). Finally, our SPR competition experiments and molecular-docking analysis also suggests that the extended DE loop, which is a unique feature of the UcaD and UclD adhesins, plays an important role in engaging with fucose-containing glycan receptors.

In summary, we report a detailed molecular, structural and functional characterisation of *E*. *coli* Ucl fimbriae. We demonstrate how minor mutations in the promoter regulatory region enable tuning of Ucl fimbrial expression mediated via the global OxyR regulator, and reveal the impact of such changes on intestinal colonisation in a mouse model. Long-term persistence in the human gut despite ongoing antibiotic treatment can support the survival of ExPEC reservoirs that seed recurrent UTI [37]. Thus, blocking bacterial adherence, as shown in preclinical models for UPEC type 1 and Fml fimbriae [12, 38], represents an attractive non-antibiotic approach to address the global threat of rapidly increasing antibiotic resistance. The identification of glycan receptor targets for the UclD adhesin provides a framework to understand Ucl fimbriae tissue tropism and to develop new therapeutics that could be used as anti-adhesion drugs.

## Materials and methods

### Ethics statement

Mice were handled in accordance with protocols approved by the Institutional Animal Care and Use Committee at the University of Utah (protocol number 19–01001), according to U.S. federal guidelines indicated by the Office of Laboratory Animal Welfare and described in the Guide for the Care and Use of Laboratory Animals, 8th ed.

### Bacterial strains, mutants and culture conditions

Bacterial strains and plasmids used in this study are listed in Table C in S1 Text. Gene disruption mutants were generated using λ-Red recombinase-mediated homologous recombination as previously described [39, 40]; single-nucleotide switching of F11 and UTI to F11-P*ucl*^T-78G^ and UTI89-P*ucl*^G-78T^ was performed using pORTMAGE vectors as described previously [18, 41]. Primers used for the generation of mutants are listed in Table D in S1 Text. Bacteria were routinely grown at 37°C on solid or in liquid LB media supplemented with appropriate antibiotics ampicillin (100 μg/ml), kanamycin (25 μg/ml) or chloramphenicol (30 μg/ml).

### Bioinformatic and molecular techniques

Bioinformatic analyses were performed as described in the Supplementary Methods in S1 Text. The F11 genome was completely sequenced using a combination of long-read Pacific Biosciences Single Molecule Real-Time (SMRT) and short-read Illumina sequencing as previously described [42,43]. Methods for DNA manipulation, rapid amplification of cDNA ends (5′ RACE), mutant construction, protein preparation, immunoblotting, whole-cell ELISA and β-galactosidase assays were performed as described in the Supplementary Methods in S1 Text.

### Microscopy

Bacterial cells for electron microscopy were fixed with 2.5  % glutaraldehyde. Glow-discharged carbon-coated Formvar copper grids were placed on drops of bacterial suspension for 1 min and then washed on drops of water (3 x 1 min). Grids were negatively stained with 0.5% uranyl acetate and cells were examined under a JEOL 1010 transmission electron microscope operated at 80 kV. Micrographs were captured using an analySIS Megaview III digital camera. For immuno-gold electron microscopy, fixed bacterial cells (10 μL) were spotted onto glow-discharged carbon-coated Formvar copper grids and probed with primary antibodies (1:100 in 1% BSA/PBS) and secondary species-specific gold- labelled conjugates (5- or 10-nm size particles) and then negatively stained with 0.5% uranyl acetate.

### Electrophoretic mobility shift assays

Plasmid pOxyR was modified by PCR to introduce an in-frame sequence encoding glycine-glycine-6xhistidine. Recombinant OxyR-6xHis was purified using nickel affinity chromatography; gel shift assays were performed as previously described [44]. Briefly, purified OxyR was incubated with DNA in a 25 μl reaction containing binding buffer (20 mM Tris (pH 7.5), 50 mM KCl, 1 mM EDTA, 10% glycerol v/v) at room temperature for 15 minutes. Cy3-labelled DNA was mixed with purified protein before addition of unlabelled DNA, where appropriate. The reactions were run in 4.5% native acrylamide (Bio-Rad, catalogue no. 1610146) in 0.5× Tris-borate-EDTA (TBE) at 4°C, 90 V for one hour. Cy3-labelled DNA was visualized using an Amersham imager 800 (Cytiva, catalogue no. 29399481).

### Mouse gut colonization assays

Single and mixed competitive gut colonization assays were carried out as previously described [12, 45], with detailed methods provided in the Supplementary Methods in S1 Text. For mixed infection assays, seven- to eight-week-old female Specific Pathogen Free C3H/HeN mice (Charles River Labs) were initially treated with streptomycin, and then inoculated with 50 μl PBS containing a total of ~5 x 10^7^ CFU, comprised of a 1:1 mix of F11::kan and F11::cm (control) or F11::kan and F11-P*ucl*^T-78G^ (cm resistant). At the indicated time points post-inoculation, individual mice were briefly (3 to 10 min) placed into unused takeout boxes for weighing and feces collection. Competitive indices (CI) were calculated as the ratio of the cm resistant over kan resistant bacteria recovered from the feces divided by the ratio of the same strains within the inoculum.

### UclD^LD^ and UcaD^LD^ expression and structural analysis

The coding sequences for the *uclD and ucaD* lectin domains were amplified from *E*. *coli* strain F11 and *P*. *mirabilis* PM54 clinical isolate [46], respectively. The amplicons were cloned into pET22b, which encodes a C-terminal His-tag. Epoch Life Science made and confirmed by sequencing the pET22b::*uclD* and pET22b::*ucaD* constructs. The expression and purification of UclD^LD^ and UcaD^LD^ was performed as described in the Supplementary Methods in S1 Text. Crystals were produced by the hanging drop method. Structural analyses were carried out using DALI [22], and PyMOL (http://www.pymol.org). Figures were generated using PyMOL. The UclD^LD^ structure was refined to final R_work_/R_free_ values of 24.8%/30.6%; the UcaD^LD^ structure was refined to final R_work_/R_free_ values of 16.9%/19.2%, respectively (Table A in S1 Text). For UcaD^LD^:monosaccharide complexes, coordinates and structure factors have been deposited in the PDB with IDs 7MZQ (Fuc complex), 7MZR (Glc complex), and 7MZS (Gal complex). Detailed methods for crystallisation and structure determination of UclD^LD^, UcaD^LD^ and monosaccharide complexes, as well as methods for molecular docking and molecular dynamics simulations, are described in the Supplementary Methods in S1 Text.

### Glycan array analysis

Glycan array analysis was carried out using methods previously described [47,48], with specific details outlined in the Supplementary Methods in S1 Text. The full list of glycans analysed and the array analysis data is shown in Table E in S1 Text. Slides were scanned and analysed as outlined in the MIRAGE compliance table (Table F in S1 Text). SPR analysis was carried out using a Biacore T200 system (Cytivia) as previously described [47] with minor modifications outlined in the Supplementary Methods in S1 Text.

### Competitive surface plasmon resonance

Competition assays (ABA–injection method according to the manufacturer’s instructions; Cytiva S200) were performed between the galactose monosaccharide identified by crystallography and the best binding structure identified through glycan array and SPR analysis, lacto-N-fucopentose VI. UclD was immobilized as described above and competition analysis was performed using the ABA injection option within the kinetic/affinity methods builder. In this method, an injection molecule A at the start and end (30 seconds each injection) of the cycle and injection of a second molecule B (60 second injection) in between the two A injections. For this analysis PBS and galactose were used as ‘A’ injections, while PBS, galactose and lacto-N-fucopentose VI were used as ‘B’ injections. Galactose was used at 1 mM to ensure saturation, while lacto-N-fucopentose VI was used at 50 μM (~10 times the measured K_D_). Final responses units (RU) of injections are recorded with the response of galactose equalling ~6.3 RU 15 seconds after injection-B and lacto-N-fucopentose VI equalling ~12.4 RU 15 seconds after injection-B. If galactose and lacto-N-fucopentose VI compete for the same site then the RU 15 seconds after the injection when galactose and lacto-N-fucopentose VI is competed should be between 6.3–12.4 RU. If the response is higher than 12.4 RU then the two sugar molecules bind to different sites on the protein.

### Accession codes

The sequences for the F11 chromosome and pF11A have been deposited in the NCBI GenBank database under accession numbers CP076123 to CP076124, respectively. Raw reads for the F11 genome have been deposited to the Sequence Read Archive with accession codes SRR14581595 and SRR14581594 for Illumina and Pacific Biosciences SMRT reads, respectively. Coordinates and structure-factor files have been deposited in the Protein Data Bank (PDB), with accession codes 7MZO (UcaD), 7MZP (UclD), 7MZQ (UcaD:Fuc complex), 7MZR (UcaD:Glc complex) and 7MZS (UcaD:Gal complex).

## Supporting information

S1 Text Supplementary information comprising Supplementary Methods (DNA manipulation and genetic techniques, Bioinformatic analysis, Genetic mutagenesis of bacteria, Protein preparation, immunoblotting and whole-cell ELISA, β-galactosidase assay, 5′ RACE, Mouse gut colonization assays, Cloning, expression and purification of UclDLD and UcaDLD, Glycan array analysis, SPR Analysis, Crystallization and crystal structure determination, and Molecular docking and molecular dynamics simulations); Supplementary Figures (Figs A-Q); Supplementary Tables (Tables A-F); and Supplementary Section references. Fig A. The Ucl fimbrial operon is most frequently found in phylogroup B2 strains. Percentage of strains encoding *uclABCD* for each sequence type in the 83ST database. The bars are split to show the presence of specific *uclA* alleles; the least common *uclA* variants are summarised by the “other” designation (other: 12 variants observed; 33/404, ~8%); see Fig B in S1 Text. The *ucl* genes were most frequently found in ExPEC strains from ST12, ST73, ST127, ST131 and ST141 in the pathogenic B2 phylogroup (360/900; 40%), compared to strains from STs in phylogroups D (15/600; 2.5%) and F (3/500; 0.6%), and rarely found in strains from STs in other *E*. *coli* phylogroups. Fig B. Maximum likelihood phylogeny of *uclA* variants found in the 83ST database, with the number of each *uclA* variant indicated in the bar graph. A total of 15 *uclA* allelic variants that differed by up to 27% at the nucleotide level were identified. The most common allelic variant was *uclA*-10 (212/404; 52.5%), followed by *uclA*-5 (88/404, 21.8%) and *uclA*-13 (71/363; 17.6%); other allelic variants were infrequent. Fig C. Pair-wise sequence comparison of GI-*leuX* region of UTI89 (top), F11 (middle) and MG1655 (bottom). The integase gene adjacent to tRNA-*leuX* is coloured blue, *uclABCD* are coloured red. The grey regions connecting the genomes represent blastn output with the percent of conservation shown in the scale bar. Fig D. Analysis of the *ucl* promoter. A) Left, schematic diagram of the *ucl* promoter region from F11 and UTI89 cloned in the reporter plasmid pQF50. Indicated are the TSS, -10 and -35 promoter elements, and OxyR binding site. Right, β-galactosidase activity (measured in Miller units) for each P*ucl*-*lacZ* fusion construct in UTI89*lacZ*. Plasmid pQF50-P*ucl*^F11^*-lacZ* possessed a higher β-galactosidase activity as compared to pQF50-P*ucl*^UTI89^*-lacZ* (p < 0.0001; one-way ANOVA with Sidak’s multiple comparisons test). B) Promoter region of *ucl* operon from F11. The transcription start site is indicated as +1, with the predicted -10 and -35 core promoter elements indicated accordingly. Bolded T with an arrow indicates the single nucleotide that differed in F11, where a G is present at this position in UTI89, S77EC and HVM1299. The region from F11 and UTI89 cloned into the pQF50 *lacZ* reporter plasmid is indicated by arrows denoting the 5’ ends of primers 6571–6572. Fig E. 5’ RACE of *uclA* to map the transcription start site. Top: sequence, indicating the ATG start codon of the *uclA* gene and the +1 transcription start site. Bottom: sequence chromatogram. Fig F. a) Whole-cell lysate western-blot analysis of F11^T-78G^ and UTI89^G-78T^. Higher expression of UclA was observed in UTI89^G-78T^ mutants and lower in abundance in F11^T-78G^, compared to that of their wild-type strains. b) Qualitative analysis of 100 cells assessed for Ucl fimbriation using α-UclA immuno-gold labelling. c) Representative UclA immunogold-labelled TEM images for F11, F11Δ*uclA* and F11-P*ucl*^T-78G^. Fig G. Anti UclA whole-cell ELISA. a) Whole-cell ELISA demonstrating expression of Ucl fimbriae on wild-type F11 (wt), F11Δ*ucl* (*ucl*) and F11-P*ucl*^T-78G^ (T-78G), as well as wild-type UTI89 (wt), UTI89Δ*ucl* (*ucl*) and UTI89-P*ucl*^G-78T^. Ucl fimbriae were detected using UclA-specific polyclonal antibody. b) Control whole cell ELISA of wild-type F11 (wt), F11Δ*ucl* (*ucl*) and F11-P*ucl*^T-78G^ (T-78G), as well as wild-type UTI89 (wt), UTI89Δ*ucl* (*ucl*) and UTI89-P*ucl*^G-78T^ employing a general *E*. *coli* antibody (Life Research B65001R). The black dots show individual measurements for four technical replicates from three biological replicates (n = 12); the grey bar indicates the mean. Statistical analyses were performed by one-way ANOVA with Sidak’s multiple comparisons test. Fig H. Binding of OxyR to the *ucl* promoter region (P*ucl*). a, Coomassie-stained SDS-PAGE of OxyR-6xHis. Lane M: PageRuler Prestained Protein Ladder (Life Technologies, catalogue no. 26616), Lane 2: Nickel-affinity purified OxyR-6xHis. b, Schematic diagram of the *uclABCD* operon, indicating the transcription start site (TSS), -10 and -35 promoter region, and OxyR binding sequence containing the T^(-78)^ in F11 and the G^(-78)^ in UTI89. Also indicated are the 261 bp P*ucl* PCR fragment and the 240 bp *uclC* PCR fragment (negative control) used in the gel shift assay. c, Electrophoretic mobility shift assay of the Cy3-P*ucl*^-78T^ (top) and Cy3-P*ucl*^-78G^ (bottom) fragments with OxyR and increasing concentrations of unlabelled competitor (P*ucl*-UL) DNA. Fig I. Conservation of the P*ucl* OxyR binding site in ST127. A total of 845 genomes from ST127 strains on Enterobase were downloaded, 698 of which were positive for the Ucl fimbriae genes. The OxyR binding site was extracted from each P*ucl* sequence and aligned to generate the DNA logo shown at the top of the figure. Twenty-seven unique OxyR binding sites were identified, with nucleotide sequence changes shown below the consensus sequence. The number of times each unique sequence was identified in the dataset is indicated. The OxyR binding site consensus sequence was found most frequently (n = 486), while the F11 T-78G SNP was also common (n = 96). In total, 31% of the P*ucl* OxyR binding sites contained at least one SNP compared to the consensus sequence, with the F11 T-78G SNP most prevalent (14%). Sequences containing the G-78T and T-76C are indicated. Fig J. UclA expression is increased in ECOR60. a) OxyR binding site sequences from UTI89 (consensus), F11 and ECOR60, highlighting the C-76T nucleotide sequence change in ECOR60. b) Whole-cell lysate western-blot analysis of ECOR60, ECOR63, F11 (WT) and F11-P*ucl*^T-78G^ employing a UclA-specific antibody. Higher expression of UclA was observed in ECOR60 and F11 (WT) compared to ECOR63 and F11-P*ucl*^T-78G^. The promoter region of the *ucl* operon from ECOR63 and UTI89 is identical. Fig K. Ucl fimbriae do not impact colonisation of the mouse gut in single infection experiments. Mice were inoculated wild-type F11, F11-P*ucl*^T-78G^ and an F11Δ*ucl* mutant. Each group contained 10–11 mice infected and monitored during two independent experiments. Bacterial loads were assessed over a 2-week period. Fig L. Architecture of Ucl fimbriae demonstrated by co-immunogold labelled electron microscopy. a) Electron micrograph demonstrating immunogold labelled UclA major subunit (left; 5 nm gold particles) and UclD tip adhesin (right; 10 nm gold particles) of Ucl fimbriae. b) Cartoon model of Ucl fimbriae architecture, depicting the UclA major subunit repeating protein (green), UclD tip adhesin (orange), UclC usher (yellow) and UclB chaperone (red). Also labelled are the inner membrane (IM) and outer membrane (OM) of the cell. Fig M. Prevalence of the *uclABCD* genes in *Proteus* species. Genomes were assessed from the NCBI database. The analysis was performed using tblastn for UcaA, UcaB, UcaC and UcaD, with a positive result determined for blast hits with >70% identity and >80% coverage for all four proteins. Fig N. Amino acid sequence alignment of the UclD, UcaD and GafD adhesins. Identical amino acids are shaded in black; similar amino acids are shaded in grey. The consensus sequence is indicated. Fig O. A, Crystal packing analysis of UcaD^LD^ (PDB: 7MZO). The molecules are shown in cartoon representation. The asymmetric unit, which consists of one UcaD^LD^ molecule, is shown in cyan, while symmetry-related molecules are colored orange. The monosaccharide binding region of the asymmetric unit is circled and highlighted in magenta. B, Comparison of the monosaccharide binding site region in 3 different crystal forms of ligand-free UcaD^LD^. C, Comparison of the monosaccharide binding site in the UcaD^LD^:Fuc, UcaD^LD^ and UclD^LD^ crystal structures. Fig P. Electron density map of the fucose binding site in the UcaD^LD^:Fuc complex structure. Composite (2Fo—Fc, blue, contoured at 2σ) and difference (Fo—Fc, green/red, contoured at 3.0σ) electron density map of the Fuc binding site after refinement. Positive difference density adjacent to C4 suggests that a minor fraction of the Fuc molecules in the crystal adopts an alternate conformation. Fig Q. UcaD^LD^:Fuc interactions in the MD derived structure. Interactions between fucose (yellow) and residues of the binding pocket of ≤3.6 Å are shown as dashed lines. The binding model of the fucose molecule observed in the UcaD^LD^:Fuc crystal structure is highlighted in green stick representation, for comparison. Table A. Crystallographic data. Table B. Polar interactions in the UcaD^LD^: monosaccahride complexes. Table C. List of strains and plasmids used in this study. Table D. List of primers used in this study. Table E. Glycan array analysis of UclD and UcaD. Table F. Supplementary glycan microarray document based on MIRAGE guidelines (DOI: 10.1093/glycob/cww118.
(PDF)

S1 Data F11 genomic analyses and results. This file contains information about the genome features, virulence factors, putative phage and methylome motif summary of F11.
(XLSX)
