# Supplementary material for: Ucl fimbriae regulation and glycan receptor specificity contribute to gut colonisation by extra-intestinal pathogenic Escherichia coli
Source: PLoS Pathog. 2022 Jun 14;18(6):e1010582. doi: 10.1371/journal.ppat.1010582 (PMC9236248; doi:10.1371/journal.ppat.1010582)
Supplement: S1 Text — Fig A. The Ucl fimbrial operon is most frequently found in phylogroup B2 strains. Percentage of strains encoding uclABCD for each sequence type in the 83ST database. The bars are split to show the presence of specific uclA alleles; the least common uclA variants are summarised by the “other” designation (other: 12 variants observed; 33/404, ~8%); see Fig B in S1 Text. The ucl genes were most frequently found in ExPEC strains from ST12, ST73, ST127, ST131 and ST141 in the pathogenic B2 phylogroup (360/900; 40%), compared to strains from STs in phylogroups D (15/600; 2.5%) and F (3/500; 0.6%), and rarely found in strains from STs in other E. coli phylogroups. Fig B. Maximum likelihood phylogeny of uclA variants found in the 83ST database, with the number of each uclA variant indicated in the bar graph. A total of 15 uclA allelic variants that differed by up to 27% at the nucleotide level were identified. The most common allelic variant was uclA-10 (212/404; 52.5%), followed by uclA-5 (88/404, 21.8%) and uclA-13 (71/363; 17.6%); other allelic variants were infrequent. Fig C. Pair-wise sequence comparison of GI-leuX region of UTI89 (top), F11 (middle) and MG1655 (bottom). The integase gene adjacent to tRNA-leuX is coloured blue, uclABCD are coloured red. The grey regions connecting the genomes represent blastn output with the percent of conservation shown in the scale bar. Fig D. Analysis of the ucl promoter. A) Left, schematic diagram of the ucl promoter region from F11 and UTI89 cloned in the reporter plasmid pQF50. Indicated are the TSS, -10 and -35 promoter elements, and OxyR binding site. Right, β-galactosidase activity (measured in Miller units) for each Pucl-lacZ fusion construct in UTI89lacZ. Plasmid pQF50-PuclF11-lacZ possessed a higher β-galactosidase activity as compared to pQF50-PuclUTI89-lacZ (p < 0.0001; one-way ANOVA with Sidak’s multiple comparisons test). B) Promoter region of ucl operon from F11. The transcription start site is indicated as +1, with th [file ppat.1010582.s001.pdf]

Supplementary Information

Supplementary Methods

DNA manipulation and genetic techniques. Oligonucleotides were synthesized by Integrated DNA Technologies. Plasmid DNA was isolated using the QIAprep Spin Miniprep kit (Qiagen). Chromosomal DNA was purified using the Wizard Genomic DNA Purification Kit (Promega). PCR amplification was performed using KAPA HiFi polymerase (Kapa Biosystems). PCR products and other DNA fragments were purified using the QIAquick PCR Purification kit or the QIAquick Gel Extraction kit (Qiagen). Restriction endonucleases, T4 polynucleotide kinase, T4 ligase and T4 polymerase treatment were performed following the manufacturer's recommendations (New England Biolabs). Nucleic acid quantification was performed using a Nano-Drop 2000 Spectrophotometer (Thermo Scientific). DNA sequencing was performed using Big Dye Terminator Sequencing v3.1 Cycle Sequencing (Applied Biosystems) at the Australian Equine Genomic Research Centre (AEGRC), University of Queensland.

Bioinformatic analysis. An *E. coli* genome database was generated by downloading up to 100 randomly selected sequence assemblies from each of the top 83 *E. coli* sequence types in the *E. coli* collection on EnteroBase (www.enterobase.warwick.ac.uk), a large publicly available *Enterobacteriaceae* genome sequence database [1]. This resulted in a collection of 8,247 assemblies (downloaded in January 2019) that we refer to as the 83ST database. The presence of the individual *uclA-B-C-D* genes in each genome was assessed by tblastn, using the amino acid sequence as a query, and employing a cut-off at 70 % identity and 95 % coverage. The nucleotide sequence of each gene in each genome was extracted using blastdbcmd and curated manually for false-positive hits. All alignments were constructed using ClustalO v1.2.4 [2] with default settings. Maximum-likelihood trees were produced with IQTree v1.6.8 using ModelFinder with default settings and supported by a bootstrap value of 100 [3]. Trees and metadata were visualised in Evolview [4].

Genetic mutagenesis of bacteria. Gene disruption mutants were generated using λ-Red recombinase-mediated homologous recombination as described previously [5]. Mutant strains F11*ucl*, UTI89*ucl*, S77EC*ucl* and HVM1299*ucl* were generated via a previously described three-way PCR procedure [6] to amplify a chloramphenicol (*cm*) resistance cassette from pKD3 with a 500-bp

homology region to the *ucl* genes. UTI89*lacI-Z* was generated by amplifying the *gfp-cm* cassette with a 700-bp homology region using primers 4057 (5′- tcgtcttcatcctgctcttc) and 4058 (5′-gctaaatgccgaatggttg), and the chloramphenicol resistance cassette was subsequently removed using the FLP flippase-encoding pCP20 [5]. Single-nucleotide switching of F11 and UTI to F11-P*ucl*$^{T\text{-}78G}$ and UTI89-P*ucl*$^{G\text{-}78T}$ was performed using pORTMAGE vectors as described previously [7].

Protein preparation, immunoblotting and whole-cell ELISA. Strains to be assessed were grown overnight in LB broth, supplemented with the necessary antibiotics. Whole-cell lysates were prepared by centrifuging 1 ml of overnight cultures standardised to OD_{600nm} = 1.0. Cell pellets were resuspended in 40 µl water and 5 µl of 2 M HCl and boiled for 10 min before being neutralised with 5 µl of 2 M NaOH, followed by adding 50 µl of 2×SDS loading buffer (100 mM Tris-HCl, 4 % w/v SDS, 20 % v/v glycerol, 0.2 % w/v bromophenol blue, pH 6.8). Samples were boiled for 10 min, prior to electrophoresis; a volume of 10 µl was routinely analysed. SDS-PAGE and transfer of proteins to a PVDF membrane for western-blot analysis was performed as described previously [8]. Rabbit polyclonal antibody generated against UclA, UclD or UcaD was utilised as primary antibody and detected with commercially purchased alkaline phosphatase conjugated anti-rabbit antibodies (Sigma Aldrich). SIGMAFAST BCIP/NBT (Sigma-Aldrich) were used as the substrate for detection. Western blots were scanned using the Bio-Rad GS-800 calibrated imaging densitometer.

To detect Ucl surface expression, cells were suspended in 100 mM sodium carbonate (pH 9.5) and standardised to an OD_{600} of 1. 100 µl of culture was used to coat each well of a MaxiSorp 96-well ELISA plate (Thermo Fisher 44-2404-21) overnight at 4 °C. Wells were blocked with 5 % skim milk in PBS for 1 hour at room temperature. Antibody incubations were performed in 100 µl PBS with 0.05 % Tween-20 for 1 hour at room temperature. Ucl fimbriae were probed with α-UclA, while α-*E. coli* (Life Research B65001R) was used as a cell-loading control, and secondary antibody was α-rabbit IgG, AP conjugated (Sigma A3687). All wash steps were performed three times with 250 µl PBS with 0.05 % Tween-20. The reaction was developed with 100 µl of substrate pNPP (Sigma P7998) in the absence of light and optical density was measured at 420 nm after 30 minutes.

β-galactosidase assay. β-galactosidase assays were performed essentially as described previously [9]. Briefly, strains to be assessed were grown overnight in LB broth, supplemented with the necessary antibiotics. Cultures were diluted in Z buffer (60 mM Na_2HPO_4, 40 mM NaH_2PO_4, 50 mM β-mercaptoethanol, 10 mM KCl, 1 mM $MgSO_4$, pH 7) with 0.004 % SDS and 16 % chloroform added. Samples were vortexed and incubated at 28 °C to permeabilise the cells. The substrate o-nitrophenyl-β-D-galactopyranoside (ONPG) was added to initiate the reaction, which was

subsequently stopped with sodium bicarbonate. β-galactosidase activity was assessed in quadruplicate for each strain, by measuring the absorbance at 420 nm. All experiments were performed as three independent replicates. Statistical analysis of β-galactosidase levels between F11*lacI-Z* and UTI89*lacI-Z* carrying each of the pQF50 construct was performed using an unpaired, one-way ANOVA and Sidak's multiple comparison test.

Rapid amplification of cDNA ends (5′ RACE). The transcription start site of *ucl* was determined using 5′ RACE (Version 2.0; Invitrogen) [10]. Exponentially growing cells (OD_{600nm} = 0.6) were stabilized with two-volumes of RNAprotect Bacteria Reagent (Qiagen), prior to RNA extraction using the RNeasy Mini Kit (Qiagen) and treated with rDNase I (Ambion) to remove contaminating DNA. First-strand cDNA was synthesized and PCR-amplified using the following gene specific primers: 6973 (5′- ctgagcactattcatacc) and 6974 (5′- gaatggcaaagggtgtcag), following the manufacturer's specification. Amplified cDNA ends were sequenced to determine the transcription start site.

Mouse gut colonization assays. Competitive gut colonization assays were carried out as previously described, with slight modifications [11, 12]. Seven- to eight-week-old female Specific Pathogen Free C3H/HeN mice (Charles River Labs) were first inoculated via oral gavage with 100 uL of streptomycin (1000 mg/kg in water) 24 hours prior to introduction of the F11 strains. Bacteria were grown statically from frozen stocks at 37°C for 24 hours in 250 ml flasks containing 20 ml LB and then diluted 1:1000 into fresh LB and grown for another 24 hours. Prior to gavage, 6 mL of each culture was spun down at 8,000 x g for 8 minutes and pellets were washed twice and then resuspended in phosphate-buffered saline (PBS). Each mouse was inoculated with 50 µl PBS containing a total of ~5 x 10^7 CFU, comprised of a 1:1 mix of F11::kan and F11::cm (control) or F11::kan and F11-P*ucl*$^{T-78G}$ (cm resistant). At the indicated time points post-inoculation, individual mice were briefly (3 to 10 min) placed into unused takeout boxes for weighing and feces collection. Freshly deposited feces were recovered and directly added to 1 ml of 0.7 % NaCl, weighed, and set on ice. Fecal pellets were broken up by homogenization and samples were then briefly centrifuged to pellet any large insoluble debris. Supernatants were serially diluted and spread onto LB agar plates containing either chloramphenicol (20 µg/ml) or kanamycin (50 µg/ml) to select for growth of the relevant bacterial strains. Plating assays confirmed that the mice contained no endogenous bacteria that were resistant to chloramphenicol or kanamycin prior to introduction of the ExPEC strains. Mice were housed 3 to 5 per cage and were allowed to eat (irradiated Teklad Global Soy Protein-Free Extruded chow) and drink antibiotic-free water *ad libitum*. Competitive indices (CI) were calculated as the ratio of the cm

resistant over kan resistant bacteria recovered from the feces divided by the ratio of the same strains within the inoculum.

Cloning, expression and purification of UclD^{LD} and UcaD^{LD}. The structures of the lectin domains of UclD and UcaD were predicted using Phyre (http://www.sbg.bio.ic.ac.uk/phyre2/html/) [13]. The coding sequences for the *uclD and ucaD* lectin domains were amplified from *E. coli* strain F11 and *P. mirabilis* PM54 clinical isolate [14] genomic DNAs. The amplicons were cloned into pET22b, which encodes a C-terminal His-tag. Epoch Life Science made and confirmed by sequencing the pET22b::*uclD* and pET22b::*ucaD* constructs.

E. coli BL21 (DE3) pLys harbouring pET22b::*uclD* and pET22b::*ucaD* were grown at 37 °C in LB media supplemented with 100 µg/mL ampicillin. Cells were induced at OD_{600nm} of 0.5 with 1 mM IPTG at 37 °C. Periplasmic extractions by cold osmotic shock of cells incubated overnight were carried out. UclD^{LD} and UcaD^{LD} were purified using a HiTrap nickel column (GE Healthcare). Proteins were eluted in a gradient of 0-400 mM imidazole in a buffer containing 20 mM Tris-HCl (pH 7.5) and 150 mM NaCl. Fractions containing the UclD or UcaD, as judged by SDS-PAGE, were pooled and dialysed against a buffer containing 20 mM Tris-HCl (pH 7.5) and 150 mM NaCl overnight at 4 °C. Size-exclusion chromatography (HiPrep 16/60 Sephacryl S-200 HR GE Healthcare) in 20 mM Tris-HCl (pH 7.5) and 150 mM NaCl was used to further purify UclD and UcaD, as assessed by SDS-PAGE.

Glycan array analysis. Glycan array analysis was carried out using methods previously described [15, 16]. Briefly, 2 µg of UclD and UcaD, in a final volume of 500 µL of array PBS (PBS with 2 mM $MgCl_2$ and 2mM $CaCl_2$), were pre-complexed with a mouse anti-his antibody and detected with fluorescent Alexafluor555 secondary and tertiary antibodies in a molar ration of 4:2:1 for 10 mins prior to application to the array. Slides were previously pre-blocked for 15 mins in array PBS with 0.5 % BSA. Slides were rinsed in PBS and dried by centrifugation. Protein was hybridised to the array for 15 min at room temperature in the dark. After 15 min, the slide was immersed in array PBS with 0.2% BSA and washed for 2 min. The slide was then placed in a 50 mL falcon tube and washed in array PBS for 2 min, before rinsing in clean PBS then drying by centrifugation at 200 x *g* for 5 min. The array was scanned by a ProScan Array scanner, and the results analysed by ScanArray Express software program. Binding was classified as RFU (relative fluorescence units) structure reported as positive had a value above mean background (defined as mean background plus 3 standard deviations), and had a P value of < 0.005.

SPR analysis. SPR analysis was carried out using a Biacore T200 system (Cytivia) as previously described [15] with minor modifications. Briefly, UclD and UcaD were immobilized onto flow cells of a CM5 sensor chip using amine coupling at 20 µg/mL in 10 mM sodium acetate pH 4.0 and at a flow rate of 5 µL/min for 10 minutes. Glycans were chosen based on positive glycan array results for each of the proteins with each glycan run across a dilution range starting at a maximum concentration of 100 µM and minimum concentrations tested being 1.6 nM using single cycle kinetics. Results were analysed using the Biacore T200 evaluation software.

Crystallization and crystal structure determination.

UclD^{LD}: UclD^{LD} crystals were produced using the hanging drop method, with drops containing 1 µL of protein (10 mg/mL) and 1 µL of well solution (20-25% w/v PEG 3350, 0.1 M Bis-Tris propane pH 6.5, 0.2 M sodium iodide). The crystals appeared within 1-5 days. The crystals were cryoprotected in glycerol (80% well solution and 20% (v/v) glycerol) before flash-cooling in liquid nitrogen. X-ray diffraction data were collected from a single crystal at the Australian Synchrotron MX1 beamline, using a wavelength of 0.9537 Å. Data collection was performed using Blu-Ice software [17], indexed and integrated using MOSFLM and scaled with AIMLESS within the CCP4 suite [18]. Molecular replacement was initially attempted using several published fimbrial adhesin structures as templates, but a solution could not be obtained. Because the crystallisation condition contained sodium iodide, we determined the UclD^{LD} structure by SAD phasing using the CRANK2 [19] pipeline of the CCP4 suite and a dataset (2.85 Å resolution) collected at wavelength of 1.3776 Å, where the anomalous scattering properties of iodide are still significant. Iodine atoms were located by SHELXD [20], and automatic model building was performed using Buccaneer [21] and Refmac [22]. The higher resolution UclD^{LD} structure was subsequently solved by molecular replacement using PHASER [23]. The model was refined using Phenix [24], with iterative model building carried out between rounds of refinement using Coot [25]. Structure validation was performed using MolProbity [26]. The structure was refined to final R_{work}/R_{free} values of 24.8%/30.6% (Table A in S1 Text). The moderate quality of the UclD^{LD} dataset (R_{meas} of 27.9%, Table A in S1 Text) is a likely reason for the high R_{free} value. The final UclD^{LD} model contains residues 21-215. Electron density was not observed for residues 43-48, suggesting that these regions have a disordered or flexible conformation in the crystals. The coordinates and structure factors have been deposited in the PDB with ID 7MZP.

UcaD^{LD}: UcaD^{LD} crystals were produced using the hanging drop method with drops containing 1 µL of protein (8-16 mg/mL) and 1 µL of well solution (0.1 M sodium citrate buffer pH 4.5-5.5, 2-3 M NaCl). The crystals appeared within 3-5 days. The crystals were cryoprotected in Paratone-N, before

flash-cooling in liquid nitrogen. X-ray diffraction data were collected from a single crystal at the Australian Synchrotron MX2 beamline, using a wavelength of 0.9537 Å. Data collection was performed using Blu-Ice software [17], indexed and integrated using MOSFLM and scaled with AIMLESS within the CCP4 suite [18]. The structure was solved by molecular replacement using PHASER [23] and UclD^{LD} as the template. The model was refined using Phenix [24] and structure validation was performed using MolProbity [26]. The structure was refined to final R_{work}/R_{free} values of 16.9 %/19.2 %, respectively (Table A in S1 Text). The final UcaD^{LD} model contains residues 21-217. Electron density was not observed for residues 44-47, suggesting that these regions have a disordered or flexible conformation in the crystals. Coordinates and structure factors have been deposited in the PDB with ID 7MZO.

UcaD^{LD}:monosaccharide complexes: Pre-formed UcaD^{LD} and UclD^{LD} crystals were soaked with 0.1-0.2 M of the monosaccharides Fuc, Glc, Gal, GlcNAc, GalNAc or Neu5Ac for 48 hours in the crystallisation solution (0.1 M sodium citrate buffer pH 4.5-5.5, 2-3 M NaCl). The crystals were cryoprotected in Paratone-N and flash-cooled at 100 K. X-ray diffraction data were collected from single crystals on the MX2 beamline at the Australian Synchrotron, using a wavelength of 0.9537 Å. The data-sets was processed using either MOSFLM (Gal complex), or XDS (Fuc and Glc complexes) [27] and scaled using AIMLESS in the CCP4 suite [18]. The structures were solved by molecular replacement using PHASER [23] and ligand-free UcaD^{LD} as the template. The models were refined using Phenix [24], and structure validations were performed using MolProbity [26]. Coordinates and structure factors have been deposited in the PDB with IDs 7MZQ (Fuc complex), 7MZR (Glc complex), and 7MZS (Gal complex).

Molecular docking and molecular dynamics simulations. The initial structure for MD simulations was obtained by molecular docking of lacto-N-fucopentose VI with AutoDock Vina [28], as implemented in the YASARA molecular modelling package (Ver. 16.46) [29]. A grid box covering the entire monosaccharide-binding site and surroundings was used to place lacto-N-fucopentose VI. The docked structure with the best superimposition between the fucose moiety of lacto-N-fucopentose VI and the fucose molecule observed in the UcaD^{LD}:Fuc complex was then subjected to further optimization by a 40 ns MD simulation using the AMBER force-field implemented in the YASARA software suite [29]. A representative energy-minimised snapshot from the MD trajectory was used for the analyses.

Supplementary Figures

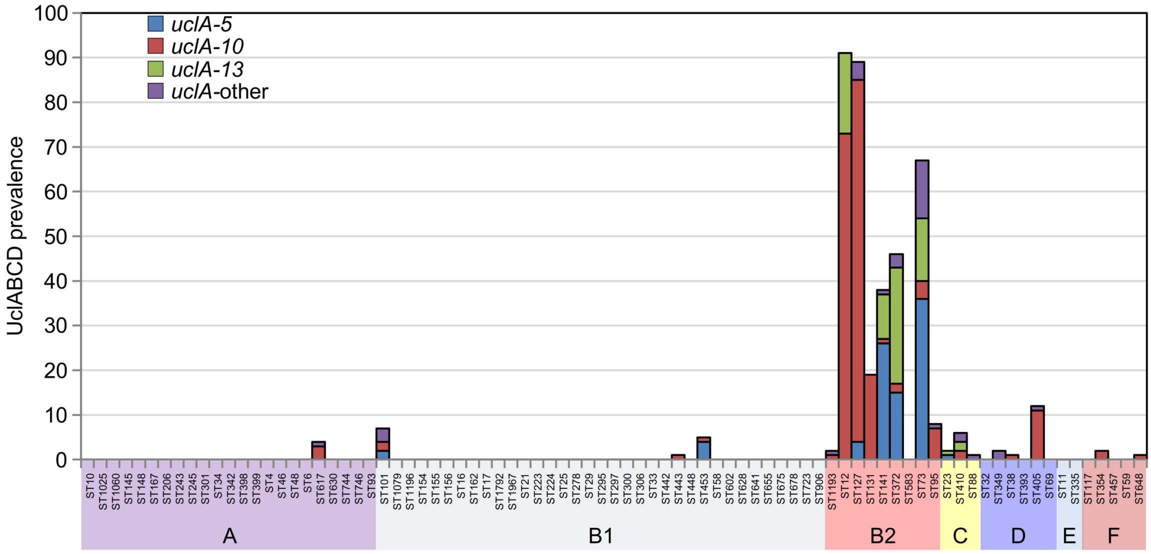

Fig A. The Ucl fimbrial operon is most frequently found in phylogroup B2 strains. Percentage of strains encoding *uclABCD* for each sequence type in the 83ST database. The bars are split to show the presence of specific *uclA* alleles; the least common *uclA* variants are summarised by the "other" designation (other: 12 variants observed; 33/404, ~8%); see Fig B in S1 Text. The *ucl* genes were most frequently found in ExPEC strains from ST12, ST73, ST127, ST131 and ST141 in the pathogenic B2 phylogroup (360/900; 40%), compared to strains from STs in phylogroups D (15/600; 2.5%) and F (3/500; 0.6%), and rarely found in strains from STs in other *E. coli* phylogroups.

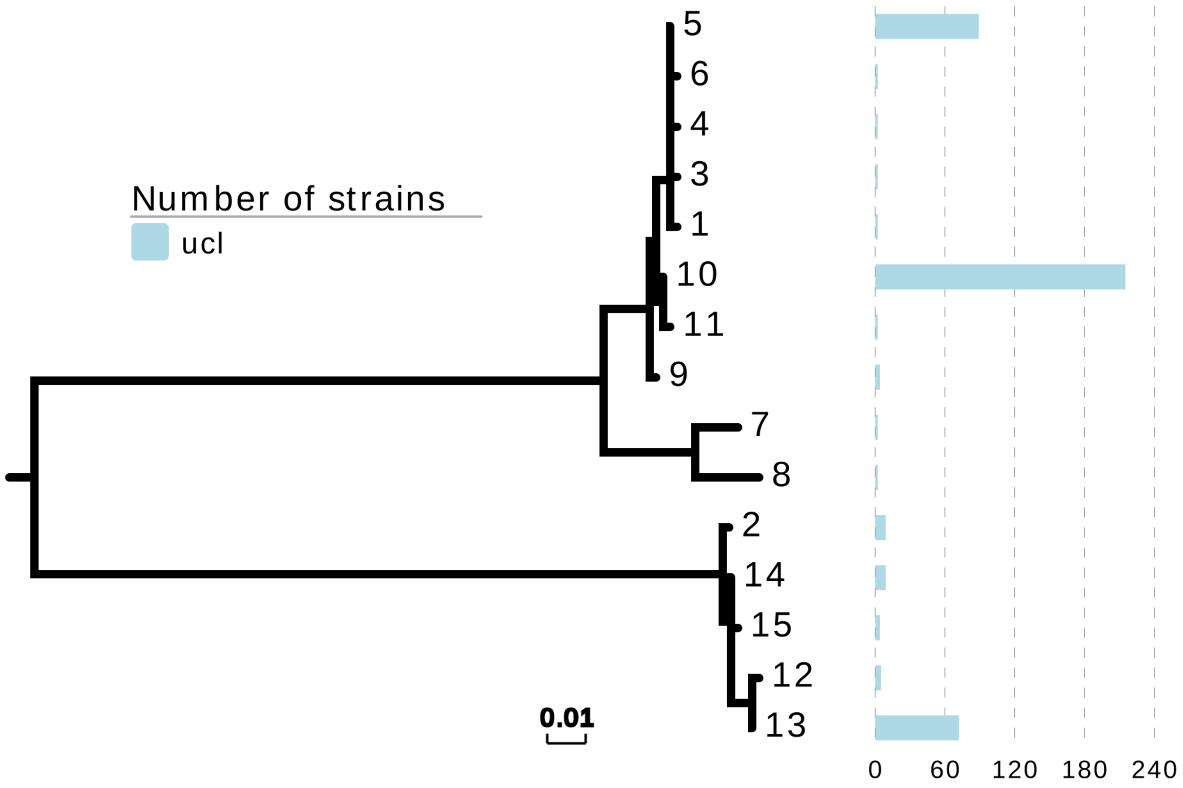

Fig B. Maximum likelihood phylogeny of *uclA* variants found in the 83ST database, with the number of each *uclA* variant indicated in the bar graph. A total of 15 *uclA* allelic variants that differed by up to 27% at the nucleotide level were identified. The most common allelic variant was *uclA*-10 (212/404; 52.5%), followed by *uclA*-5 (88/404; 21.8%) and *uclA*-13 (71/363; 17.6%); other allelic variants were infrequent.

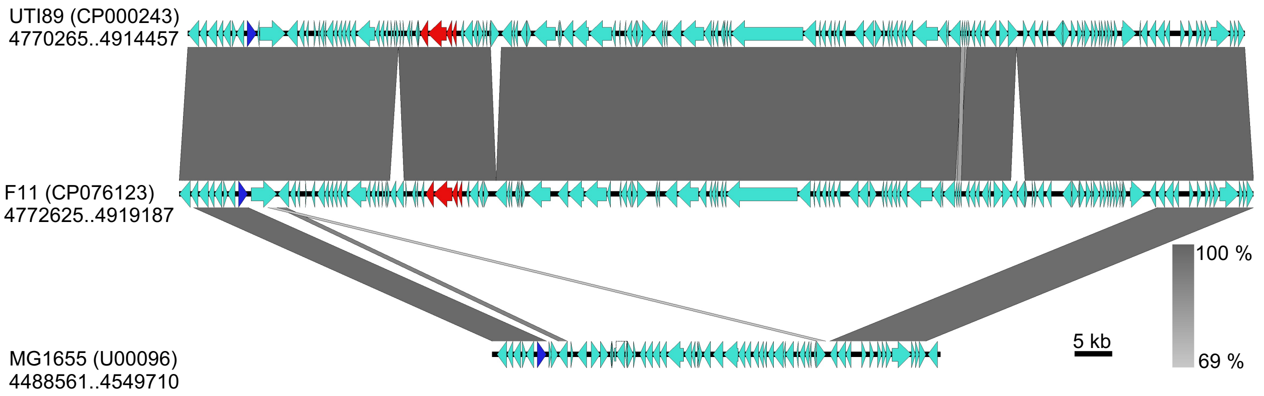

Fig C. Pair-wise sequence comparison of GI-*leuX* region of UTI89 (top), F11 (middle) and MG1655 (bottom). The integase gene adjacent to tRNA-*leuX* is coloured blue, *uclABCD* are coloured red. The grey regions connecting the genomes represent blastn output with the percent of conservation shown in the scale bar.

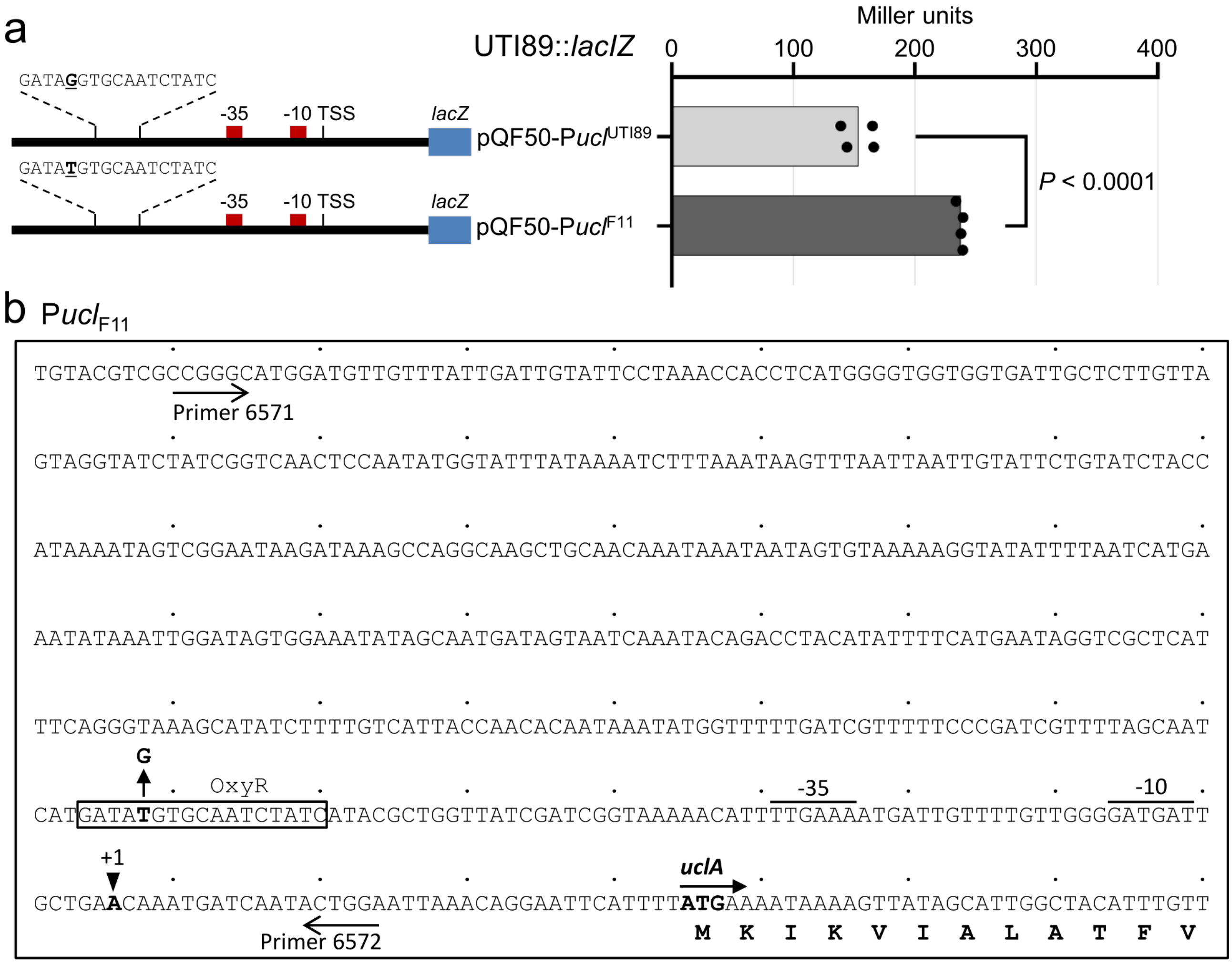

Fig D. Analysis of the *ucl* promoter. a) Left, schematic diagram of the *ucl* promoter region from F11 and UTI89 cloned in the reporter plasmid pQF50. Indicated are the TSS, -10 and -35 promoter elements, and OxyR binding site. Right, β-galactosidase activity (measured in Miller units) for each P*ucl-lacZ* fusion construct in UTI89*lacZ*. Plasmid pQF50-P*ucl*^{F11}-*lacZ* possessed a higher β-galactosidase activity as compared to pQF50-P*ucl*^{UTI89}-*lacZ* ($p < 0.0001$; one-way ANOVA with Sidak's multiple comparisons test). b) Promoter region of *ucl* operon from F11. The transcription start site is indicated as +1, with the predicted -10 and -35 core promoter elements indicated accordingly. Bolded T with an arrow indicates the single nucleotide that differed in F11, where a G is present at this position in UTI89, S77EC and HVM1299. The region from F11 and UTI89 cloned into the pQF50 *lacZ* reporter plasmid is indicated by arrows denoting the 5' ends of primers 6571-6572.

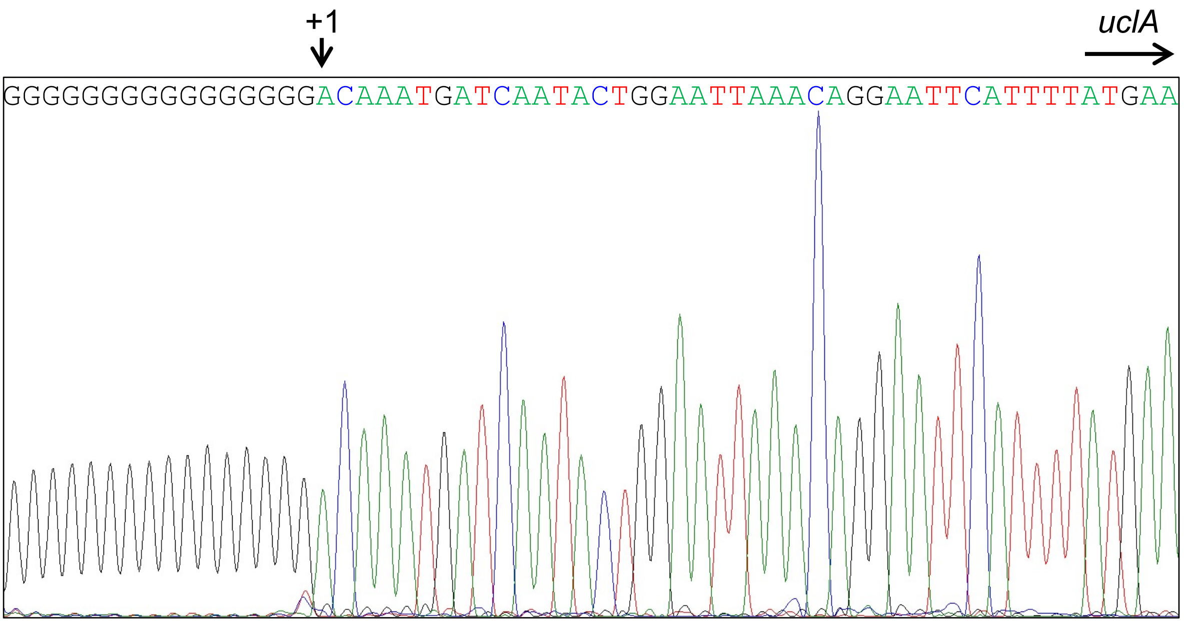

Fig E. 5’ RACE of *uclA* to map the transcription start site. Top: sequence, indicating the ATG start codon of the *uclA* gene and the +1 transcription start site. Bottom: sequence chromatogram.

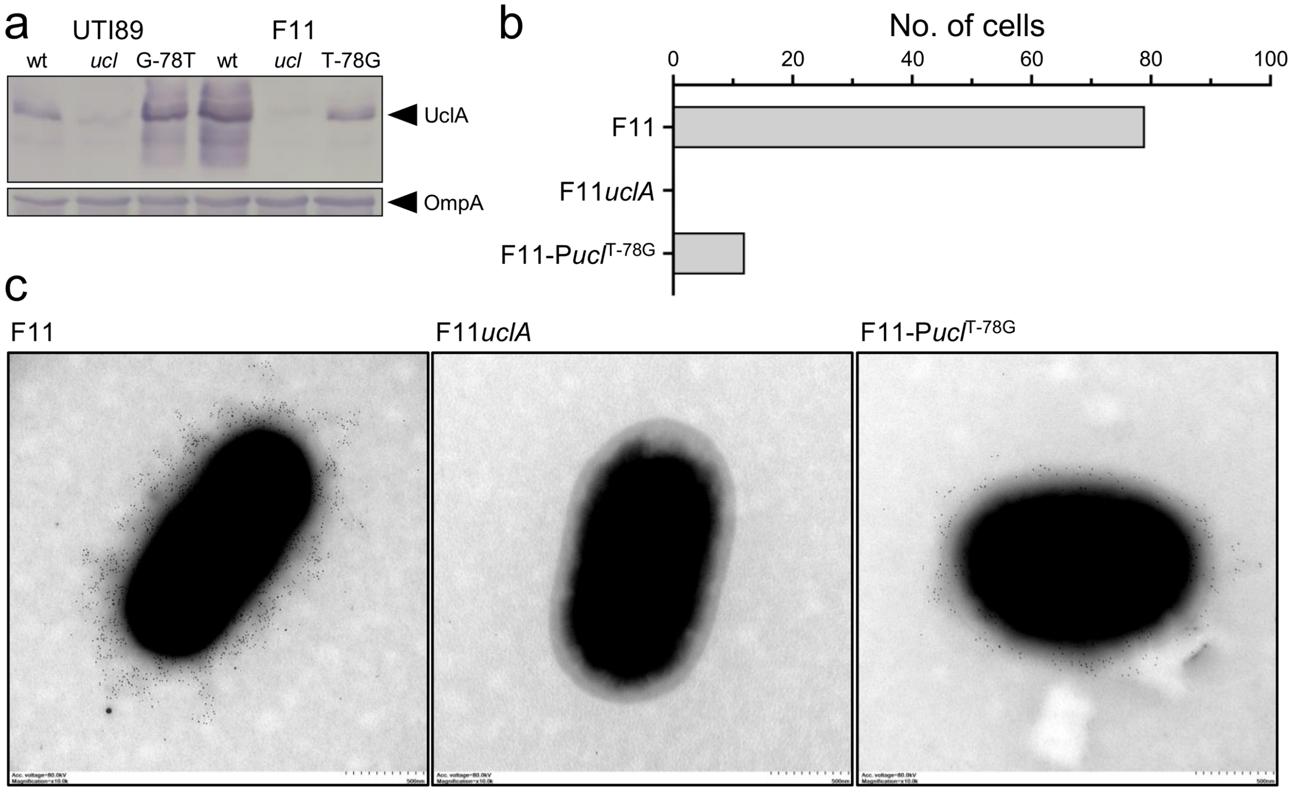

Fig F. a) Whole-cell lysate western-blot analysis of F11$^{T\text{-}78G}$ and UTI89$^{G\text{-}78T}$. Higher expression of UclA was observed in UTI89$^{G\text{-}78T}$ mutants and lower in abundance in F11$^{T\text{-}78G}$, compared to that of their wild-type strains. b) Qualitative analysis of 100 cells assessed for Ucl fimbriation using α-UclA immuno-gold labelling. c) Representative UclA immunogold-labelled TEM images for F11, F11Δ*uclA* and F11-P*ucl*$^{T\text{-}78G}$.

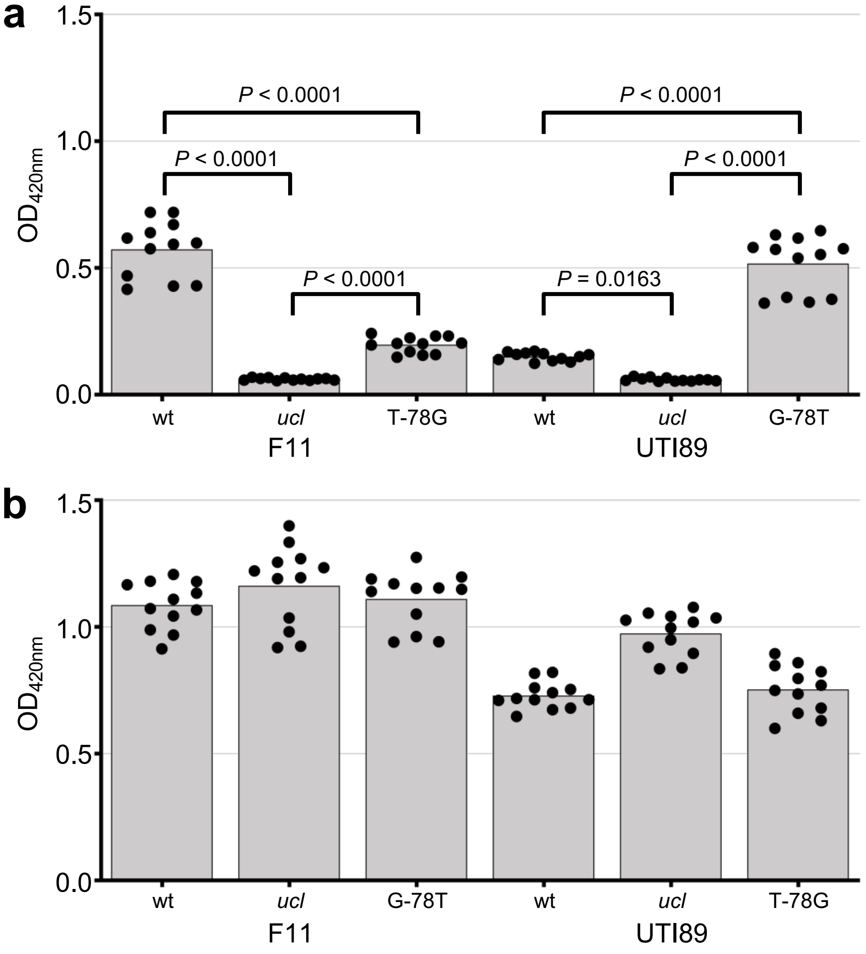

Fig G. Anti UclA whole-cell ELISA. a) Whole-cell ELISA demonstrating expression of Ucl fimbriae on wild-type F11 (wt), F11Δ*ucl* (*ucl*) and F11-P*ucl*$^{T\text{-}78G}$ (T-78G), as well as wild-type UTI89 (wt), UTI89Δ*ucl* (*ucl*) and UTI89-P*ucl*$^{G\text{-}78T}$. Ucl fimbriae were detected using UclA-specific polyclonal antibody. b) Control whole cell ELISA of wild-type F11 (wt), F11Δ*ucl* (*ucl*) and F11-P*ucl*$^{T\text{-}78G}$ (T-78G), as well as wild-type UTI89 (wt), UTI89Δ*ucl* (*ucl*) and UTI89-P*ucl*$^{G\text{-}78T}$ employing a general *E. coli* antibody (Life Research B65001R). The black dots show individual measurements for four technical replicates from three biological replicates (n=12); the grey bar indicates the mean. Statistical analyses were performed by one-way ANOVA with Sidak's multiple comparisons test.

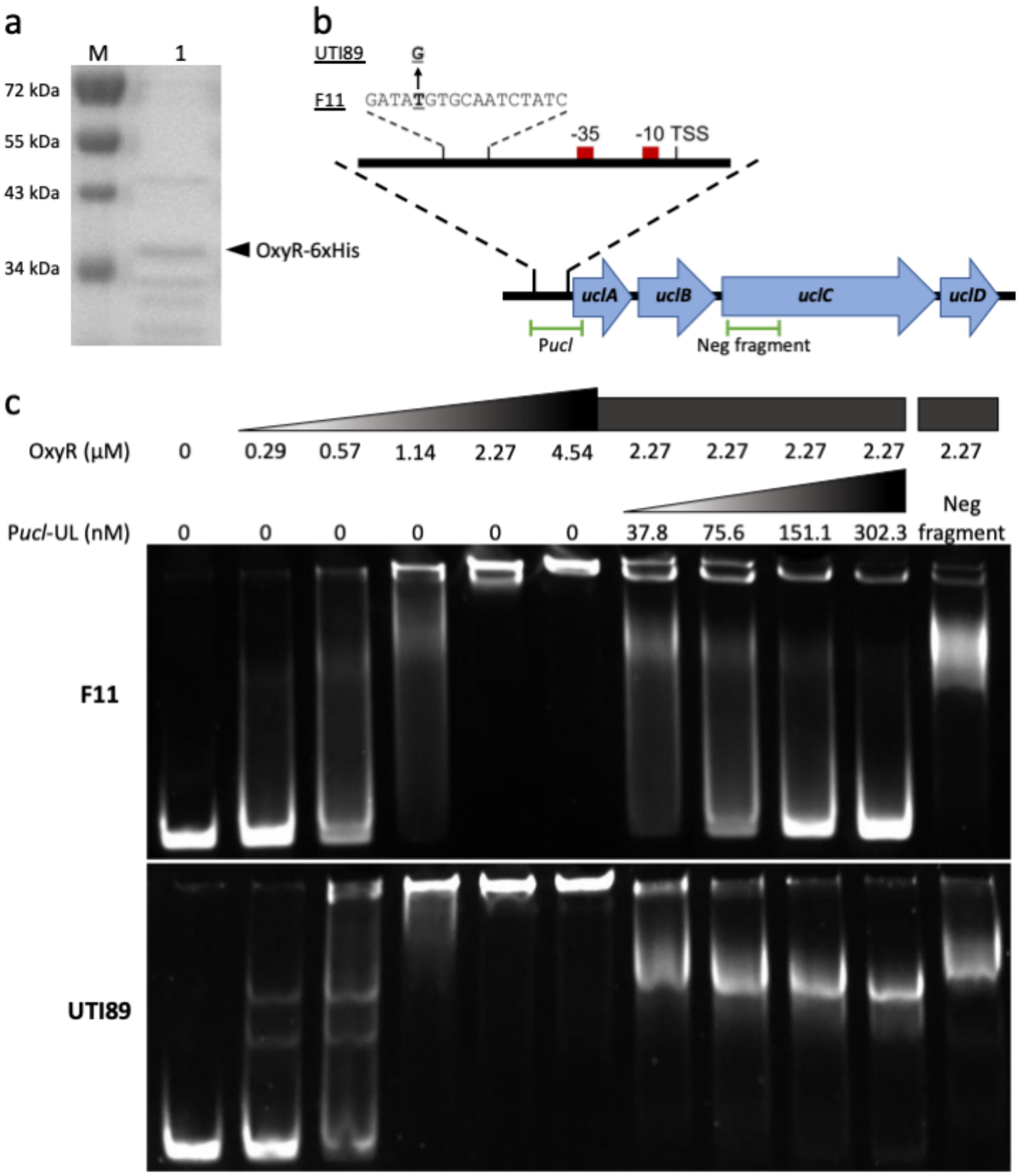

Fig H. Binding of OxyR to the *ucl* promoter region (P*ucl*). a, Coomassie-stained SDS-PAGE of OxyR-6xHis. Lane M: PageRuler Prestained Protein Ladder (Life Technologies, catalogue no. 26616), Lane 2: Nickel-affinity purified OxyR-6xHis. **b**, Schematic diagram of the *uclABCD* operon, indicating the transcription start site (TSS), -10 and -35 promoter region, and OxyR binding sequence containing the $T^{(-78)}$ in F11 and the $G^{(-78)}$ in UTI89. Also indicated are the 261 bp P*ucl* PCR fragment and the 240 bp *uclC* PCR fragment (negative control) used in the gel shift assay. **c**, Electrophoretic mobility shift assay of the Cy3-P*ucl*$^{-78T}$ (top) and Cy3-P*ucl*$^{-78G}$ (bottom) fragments with OxyR and increasing concentrations of unlabelled competitor (P*ucl*-UL) DNA.

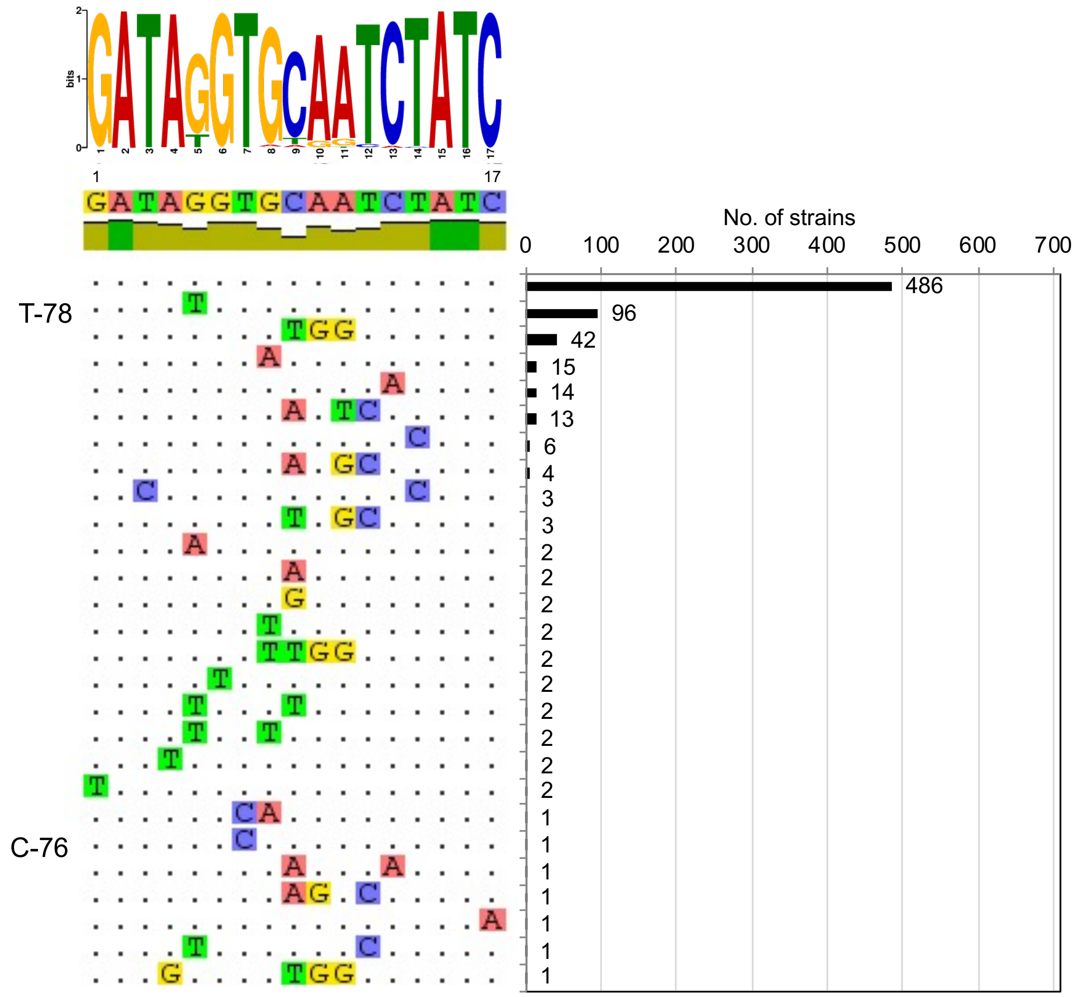

Fig I. Conservation of the P*ucl* OxyR binding site in ST127. A total of 845 genomes from ST127 strains on Enterobase were downloaded, 698 of which were positive for the Ucl fimbriae genes. The OxyR binding site was extracted from each P*ucl* sequence and aligned to generate the DNA logo shown at the top of the figure. Twenty-seven unique OxyR binding sites were identified, with nucleotide sequence changes shown below the consensus sequence. The number of times each unique sequence was identified in the dataset is indicated. The OxyR binding site consensus sequence was found most frequently (n=486), while the F11 T-78G SNP was also common (n=96). In total, 31% of the P*ucl* OxyR binding sites contained at least one SNP compared to the consensus sequence, with the F11 T-78G SNP most prevalent (14%). Sequences containing the G-78T and T-76C are indicated.

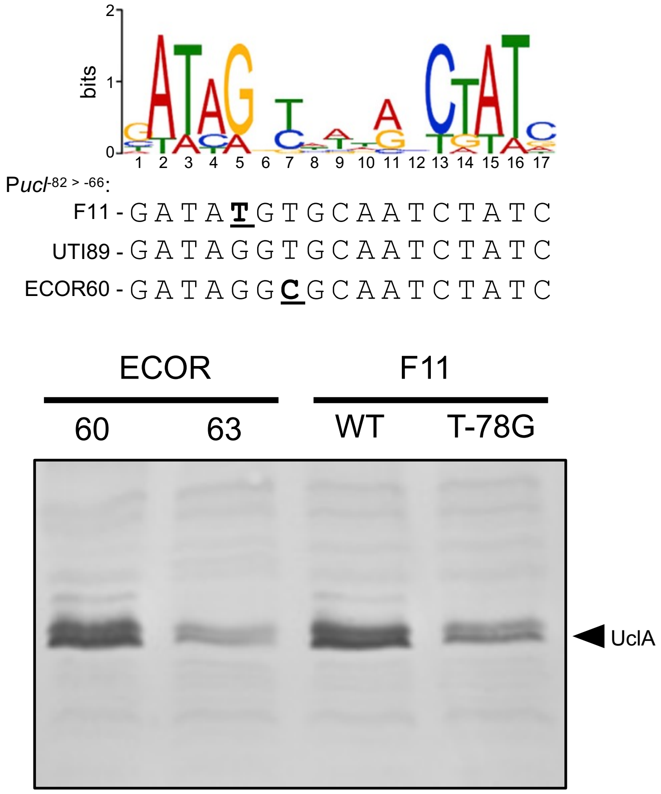

Fig J. UclA expression is increased in ECOR60. a) OxyR binding site sequences from UTI89 (consensus), F11 and ECOR60, highlighting the C-76T nucleotide sequence change in ECOR60. b) Whole-cell lysate western-blot analysis of ECOR60, ECOR63, F11 (WT) and F11-P*ucl*$^{T\text{-}78G}$ employing a UclA-specific antibody. Higher expression of UclA was observed in ECOR60 and F11 (WT) compared to ECOR63 and F11-P*ucl*$^{T\text{-}78G}$. The promoter region of the *ucl* operon from ECOR63 and UTI89 is identical.

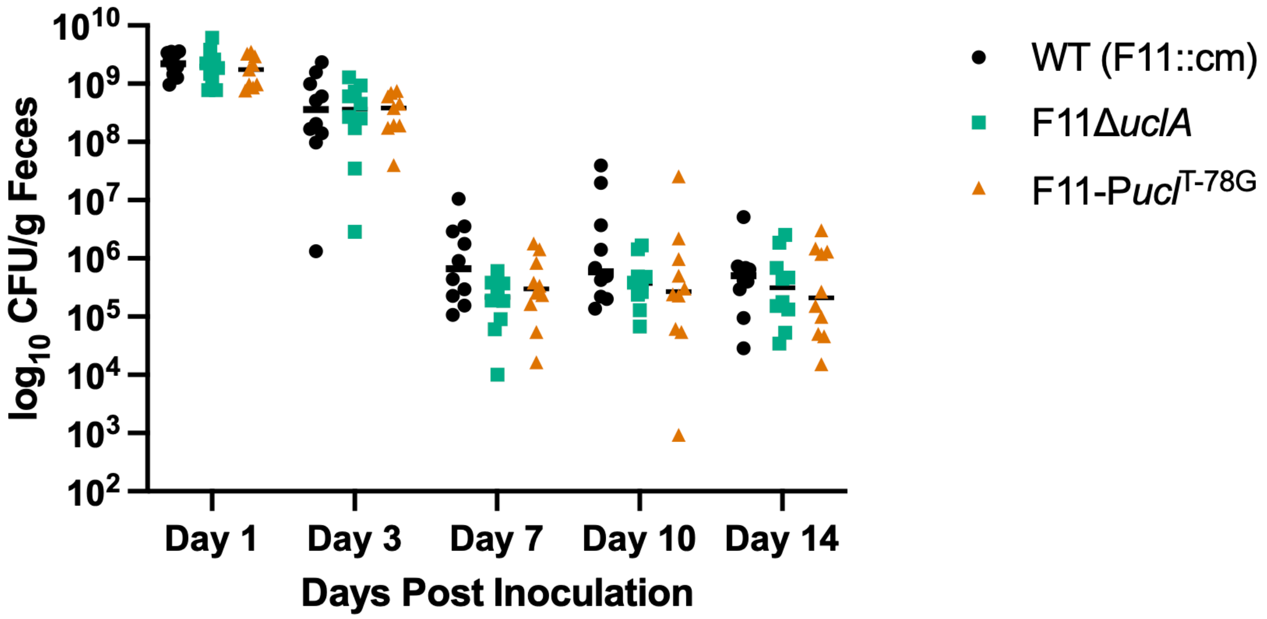

Fig K. Ucl fimbriae do not impact colonisation of the mouse gut in single infection experiments. Mice were inoculated wild-type F11 (tagged with a chloramphenicol resistance cassette; black circles), F11-P*ucl*$^{T-78G}$ (green squares) and an F11Δ*ucl* mutant (orange triangles). Each group contained 10-11 mice infected and monitored during two independent experiments. Bacterial loads were assessed over a 2-week period.

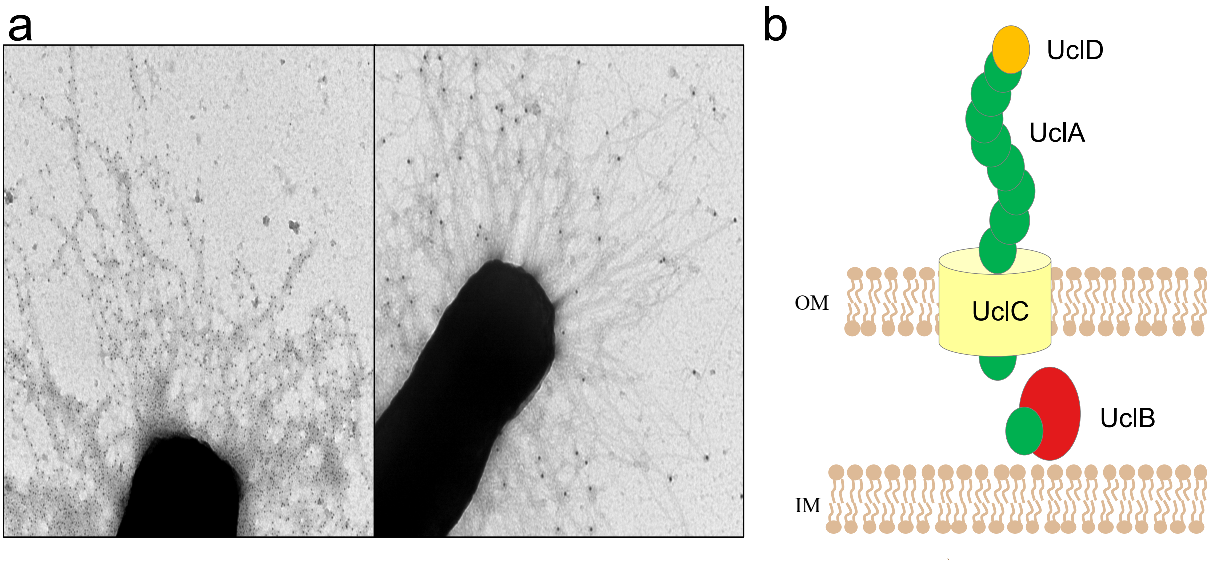

Fig L. Architecture of Ucl fimbriae demonstrated by co-immunogold labelled electron microscopy. a) Electron micrograph demonstrating immunogold labelled UclA major subunit (left; 5 nm gold particles) and UclD tip adhesin (right; 10 nm gold particles) of Ucl fimbriae. b) Cartoon model of Ucl fimbriae architecture, depicting the UclA major subunit repeating protein (green), UclD tip adhesin (orange), UclC usher (yellow) and UclB chaperone (red). Also labelled are the inner membrane (IM) and outer membrane (OM) of the cell

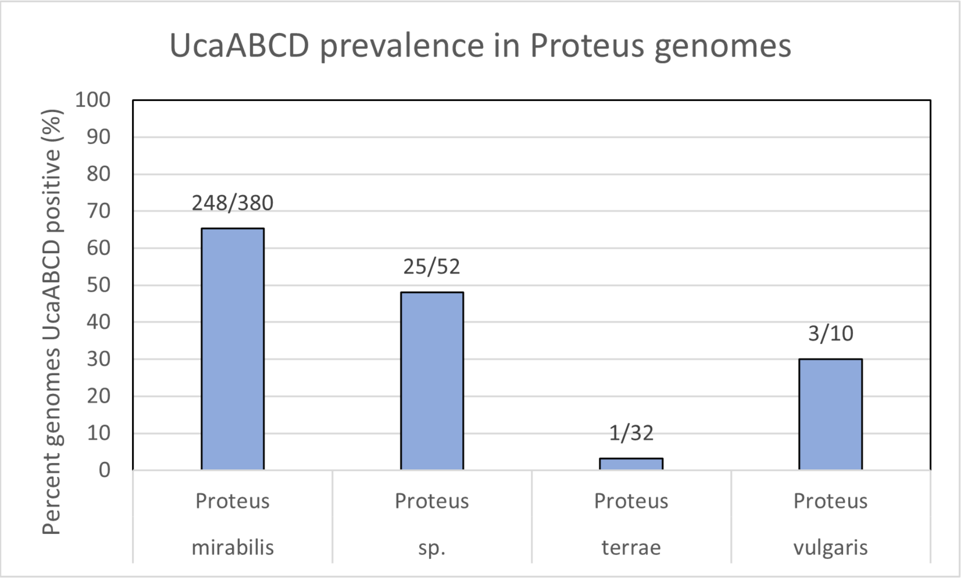

Fig M. Prevalence of the *uclABCD* genes in *Proteus* species. Genomes were assessed from the NCBI database. The analysis was performed using tblastn for UcaA, UcaB, UcaC and UcaD, with a positive result determined for blast hits with >70% identity and >80% coverage for all four proteins.

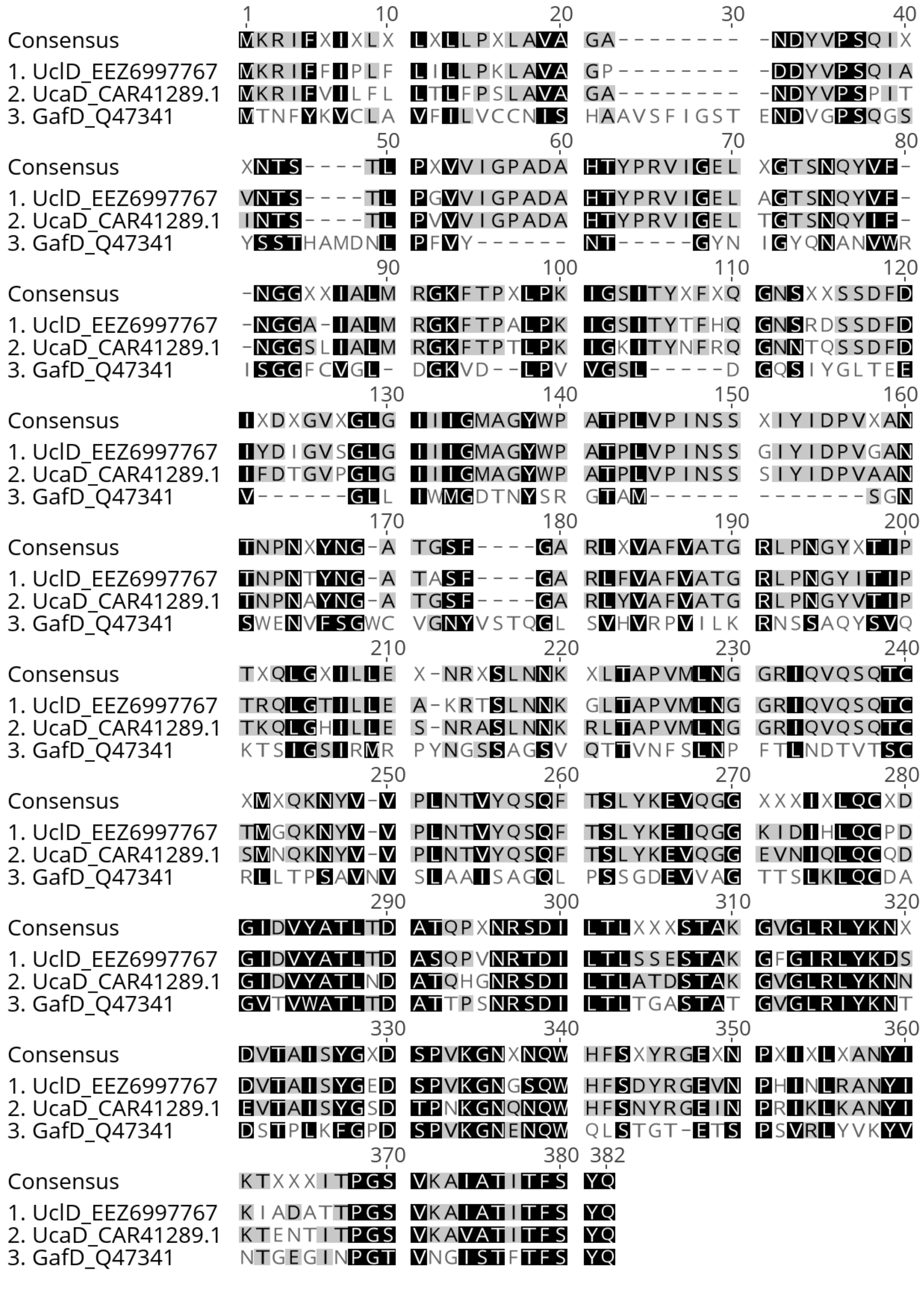

Fig N. Amino acid sequence alignment of the UclD (NCBI protein entry EEZ6997767), UcaD (CAR41289.1) and GafD (Q47341) adhesins. Identical amino acids are shaded in black; similar amino acids are shaded in grey. The consensus sequence is indicated.

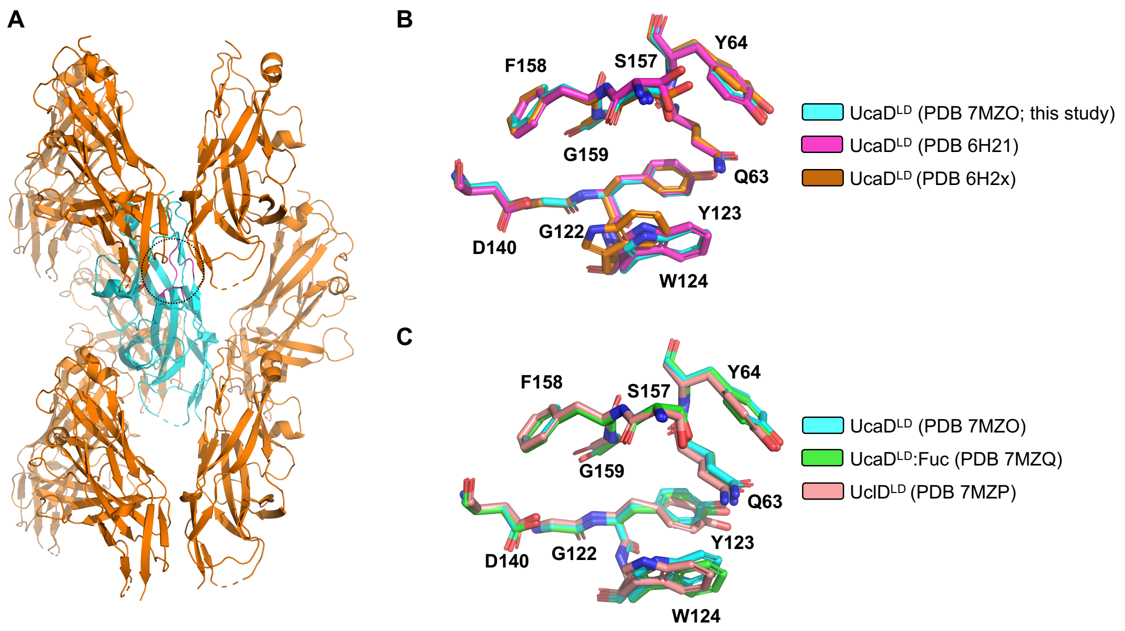

Fig O. A) Crystal packing analysis of UcaD^{LD} (PDB: 7MZO). The molecules are shown in cartoon representation. The asymmetric unit consists of one UcaD^{LD} molecule shown in cyan, while symmetry-related molecules are colored orange. The monosaccharide binding region of the asymmetric unit is circled and highlighted in magenta. **B)** Comparison of the monosaccharide binding site region in 3 different crystal forms of ligand-free UcaD^{LD}. **C)** Comparison of the monosaccharide binding site in the UcaD^{LD}:Fuc, UcaD^{LD} and UclD^{LD} crystal structures.

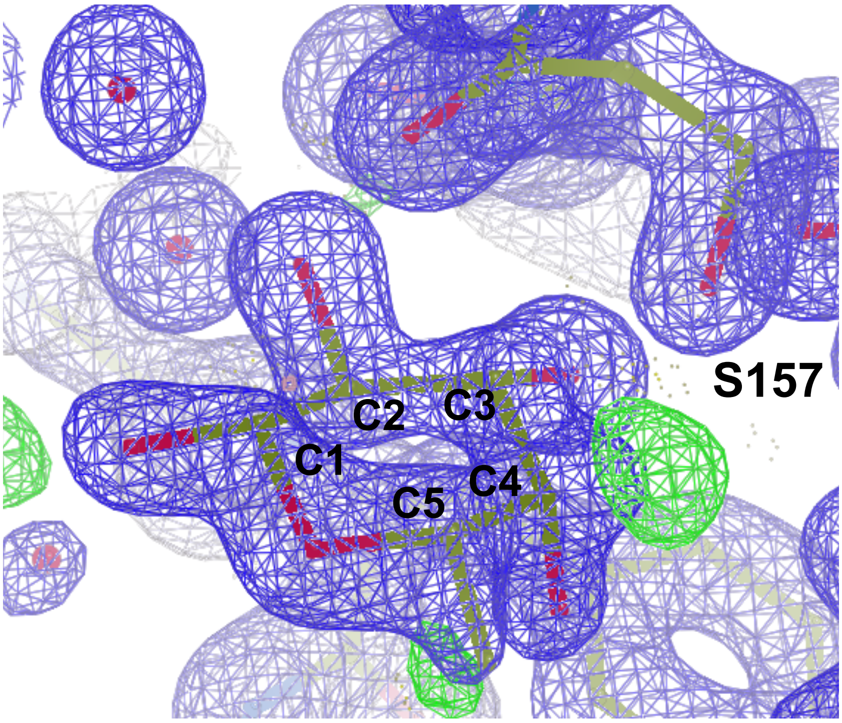

Fig P. Electron density map of the fucose binding site in the UcaD^{LD}:Fuc complex structure. Composite (2Fo - Fc, blue, contoured at 2σ) and difference (Fo - Fc, green/red, contoured at 3.0σ) electron density map of the Fuc binding site after refinement. Positive difference density adjacent to C4 suggests that a minor fraction of the Fuc molecules in the crystal adopts an alternate conformation.

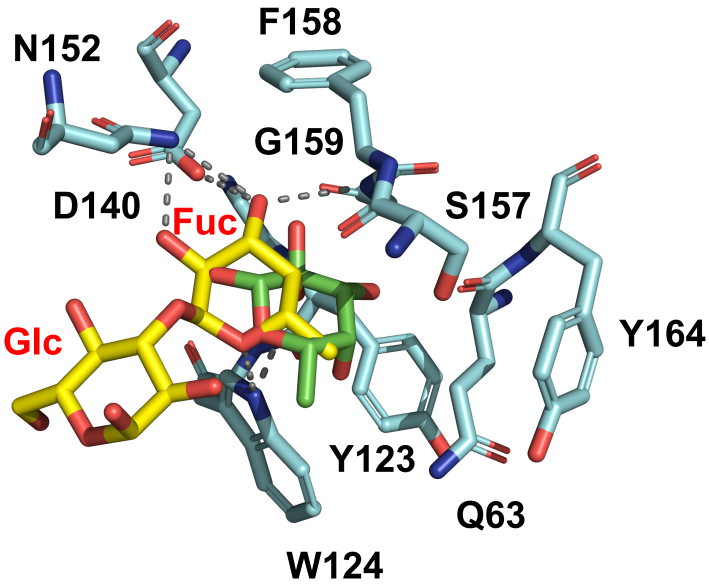

Fig Q. UcaD^{LD}:Fuc interactions in the MD derived structure. Interactions between the fucose residue of lacto-N-fucopentose VI (yellow) and residues of the binding pocket of ≤3.6 Å are shown as dashed lines. The binding model of the fucose molecule observed in the UcaD^{LD}:Fuc crystal structure is highlighted in green stick representation, for comparison.

Supplementary Tables

Table A. Crystallographic data.

	UclD^{LD} Dataset 1	UclD^{LD} Dataset 2	UcaD^{LD}	UcaD^{LD} Galactose	UcaD^{LD} Fucose	UcaD^{LD} Glucose
**Data collection**						
Detector	ADSC Quantum 210r CCD	ADSC Quantum 210r CCD	ADSC Quantum 315r CCD	ADSC Quantum 315r CCD	Dectris Eiger X 16M	Dectris Eiger X 16M
Wavelength (Å)	0.9537	1.3776	0.9537	0.9537	0.9537	0.9537
Temperature (K)	100	100	100	100	100	100
Rotation range per image (°)	0.5	0.5	0.5	0.5	0.1	0.1
Exposure time per image (s)	0.5	0.5	1	1	0.01	0.01
Space group	$P\ 2_12_12_1$	$P\ 2_12_12_1$	I 4	I 4	I 4	I 4
a, b, c (Å)	39.04, 58.12, 175.42	39.03, 58.26, 175.86	77.39, 77.39, 70.91	79.36, 79.36, 70.36	79.47, 79.47, 70.23	79.07, 79.07, 70.18
α, β, γ (°)	90.00, 90.00, 90.00	90.00, 90.00, 90.00	90.00, 90.00, 90.00	90.00, 90.00, 90.00	90.00, 90.00, 90.00	90.00, 90.00, 90.00
Average mosaicity (°) [b]	0.76	0.71	0.88	0.84	0.12	0.09
Resolution range (Å)	58.12-2.20 (2.27-2.20) [a]	87.93-2.85 (3.01-2.85) [a]	54.72-1.62 (1.65-1.62) [a]	56.12-1.72 (1.75-1.72) [a]	39.74-1.50 (1.53-1.50) [a]	39.54-1.78 (1.81-1.78) [a]
Total no. of reflections	147408 (12817)	259840 (33746)	185132 (9197)	83446 (4474)	475741 (21842)	283806 (13975)
No. of unique reflections	21160 (1794)	9966 (1412)	26588 (1305)	23071 (1237)	34590 (1653)	20791 (1137)
Completeness (%)	100.0 (100.0)	99.7 (99.6)	99.8 (99.9)	99.4 (99.7)	99.8 (95.7)	99.8 (97.0)
Multiplicity	7.0 (7.1)	26.1 (23.9)	7.0 (7.0)	3.6 (3.6)	13.8 (13.2)	13.7 (12.3)
Mean $I/\sigma(I)$	5.4 (1.5)	14.1 (6.1)	11.6 (1.4)	8.4 (1.5)	18.3 (2.5)	15.6 (3.3)
Rmeas (%) [c]	27.9 (143.7)	19.0 (50.9)	11.9 (155.9)	15.1 (131.9)	6.6 (69.4)	11.5 (58.4)
Rpim (%) [d]	10.5 (53.2)	3.7 (10.2)	4.5 (58.4)	7.7 (68.7)	1.8 (18.7)	3.1 (16.2)
$CC_{1/2}$ [b]	0.990 (0.748)	0.997 (0.985)	0.998 (0.634)	0.990 (0.291)	0.999 (0.895)	0.999 (0.887)
**Refinement**						
Resolution range (Å)	43.85-2.20		38.70-1.62	39.68-1.72	39.74-1.50	39.54-1.78
R_{work} (%) [e]	24.8		16.9	17.3	16.3	16.2
R_{free} (%) [f]	30.6		19.2	20.3	17.6	19.7
No. of non-H atoms						
Total	2920		1709	1689	1678	1683
Non-solvent	2785		1457	148	1483	1479
Water	129		247	236	195	204
Average isotropic B value (Å^2)	29.1		20.8	16.6	22.0	20.0
R.m.s.d. from ideal geometry						
Bond lengths (Å)	0.002		0.009	0.009	0.008	0.008
Bond angles (°)	0.553		0.957	0.970	0.936	0.936
Ramachadran plot, residues in (%)						
Favoured regions	96.19		97.35	97.31	97.35	97.34
Additionally allowed regions	3.81		2.65	2.69	2.65	2.66
Outlier regions	0		0	0	0	0

[a] The values in parentheses are for the highest-resolution shell.

[b] Calculated with AIMLESS [42].

[c] $R\text{meas} = \Sigma hkl\{N(hkl)/[N(hkl)\text{-}1]\}\,1/2\ \Sigma i|Ii(hkl)\text{-}\ \langle I(hkl)\rangle|/\ \Sigma hkl\Sigma iIi(hkl)$, where $Ii(hkl)$ is the intensity of the ith measurement of an equivalent reflection with indices hkl.

[d] $R\text{pim} = \Sigma hkl\{1/[N(hkl)\text{-}1]\}\,1/2\ \Sigma i|Ii(hkl)\text{-}\ \langle I(hkl)\rangle|/\ \Sigma hkl\Sigma iIi(hkl)$.

[e] $R_{work} = \sum_{hkl} ||F_{obs}|\text{-}|F_{calc}||/\sum_{hkl} |F_{obs}|$, where F_{obs} and F_{calc} are the observed and calculated structure factor amplitudes.

[f] R_{free} is equivalent to R_{work} but calculated with reflections (5-10%) omitted from the refinement process.

Table B. Polar interactions in the UcaD^{LD}: monosaccahride complexes[1].

	FUCOSE		GLUCOSE		GALACTOSE	
**PROTEIN ATOM**	Ligand atom	Distance (A)	Ligand atom	Distance (A)	Ligand atom	Distance (A)
**N63 NE2**	O4	3	O2	2.9	O5	3.3
**N63 NE2**	-	-	-	-	O6	2.9
**N63 O**	O3	2.7	O3	2.8	O1	2.7
**W124 NE1**	O5	3.2	O5	3.1	O4	2.8
**W124 NE1**	O4	3.1	-	-	-	-
**S157 OG**	-	-	O2	2.8	O1	2.8
**G159 N**	O3	3	O3	3.1	O1	3.4
**G159 N**	O2	2.9	O4	2.9	O2	2.8
**HOH1[2]**	O2	2.6	O4	2.6	O2	2.6

[1]Bond distances are based on the distances between nitrogen and oxygen atoms and do not include hydrogen atoms. [2]The water molecule is displayed in Figure 5.

Table C: List of strains and plasmids used in this study.

Strains or plasmids	Relevant description	Reference(s) or source
**Strains**		
MS528	*E. coli* K-12 MG1655Δ*fim*Δ*flu*	[30]
BL21(DE3) pLys		
F11	UPEC cystitis isolate	[30-32]
F11*ucl*	F11*uclA-D::cm*; Cm^r	This study
F11*lacI-Z*	F11*lacI-Z::gfp*	This study
F11*lacI-Z*-P*ucl::lacZ*	F11*lacI-Z::gfp Pucl::lacZ*	This study
F11-P*ucl*^{T78G}	F11-P*ucl*^{T78G}	This study
F11*lacI-Z*-P*ucl*^{T78G}*::lacZ*	F11*lacI-Z::gfp* P*ucl*^{T78G}*::lacZ*	This study
F11*oxyR*	F11*oxyR*	This study
F11-P*ucl*^{T78G} *oxyR*	F11-P*ucl*^{T78G} *oxyR*	This study
UTI89	UPEC cystitis isolate	[33, 34]
UTI89*ucl*	UTI89*uclA-D::cm*; Cm^r	This study
UTI89*lacI-Z*	UTI89*lacI-Z::gfp*	This study
UTI89-P*ucl*^{G78T}	UTI89-P*ucl*^{G78T}	This study
S77EC	Clinical ST131 isolate	[35]
S77ECΔ*ucl*	S77EC*uclA-D::cm*; Cm^r	This study
HVM1299	Clinical ST131 isolate	[35]
HVM1299*ucl*	HVM1299*uclA-D::cm*; Cm^r	This study
TOP10 pSU2718*::uclABCD*	*E. coli* TOP10 + pSU2718*::uclABCD* (pUcl)	This study
MS528 pSU2718::*uclABCD*	*E. coli* K-12 MG1655*fim,flu* pSU2718::*uclABCD* (pUcl)	This study
P. mirabilis PM54	UTI clinical isolate	[36]
**Plasmids**		
pKOBEG	λ-Red recombinase expression vector	[37]
pCP20	FLP flipase expression vector	[37]
pSU2718	pACYC184-derived cloning plasmid	[38]
pQF50	Promoterless *lacZ* reporter plasmid	[39]
pQF50-P*ucl*$_{F11}$	*ucl* promoter region from F11 cloned in pQF50	This study
pQF50-P*ucl*$_{UTI89}$	*ucl* promoter region from UTI89 cloned in pQF50	This study
pSU2718*::uclABCD* (pUcl)	*uclABCD* operon from F11 cloned into pSU2718	This study
pBAD/myc-HisA	Arabinose-inducible promoter, Amp^R	[40]
pOxyR	*oxyR* gene from *E. coli* MG1655 in pBAD/myc-HisA (pMGJ1)	[41]
pOxyR-6xHis	pOxyR modified to encode OxyR containing C-terminal 6xHistag	This study
pET22b	Expression vector, T7 promoter, N-ter pelB signal sequence, C-ter 6xHis tag, Amp^R	Novagen
pET22b::*uclD*	Lectin binding domain of *uclD* from F11 cloned into pET22b	This study
pET22b::*ucaD*	Lectin binding domain of *ucaD* from PM54 cloned into pET22b	This study

Table D: List of primers used in this study.

Primer name	Sequence (5' to 3')
**Mutant generation**	
6310-*ucl*:cm.1	tagcaagcgtagcgtaacag
6311-*ucl*:cm.2	cacagcagaaacaaatgtagcc
6312-*ucl*:cm.3	gcattggctacatttgtttctgctgtgtcctccttagttcctattcc
6313-*ucl*:cm.4	cacttccaggtgtagttgcatcagcaagtcttgagcgattgtgtagg
6314-*ucl*:cm.5	ttgctgatgcaactacacctg
6315-*ucl*:cm.6	tagtggctgcttccgtatcc
5080-*oxyR*.1	caatcttcctcgccagccca
5078-*oxyR*.2	gcaggtgatggtgtcagaa
3842-*oxyR*.3	ggaataggaactaaggaggatgccaccaggtactcaagatc
3843-*oxyR*.4	cctacacaatcgctcaagacaaagtgttaaaacaggcggt
5079-*oxyR*.5	aggtcaacagcatgtatcag
5081-*oxyR*.6	actgtctgctctatgccaa
4057-*lacIZ::cm-gfp* 700bp F	tcgtcttcatcctgctcttc
4058-*lacIZ::cm-gfp* 700bp R	gctaaatgccgaatggttg
7900_uclAprom_TtoG	a*t*ggtttttgatcgtttttcccgatcgttttagcaatcatgata**G**gtgcaatctatcatacgctggttatcgatcggtaaaaacattt*t*g
7901_uclAprom_GtoT	a*t*ggtttttgatcgtttttcccgatcgttttagcaatcatgata**T**gtgcaatctatcatacgctggttatcgatcggtaaaaacattt*t*g
**5' RACE**	
6973-P*uclA*-5'RACE GSP1	ctgagcactattcatacc
6974-P*uclA*-5'RACE GSP2	gaatggcaaagggtgtcag
**Cloning**	
6571-BamHI-P*ucl*-F	cgcgggatcccgggcatggatgttgtttat
6572-HindIII-P*ucl*-R	cccggaagctttccagtattgatcatttgttcagc
6568-*uclABCD*-F	cgcgggatccgttttgttggggatgattgc
6569-*uclABCD*-R	cccggtctagattattgatatgagaaagtaatagttg
11190_OxyR_cterGG6xHis_F	ggcggtcatcatcaccatcaccactgagtttaaacg
11191_OxyR_cterGG6xHis_R	aaccgcctgttttaaaactttatcg
**Gel shift assay**	
11206_PuclA-F2	aataggtcgctcatttcagg
11207_PuclA-F2-cy3	cy3-aataggtcgctcatttcagg
11208_PuclA-R2	cagcagaaacaaatgtagcc
11193_uclC-F	tcatgcctgatttactgtcg
11196_uclC-R2	atactgcaaatcagccttcc

Table E. Glycan array analysis of UclD and UcaD

Glycan structure	ID	UclD	UcaD
**Monosaccharides**			
Fucα-sp3	1	<1	<1
Galα-sp3	2	<1	<1
Galβ-sp3	3	<1	<1
GalNAcα-sp0	4	<1	<1
GalNAcα-sp3	5	<1	<1
GalNAcβ-sp3	6	<1	<1
Glcα-sp3	7	<1	<1
Glcβ-sp3	9	<1	<1
GlcNAcβ-sp3	10	<1	<1
GlcN(Gc)β-sp4	14	<1	<1
HOCH2(HOCH)4CH2NH2	15	<1	<1
Manα-sp3	16	<1	<1
Manβ-sp4	18	<1	<1
ManNAcβ-sp4	19	<1	<1
Rhaα-sp3	20	<1	<1
GlcNAcβ-sp4	22	<1	<1
3-O-Su-Galβ-sp3	37	<1	<1
3-O-Su-GalNAcα-sp3	38	<1	<1
6-O-Su-GlcNAcβ-sp3	43	<1	<1
GlcAα-sp3	44	<1	<1
GlcAβ-sp3	45	<1	<1
6-H2PO3Glcβ-sp4	46	<1	<1
6-H2PO3Manα-sp3	47	<1	<1
Neu5Acα-sp3	48	<1	<1
Neu5Acα-sp9	49	<1	<1
Neu5Gcα-sp3	52	<1	<1
9-NAc-Neu5Acα-sp3	54	<1	<1
3-O-Su-GlcNAcβ-sp3	55	<1	<1
**Terminal Galactose**			
Galα1-2Galβ-sp3	75	<1	<1
Galα1-3Galβ-sp3	76	<1	<1
Galα1-3GalNAcβ-sp3	77	<1	<1
Galα1-3GalNAcα-sp3	78	<1	<1
Galα1-3GlcNAcβ-sp3	80	<1	<1
Galα1-4GlcNAcβ-sp3	81	<1	<1
Galα1-6Glcβ-sp4	83	<1	<1
Galβ1-2Galβ-sp3	84	<1	<1
Galβ1-3GlcNaAcβ-sp3	85	<1	<1
Galβ1-3Galβ-sp3	87	<1	<1
Galβ1-3GalNAcβ-sp3	88	<1	<1
Galβ1-3GalNAcα-sp3	89	<1	<1
Galβ1-4Glcβ-sp4	93	<1	<1
Galβ1-4Galβ-sp4	94	<1	<1
Galβ1-4GlcNAcβ-sp3	97	<1	<1
Galβ1-6Galβ-sp4	100	<1	<1
Galβ1-3(6-O-Su)GlcNAcβ-sp3	145	<1	<1
Galβ1-4(6-O-Su)Glcβ-sp2	146	<1	<1

Galβ1-4(6-O-Su)GlcNAcβ-sp3	147	<1	<1
3-O-Su-Galβ1-3GalNAcα-sp3	150	<1	<1
6-O-Su-Galβ1-3GalNAcα-sp3	151	<1	<1
3-O-Su-Galβ1-4Glcβ-sp2	152	<1	<1
6-O-Su-Galβ1-4Glcβ-sp2	153	<1	<1
3-O-Su-Galβ1-3GlcNAcβ-sp3	155	<1	<1
3-O-Su-Galβ1-4GlcNAcβ-sp3	157	<1	<1
4-O-Su-Galβ1-4GlcNAcβ-sp3	159	<1	<1
6-O-Su-Galβ1-3GlcNAcβ-sp3	161	<1	<1
6-O-Su-Galβ1-4GlcNAcβ-sp3	163	<1	<1
3-O-Su-Galβ1-4(6-O-Su)Glcβ-sp2	176	<1	<1
3-O-Su-Galβ1-4(6-O-Su)GlcNAcβ-sp2	177	<1	<1
6-O-Su-Galβ1-4(6-O-Su)Glcβ-sp2	178	<1	<1
6-O-Su-Galβ1-3(6-O-Su)GlcNAcβ-sp2	179	<1	<1
6-O-Su-Galβ1-4(6-O-Su)GlcNAcβ-sp2	180	<1	<1
3,4-O-Su2-Galβ1-4GlcNAcβ-sp3	181	<1	<1
3,6-O-Su2-Galβ1-4GlcNAcβ-sp2	182	<1	<1
4,6-O-Su2-Galβ1-4GlcNAcβ-sp2	183	<1	<1
4,6-O-Su2-Galβ1-4GlcNAcβ-sp3	184	<1	<1
3,6-O-Su2-Galβ1-4(6-O-Su)GlcNAcβ-sp2	189	<1	<1
3,4-O-Su2-Galβ1-4GlcNAcβ-sp3	201	<1	<1
Galβ1-4(6-O-Su)GlcNAcβ-sp2	203	<1	<1
Galα1-3Galβ1-4Glcβ-sp2	220	<1	<1
Galα1-3Galβ1-4GlcNAcβ-sp3	222	<1	<1
Galα1-4Galβ1-4Glcβ-sp3	224	<1	<1
Galα1-4Galβ1-4GlcNAc-sp2	225	<1	<1
Galβ1-2Galα1-4GlcNAcβ-sp4	228	<1	<1
Galβ1-3Galβ1-4GlcNAcβ-sp4	229	<1	<1
Galβ1-4GlcNAcβ1-3GalNAcα-sp3	231	<1	5.882±1.12
Galβ1-4GlcNAcβ1-6GalNAcα-sp3	232	<1	<1
Galβ1-3(GlcNAcβ1-6)GalNAcα-sp3	254	<1	<1
Galβ1-3GalNAcβ1-3Gal-sp4	262	<1	4.944±0.98
Galβ1-4Galβ1-4GlcNAc-sp3	264	<1	<1
Galα1-3Galβ1-4GlcNAcβ1-3Galβ-sp3	373	<1	<1
Galα1-4GlcNAcβ1-3Galβ1-4GlcNAcβ-sp3	375	<1	<1
Galβ1-3GlcNAcβ1-3Galβ1-4Glcβ-sp4	376	<1	<1
Galβ1-3GlcNAcβ1-3Galβ1-3GlcNAcβ-sp2	377	<1	<1
Galβ1-3GlcNAcα1-3Galβ1-4GlcNAcβ-sp3	378	<1	<1
Galβ1-3GlcNAcβ1-3Galβ1-4GlcNAcβ-sp3	379	<1	<1
Galβ1-3GlcNAcα1-6Galβ1-4GlcNAcβ-sp2	380	<1	<1
Galβ1-3GlcNAcβ1-6Galβ1-4GlcNAcβ-sp2	381	<1	<1
Galβ1-3GalNAcβ1-4Galβ1-4Glcβ-sp3	382	<1	<1
Galβ1-4GlcNAcβ1-3Galβ1-4Glcβ-sp2	383	<1	<1
Galβ1-4GlcNAcβ1-3Galβ1-4GlcNAcβ-sp3	385	<1	<1
Galβ1-4GlcNAcβ1-6Galβ1-4GlcNAcβ-sp2	387	<1	<1
Galβ1-4GlcNAcβ1-6(Galβ1-3)GalNAcα-sp3	388	<1	<1
Galβ1-4GlcNAcβ1-6(Galβ1-4GlcNAcβ1-3)GalNAcα-sp3	488	<1	5.566±1.08
Galβ1-4GlcNAcβ1-3(Galβ1-4GlcNAcβ1-6)GalNAcα-sp3	504	<1	<1
Galβ1-3GlcNAc	1A	<1	<1
Galβ1-4GlcNAc	1B	<1	<1
Galβ1-4Gal	1C	<1	2.886±0.77
Galβ1-6GlcNAc	1D	<1	<1

Galβ1-3GalNAc	1E	<1	<1
Galβ1-3GalNAcβ1-4Galβ1-4Glc	1F	<1	<1
Galβ1-3GlcNAcβ1-3Galβ1-4Glc	1G	<1	<1
Galβ1-4GlcNAcβ1-3Galβ1-4Glc	1H	<1	<1
Galβ1-4GlcNAcβ1-6(Galβ1-4GlcNAcβ1-3)Galβ1-4Glc	1I	<1	<1
Galβ1-4GlcNAcβ1-6(Galβ1-3GlcNAcβ1-3)Galβ1-4Glc	1J	<1	<1
Gala1-4Galβ1-4Glc	1K	<1	<1
GalNAcα1-O-Ser	1L	<1	<1
Galb1-3GalNAcα1-O-Ser	1M	<1	<1
Gala1-3Gal	1N	<1	<1
Gala1-3Galβ1-4GlcNAc	1O	<1	<1
Gala1-3Galβ1-4Glc	1P	<1	<1
Gala1-3Galβ1-4Galα1-3Gal	2A	<1	<1
Galβ1-6Gal	2B	<1	<1
GalNAcβ1-3Gal	2C	<1	<1
GalNAcβ1-4Gal	2D	<1	<1
Galα1-4Galβ1-4GlcNAc	2E	<1	<1
GalNAcα1-3Galβ1-4Glc	2F	<1	<1
Galβ1-3GlcNAcβ1-3Galβ1-4GlcNAcβ1-6(Galβ1-3GlcNAcβ1-3)Galβ1-4Glc	2G	<1	<1
Galβ1-3GlcNAcβ1-3Galβ1-4GlcNAcβ1-3Galβ1-4Glc	2H	<1	<1
Galβ1-3GalNAcβ1-3Galα1-4Galβ1-4Glc	18B	<1	<1
Galβ1-3GalNAcβ1-3Gal	18C	<1	<1
Galβ1-4Glc	18L	<1	<1
Galβ1-4Gal	18M	<1	<1
Galβ1-6Gal	18N	<1	<1
**Terminal N-Acetylgalactosamine**			
GalNAcα1-3GalNAcβ-sp3	101	<1	<1
GalNAcα1-3Galβ-sp3	102	<1	<1
GalNAcα1-3GalNAcα-sp3	103	<1	<1
GalNAcβ1-3Galβ-sp3	104	<1	<1
GalNAcβ1-4GlcNAcβ-sp3	106	<1	<1
GalNAcβ1-4(6-O-Su)GlcNAcβ-sp3	192	<1	<1
3-O-Su-GalNAcβ1-4GlcNAcβ-sp3	193	<1	<1
6-O-Su-GalNAcβ1-4GlcNAcβ-sp3	194	<1	<1
6-O-Su-GalNAcβ1-4-(3-O-Su)GlcNAcβ-sp3	195	<1	<1
3-O-Su-GalNAcβ1-4(3-O-Su)-GlcNAcβ-sp3	196	<1	<1
3,6-O-Su$_2$-GalNAcβ1-4GlcNAcβ-sp3	197	<1	<1
4,6-O-Su$_2$-GalNAcβ1-4GlcNAcβ-sp3	198	<1	<1
4,6-O-Su$_2$-GalNAcβ1-4-(3-O-Ac)GlcNAcβ-sp3	199	<1	<1
4-O-Su-GalNAcβ1-4GlcNAcβ-sp3	200	<1	<1
6-O-Su-GalNAcβ1-4(6-O-Su)GlcNAcβ-sp3	202	<1	<1
4-O-Su-GalNAcβ1-4GlcNAcβ-sp2	204	<1	<1
GalNAcβ1-4Galβ1-4Glcβ-sp3	238	<1	<1
GalNAcβ1-3Galα1-4Galβ1-4Glcβ-sp3	389	<1	<1
GalNAcα1-O-Ser	1L	<1	<1
GalNAcβ1-3Gal	2C	<1	<1
GalNAcβ1-4Gal	2D	<1	<1
GalNAcα1-3Galβ1-4Glc	2F	<1	<1
**Terminal N-Acetylglucosamine**			
GlcNAcβ1-3GalNAcα-sp3	113	<1	<1
GlcNAcβ1-3Manβ-sp4	114	<1	<1

GlcNAcβ1-4GlcNAcβ-Asn	115	<1	<1
GlcNAcβ1-4GlcNAcβ-sp4	117	<1	<1
GlcNAcβ1-6GalNAcα-sp3	118	<1	<1
GlcNAcβ1-4(6-O-Su)GlcNAcβ-sp2	149	<1	<1
GlcNAcβ1-4-[HOOC(CH_3)CH]-3-O-GlcNAcβ-sp4	167	<1	<1
GlcNAcβ1--[HOOC(CH_3)CH]-3-O-GlcNAcβ-L-alanyl-D-i-glutaminyl-L-lysine	168	<1	<1
GlcNAcβ1-2Galβ1-3GalNAcα-sp3	246	<1	<1
GlcNAcβ1-3Galβ1-3GalNAcα-sp3	247	<1	<1
GlcNAcβ1-3Galβ1-4Glcβ-sp2	248	<1	<1
GlcNAcβ1-3Galβ1-4GlcNAcβ-sp3	250	<1	<1
GlcNAcβ1-4Galβ1-4GlcNAcβ-sp2	251	<1	<1
GlcNAcβ1-4GlcNAcβ1-4GlcNAcβ-sp4	252	<1	<1
GlcNAcβ1-6Galβ1-4GlcNAcβ-sp2	253	<1	<1
GlcNAcβ1-3(GlcNAcβ1-6)GalNAcα-sp3	255	<1	<1
GlcNAcβ1-3(GlcNAcβ1-6)Galβ1-4GlcNAcβ-sp3	395	<1	<1
(GlcNAcβ1-4)$_5$β-sp4	493	<1	<1
(GlcNAcβ1-4)$_6$β-sp4	503	<1	<1
(GN-M)$_2$-3,6-M-GN-GNβ-sp4	505	<1	<1
Glc*N*Acβ1-4Glc*N*Ac	4A	<1	<1
Glc*N*Acβ1-4Glc*N*Acβ1-4Glc*N*Ac	4B	<1	<1
Glc*N*Acβ1-4Glc*N*Acβ1-4Glc*N*Acβ1-4Glc*N*Ac	4C	<1	<1
Glc*N*Acβ1-4Glc*N*Acβ1-4Glc*N*Acβ1-4Glc*N*Acβ1-4Glc*N*Acβ1-4Glc*N*Ac	4D	<1	<1
Bacterial cell wall muramyl discaccharide	4E	<1	<1
GlcNAcβ1-4GlcNAcβ1-4GlcNAcβ1-4GlcNAcβ1-4GlcNAc	4F	<1	<1
**Mannose**			
Manα1-2Manβ-sp4	119	<1	<1
Manα1-3Manβ-sp4	120	<1	<1
Manα1-4Manβ-sp4	121	<1	<1
Manα1-6Manβ-sp4	122	<1	<1
Manβ1-4GlcNAcβ-sp4	123	<1	<1
Manα1-2Manα-sp4	124	<1	<1
Manα1-3(Manα1-6)Manβ-sp4	258	<1	<1
Manα1-3(Manα1-3(Manα1-6)Manα1-6)Manβ-sp4	495	<1	<1
Glc*N*Acβ1-2Man	5A	<1	<1
Glc*N*Acβ1-2Manα1-6(Glc*N*Acβ1-2Manα1-3)Man	5B	<1	<1
Manα1-2Man	5C	<1	<1
Manα1-3Man	5D	<1	<1
Manα1-4Man	5E	<1	<1
Manα1-6Man	5F	<1	<1
Manα1-6(Manα1-3)Man	5G	<1	<1
Manα1-6(Manα1-3)Manα1-6(Manα1-3)Man	5H	<1	<1
**Fucosylated**			
Fucα1-2Galβ-sp3	71	<1	<1
Fucα1-3GlcNAcβ-sp3	72	<1	<1
Fucα1-4GlcNAcβ-sp3	73	<1	<1
Fucα1-2Galβ1-3GlcNAcβ-sp3	215	<1	<1
Fucα1-2Galβ1-4GlcNAcβ-sp3	216	<1	<1
Fucα1-2Galβ1-3GalNAcα-sp3	217	<1	<1
Fucα1-2Galβ1-4Glcβ-sp4	219	<1	<1
Fucα1-2(Galα1-3)Galβ-sp3	226	<1	<1

Galβ1-3(Fucα1-4)GlcNAcβ-sp3	233	<1	<1
Fucα1-3(Galβ1-4)GlcNAcβ-sp3	234	<1	<1
Fucα1-2(GalNAcα1-3)Galβ-sp3	235	<1	<1
3-O-Su-Galβ1-3(Fucα1-4)GlcNAcβ-sp3	287	<1	<1
Fucα1-3(3-O-Su-Galβ1-4)GlcNAcβ-sp3	288	<1	<1
Fucα1-2(Galα1-3)Galβ1-3GlcNAcβ-sp3	359	<1	<1
Fucα1-2(Galα1-3)Galβ1-4GlcNAcβ-sp3	360	<1	<1
Fucα1-2(Galα1-3)Galβ1-3GalNAcα-sp3	362	<1	<1
Fucα1-2(Galα1-3)Galβ1-3GalNAcβ-sp3	363	<1	<1
Fucα1-3(Galα1-3Galβ1-4)GlcNAcβ-sp3	364	<1	<1
Fucα1-2(GalNAcα1-3)Galβ1-3GlcNAcβ-sp3	366	<1	<1
Fucα1-2(GalNAcα1-3)Galβ1-4GlcNAcβ-sp3	368	<1	<1
Fucα1-2Galβ1-3(Fucα1-4)GlcNAcβ-sp3	371	<1	<1
Fucα1-3(Fucα1-2Galβ1-4)GlcNAcβ-sp3	372	<1	<1
Fucα1-2(GalNAcα1-3)Galβ1-3GalNAcα-sp3	392	<1	<1
Fucα1-2Galβ1-3GlcNAcβ1-3Galβ1-4Glcβ-sp4	479	<1	<1
Fucα1-2Galβ1-3GlcNAcβ1-3Galβ1-4GlcNAcβ-sp2	480	<1	<1
Fucα1-3(Fucα1-2 (Galα1-3)Galβ1-4)GlcNAcβ-sp3	483	<1	<1
Fucα1-2Galβ1-3(Fucα1-4)GlcNAcβ1-3Galβ1-4Glcβ-sp4	496	<1	<1
Fucα1-3(Fucα1-2Galβ1-4)GlcNAcβ1-3Galβ1-4Glcβ-sp4	497	<1	<1
Lex1-6'(Lec1-3')Lac-sp4	538	<1	<1
LacNAc1-6'(Led1-3')Lac-sp4	539	<1	<1
Lex1-6'(Led1-3')Lac-sp4	541	<1	<1
LecLex1-6'(Lec1-3')Lac-sp4	542	<1	<1
Lex1-6'(Leb1-3')Lac-sp4	543	<1	<1
Fucα1-2Galβ1-3GlcNAcβ1-3Galβ1-4Glc	7A	<1	1.992±0.43
Galβ1-3(Fuca1-4)GlcNAcβ1-3Galβ1-4Glc	7B	<1	<1
Galβ1-4(Fuca1-3)GlcNAcβ1-3Galβ1-4Glc	7C	<1	<1
Fucα1-2Galβ1-3(Fucα1-4)GlcNAcβ1-3Galβ1-4Glc	7D	<1	<1
Galβ1-3(Fucα1-4)GlcNAcβ1-3Galβ1-4(Fucα1-3)Glc	7E	<1	<1
Fucα1-2Gal	7F	<1	<1
Fucα1-2Galβ1-4Glc	7G	<1	<1
Galβ1-4(Fucα1-3)Glc	7H	<1	<1
Galβ1-4(Fucα1-3)GlcNAc	7I	<1	<1
Galβ1-3(Fucα1-4)GlcNAc	7J	<1	<1
GalNAcα1-3(Fucα1-2)Gal	7K	<1	<1
Fucα1-2Galβ1-4(Fucα1-3)Glc	7L	<1	<1
Galβ1-3(Fucα1-2)Gal	7M	<1	<1
Fucα1-2Galβ1-4(Fucα1-3)GlcNAc	7N	<1	<1
Fucα1-2Galβ1-3GlcNAc	7O	<1	<1
Fucα1-2Galβ1-3(Fucα1-4)GlcNAc	7P	<1	<1
SO3-3Galβ1-3(Fucα1-4)GlcNAc	8A	<1	<1
SO3-3Galβ1-4(Fucα1-3)GlcNAc	8B	<1	<1
Galβ1-3GlcNAcβ1-3Galβ1-4(Fucα1-3)GlcNAcβ1-3Galβ1-4Glc	8C	<1	<1
Galβ1-4(Fucα1-3)GlcNAcβ1-6(Galβ1-3GlcNAcβ1-3)Galβ1-4Glc	8D	<1	<1
Galβ1-4(Fucα1-3)GlcNAcβ1-6(Fucα1-2Galβ1-3GlcNAcβ1-3)Galβ1-4Glc	8E	<1	<1
Galβ1-4(Fucα1-3)GlcNAcβ1-6(Fucα1-2Galβ1-3(Fucα1-4)GlcNAcβ1-3)Galβ1-4Glc	8F	<1	<1
Galβ1-4GlcNAcβ1-3Galβ1-4(Fucα1-3)Glc	8G	4.877±0.33	3.611±0.49

Fucα1-2Galβ1-4(Fucα1-3)GlcNAcβ1-3Galβ1-4Glc	8H	<1	<1
Fucα1-3Galβ1-4GlcNAcβ1-3Galβ1-4(Fucα1-3)Glc	8I	<1	<1
Fuca1-2Galb1-4(Fuca1-3)GlcNAcb1-3(Fuca1-2)Galb1-4Glc	8J	<1	<1
Galβ1-4(Fucα1-3)GlcNAcβ1-6(Galβ1-4GlcNAcβ1-3)Galβ1-4Glc	8K	<1	<1
Galb1-4(Fuca1-3)GlcNAcb1-6(Galb1-4(Fuca1-3)GlcNAcb1-3)Galb1-4Glc	8L	<1	<1
Fuca1-2Galb1-4(Fuca1-3)GlcNAcb1-6(Galb1-4GlcNAcb1-3)Galb1-4Glc	8M	<1	<1
Galb1-3GlcNAcb1-3Galb1-4(Fuca1-3)GlcNAcb1-6(Galb1-3GlcNAcb1-3)Galb1-4Glc	8N	<1	<1
Fucα1-2Galβ1-3GlcNAcβ1-3Galβb1-4(Fucα1-3)GlcNAcβ1-6(Galβ1-3GlcNAcβ1-3)Galβ1-4Glc	8O	<1	<1
GalNAcb1-3(Fuca1-2)Galb1-4Glc	8P	<1	<1
Galb1-3(Fuca1-2)Galb1-4(Fuca1-3)Glc	9A	<1	<1
Galβ1-4GlcNAcβ1-6(Fucα1-2Galβ1-3GlcNAcβ1-3)Galβ1-4Glc	9B	<1	<1
Galα1-3(Fucα1-2)Galβ1-4Glc	18D	<1	<1
**Sialylated**			
Neu5Acα2-3Galβ-sp3	169	<1	<1
Neu5Acα2-6Galβ-sp3	170	<1	<1
Neu5Acα2-3GalNAcα-sp3	171	<1	<1
Neu5Acα2-6GalNAcα-sp3	172	<1	<1
Neu5Gcα2-6GalNAcα-sp3	174	<1	<1
Neu5Acα2-8Neu5Acα2-sp3	186	<1	<1
Neu5Acα2-6GalNAcβ-sp3	205	<1	<1
Neu5Gcα2-3Gal-sp3	206	<1	<1
Galα1-3(Neu5Acα2-6)GalNAcα-sp3	289	<1	<1
Galβ1-3(Neu5Acα2-6) GalNAcα-sp3	290	<1	<1
Neu5Acα2-3Galβ1-3GalNAcα-sp3	292	<1	<1
Neu5Acα2-3Galβ1-4Glcβ-sp3	293	<1	<1
Neu5Acα2-3Galβ1-4Glcβ-sp4	294	<1	<1
Neu5Acα2-6Galβ1-4Glcβ-sp2	295	<1	<1
Neu5Acα2-3Galβ1-4GlcNAcβ-sp3	298	<1	<1
Neu5Acα2-3Galβ1-3GlcNAcβ-sp3	299	<1	<1
Neu5Acα2-6Galβ1-4GlcNAcβ-sp3	300	<1	<1
Neu5Gcα2-3Galβ1-4GlcNAcβ-sp3	303	<1	<1
Neu5Gcα2-6Galβ1-4GlcNAcβ-sp3	304	<1	<1
9-NAc-Neu5Acα2-6Galβ1-4GlcNAcβ-sp3	306	<1	<1
Neu5Acα2-3Galβ1-4-(6-O-Su)GlcNAcβ-sp3	315	<1	<1
Neu5Acα2-3Galβ1-3-(6-O-Su)GalNAcβ-sp3	317	<1	<1
Neu5Acα2-6Galβ1-4-(6-O-Su)GlcNAcβ-sp3	318	<1	<1
Neu5Acα2-3-(6-O-Su)Galβ1-4GlcNAcβ-sp3	319	<1	1.224±0.38
(Neu5Acα2-8)3-sp3	321	<1	<1
Neu5Acα2-6Galβ1-3GlcNAc-sp3	323	<1	<1
Neu5Acα2-6Galβ1-3(6-O-Su)GlcNAc-sp3	324	<1	<1
Neu5Gcα2-3Galβ1-3GlcNAcβ-sp3	331	<1	<1
Neu5Acα2-3(GalNAcβ1-4)Galβ1-4Glcβ-sp2	421	<1	<1
Neu5Acα2-3Galβ1-4GlcNAcβ1-3Galβ-sp3	422	<1	<1
Fucα1-3(Neu5Acα2-3Galβ1-4)GlcNAcβ-sp3	423	<1	<1
Neu5Acα2-3Galβ1-3(Fucα1-4)GlcNAcβ-sp3	426	<1	<1
Fucα1-3(Neu5Acα2-3Galβ1-4)6-O-Su-GlcNAcβ-sp3	428	<1	<1

Fucα1-3(Neu5Acα2-3(6-O-Su)Galβ1-4)GlcNAcβ-sp3	429	<1	6.012±1.14
Neu5Acα2-3Galβ1-3(Neu5Acα2-6)GalNAcα-sp3	433	<1	<1
Neu5Acα2-8Neu5Acα2-3Galβ1-4Glcβ-sp4	434	<1	<1
Neu5Acα2-3Galβ1-4GlcNAcβ1-3Galβ1-4GlcNAcβ-sp2	527	<1	<1
Fucα1-3(Neu5Acα2-3 Galβ1-4)GlcNAcβ1-3Galβ-sp3	528	<1	<1
Neu5Acα2-6(Galβ1-3)GlcNAcβ1-3Galβ1-4Glcβ-sp4	529	<1	<1
GalNAcβ1-4(Neu5Acα2-8Neu5Acα2-3)Galβ1-4Glc-sp2	531	<1	<1
Neu5Acα2-8Neu5Acα2-8Neu5Acα2-3Galβ1-4Glc-sp2	532	<1	6.882±1.44
(Neu5Acα2-8)2Neu5Acα2-3(GalNAcβ1-4)Galβ1-4Glc-sp2	533	<1	<1
Neu5Acα2-3Galβ1-4GlcNAcβ1-3Galβ1-4GlcNAcβ-sp3	534	<1	<1
Neu5Acα2-3Galβ1-3GlcNAcβ1-3Galβ1-4Glcβ-sp4	536	<1	<1
Neu5Acα2-3Galβ1-4GlcNAcβ1-3Galβ1-4Glcβ-sp4	537	<1	<1
Lex1-6'(6'SLN1-3')Lac-sp4	540	<1	<1
Neu5Acα2-3Galβ1-3(Fucα1-4)GlcNAc	10A	<1	2.437±0.58
Neu5Acα2-3Galβ1-4(Fucα1-3)GlcNAc	10B	1.211±0.38	<1
Neu5Acα2-3Galβ1-3GlcNAcβ1-3Galβ1-4Glc	10C	<1	<1
Galβ1-4(Fucα1-3)GlcNAcβ1-6(Neu5Acα2-6Galβ1-4GlcNAcβ1-3)Galβ1-4Glc	10D	<1	<1
Neu5Acα2-3Galβ1-3(Neu5Acα2-6)GalNAc	10E	<1	<1
Neu5Acα2-6Galβ1-3GlcNAcβ1-3Galβ1-4(Fucα1-3)Glc	10H	6.445±0.84	<1
Galβ1-3GlcNAcβ1-3(Neu5Acα2-6Galβ1-4GlcNAcβ1-6)Galβ1-4Glc	10I	<1	<1
Neu5Acα2-6Galβ1-3GlcNAcβ1-3(Galβ1-4GlcNAcβ1-6)Galβ1-4Glc	10J	<1	<1
Neu5Acα2-3Galβ1-4GlcNAc	10K	<1	<1
Neu5Acα2-6Galβ1-4GlcNAc	10L	<1	<1
Neu5Acα2-3Galβ1-3GlcNAcβ1-3Galβ1-4Glc	10M	<1	<1
Galβ1-3(Neu5Acα2-6)GlcNAcβ1-3Galβ1-4Glc	10N	<1	<1
Neu5Acα2-6Galβ1-4GlcNAcβ1-3Galβ1-4Glc	10O	1.291±0.64	<1
Neu5Acα2-3Galβ1-3(Neu5Acα2-6)GlcNAcβ1-3Galβ1-4Glc	10P	<1	<1
Neu5Acα2-3Galβ1-4Glc	11A	<1	<1
Neu5Acα2-6Galβ1-4Glc	11B	<1	<1
(Neu5Acα2-8Neu5Ac)n (n<50)	11C	<1	<1
**Glucose**			
Glcα1-4Glcβ-sp3	110	<1	<1
Glcβ1-4Glcβ-sp4	111	<1	<1
Glcβ1-6Glcβ-sp4	112	<1	<1
GlcAβ1-3GlcNAcβ-sp3	164	<1	<1
GlcAβ1-3Galβ-sp3	165	<1	<1
GlcAβ1-6Galβ-sp3	166	<1	<1
(Glcα1-4)3β-sp4	240	<1	<1
(Glcα1-6)3β-sp4	241	<1	<1
(Glcα1-4)4β-sp4	390	<1	<1
(Glcα1-6)4β-sp4	391	<1	<1
(Glcα1-6)5β-sp4	492	<1	<1
(Glcα1-6)6β-sp4	502	<1	<1
GlcA	18I	<1	<1
6-O-(H2PO4)-Glc	18J	<1	<1
Glcα1-4Glcα1-4	19O	<1	<1
Glcα1-4Glcα1-4Glcα1-4	19P	<1	<1
**LMW GAGs**			
Neocarratetraose-41, 3-di-O-sulphate (Na+)	12A	<1	<1

Neocarratetraose-41-O-sulphate (Na+)	12B	<1	<1
Neocarrahexaose-24,41, 3, 5-tetra-O-sulphate (Na+)	12C	<1	<1
Neocarrahexaose-41, 3, 5-tri-O-sulphate (Na+)	12D	<1	<1
Neocarraoctaose-41, 3, 5, 7-tetra-O-sulphate (Na+)	12E	<1	<1
Neocarradecaose-41, 3, 5, 7, 9-penta-O-sulphate (Na+)	12F	<1	<1
ΔUA-2S-GlcNS-6S	12G	<1	<1
ΔUA-GlucNS-6S	12H	<1	<1
ΔUA-2S-GlucNS	12I	<1	<1
ΔUA-2S-GlcNAc-6S	12J	<1	<1
ΔUA-GlcNAc-6S	12K	<1	<1
ΔUA-2S-GlcNAc	12L	<1	<1
ΔUA-GlcNAc	12M	<1	<1
ΔUA-GalNAc-4S (Delta Di-4S)	12N	<1	<1
ΔUA-GalNAc-6S (Delta Di-6S)	12O	<1	<1
ΔUA-GalNAc-4S,6S (Delta Di-disE)	12P	<1	<1
ΔUA-2S-GalNAc-4S (Delta Di-disB)	13A	<1	<1
ΔUA-2S-GalNAc-6S (Delta Di-disD)	13B	<1	<1
ΔUA-2S-GalNAc-4S-6S (Delta Di-tisS)	13C	<1	<1
ΔUA-2S-GalNAc-6S (Delta Di-UA2S)	13D	<1	<1
ΔUA-GlcNAc (Delta Di-HA)	13E	<1	<1
ΔUA→2S-GlcN-6S	14M	<1	<1
ΔUA→GlcN-6S	14N	<1	<1
ΔUA→2S-GlcN	14O	<1	<1
ΔUA→GlcN	14P	<1	<1
**HMW GAGs**			
(GlcAβ1-4GlcNAcβ1-3)$_8$-NH$_2$-ol	625	<1	<1
(GlcAβ1-3Glc*N*Acβ1-4)n (n=4)	13F	<1	<1
(GlcAβ1-3Glc*N*Acβ1-4)n (n=8)	13G	<1	<1
(GlcAβ1-3Glc*N*Acβ1-4)n (n=10)	13H	<1	<1
(GlcAβ1-3Glc*N*Acβ1-4)n (n=12)	13I	<1	<1
(GlcA/IdoAα/β1-4Glc*N*Acα1-4)n (n=200)	13J	<1	<1
(GlcA/IdoAβ1-3(±4/6S)Gal*N*Acβ1-4)n (n<250)	13K	<1	<1
((±2S)GlcA/IdoAα/b1-3(±4S)Gal*N*Acβ1-4)n (n<250)	13L	<1	<1
(GlcA/IdoAβ1-3(±6S)Gal*N*Acβ1-4)n (n<250)	13M	<1	<1
HA - 4 10mM	13N	<1	<1
HA - 6 10mM	130	<1	<1
HA - 8 9.7mM	13P	<1	<1
HA 10 7.83mM	14A	<1	<1
HA-12 6.5mM	14B	<1	<1
HA-14 5.6mM	14C	<1	<1
HA-16 4.9mM	14D	<1	<1
HA 30000 da 2.5mg/ml	14E	<1	<1
HA 107000 da 2.5mg/ml	14F	<1	<1
HA 190000 da 2.5 mg/ml	14G	<1	<1
HA 220000 da 2.5 mg/ml	14H	<1	<1
HA 1600000 da 2.5 mg/ml	14I	<1	<1
Heparin sulfate 5 mg/ml	14J	<1	<1
β1-3Glucan	14K	<1	<1
**Complex N-glycans**			
(Sia2-6A-GN-M)$_2$-3,6-M-GN-GNβ-sp4	627	<1	<1
Galβ1-4GlcNAcβ1-2Manα1-3(Galβ1-4GlcNAcβ1-2Manα1-6Man)β1-4GlcNAcβ1-4(Fucα1-6)GlcNAc	19A	<1	<1

Galβ1-4GlcNAcβ1-2(Galβ1-4GlcNAcβ1-4)Manα1-3(Galβ1-4GlcNAcβ1-2(Galβ1-4GlcNAcβ1-6)Manα1-6Man)β1-4GlcNAcβ1-4GlcNAc	19B	<1	<1
Neu5Acα2-6Galβ1-4GlcNAcβ1-2Manα1-3(Galβ1-4GlcNAcβ1-2Manα1-6)Manβ1-4GlcNAcβ1-4GlcNAc	19C	<1	<1
Neu5Acα2-6Galβ1-4GlcNAcβ1-2Manα1-3(Neu5Acα2-6Galβ1-4GlcNAcβ1-2Manα1-6)Manβ1-4GlcNAcβ1-4GlcNAc	19D	<1	<1
Galβ1-4GlcNAcβ1-2Manα1-3(Galβ1-4GlcNAcβ1-2Manα1-6)Manβ1-4GlcNAcβ1-4GlcNAc	19E	<1	<1
Neu5Acα2-6Galβ1-4GlcNAcβ1-2Manα1-3(Neu5Acα2-6Galβ1-4GlcNAcβ1-2Manα1-6)Manβ1-4GlcNAcβ1-4(Fucα1-6)GlcNAc	19F	<1	<1
Neu5Acα2-6Galβ1-4GlcNAcβ1-2(Neu5Acα2-6Galβ1-4GlcNAcβ1-4)Manα1-3(Neu5Acα2-6Galβ1-4GlcNAcβ1-2Manα1-6)Manβ1-4GlcNAcβ1-4GlcNAc	19G	<1	<1
GlcNAcβ1-2(GlcNAcβ1-4)Manα1-3(GlcNAcβ1-2Manα1-6)GlcNAcβ1-4Manβ1-4GlcNAcβ1-4GlcNAc	19H	<1	<1

Red indicates binding as shown in Fig 5. Binding indicates a value of greater than 1-fold of average background plus 3 standard deviations as described in the MIRAGE table (Table F).

Table F. Supplementary glycan microarray document based on MIRAGE guidelines DOI: 10.1093/glycob/cww118.

Classification	Guidelines
**1. Sample: Glycan Binding Sample**	
Description of Sample	<u>Sample names:</u><br>UclD^{LD} and UcaD^{LD}<br><br><u>Origin:</u> Produced as a recombinant protein in *E. coli*.<br><br><u>Method of preparation:</u><br>The preparation of UclD and UcaD are explained in the Materials and Methods section.
Sample modifications	UclD^{LD} and UcaD^{LD} are His-Tagged proteins.
Assay protocol	See *Materials and Methods*.
**2. Glycan Library**	
Glycan description for defined glycans	Glycans in this study are listed in Table E in S1 Text and as a published library in doi: 10.1371/journal.pntd.0004120.
Glycan description for undefined glycans	N/A.
Glycan modifications	Glycans were prepared in one of two ways for printing:<br>1. Glycans (with IDs in number/letter format; e.g. 1A, 4C, 7K) were sourced commercially from Dextra Laboratories, Elicityl and Carbosythn and were

	made into glycoamines using the protocol published in Day et al 2009 (doi: 10.1371/journal.pone.0004927).<br>2. Glycans (with IDs in number only format) were obtained from Prof Nicolai Bovin and were modified with spacers as per DOI: 10.1073/pnas.0407902101. The library of these glycans was first published in DOI: 10.1016/j.molimm.2009.06.010
**3. Printing Surface; e.g., Microarray Slide**	
Description of surface	Epoxy activated glass microarray slides.
Manufacturer	ArrayIt SuperEpoxy 3 (SME3).
Custom preparation of surface	N/A.
Non-covalent Immobilisation	N/A.
**4. Arrayer (Printer)**	
Description of Arrayer	SpotBot® Extreme Protein Microarray Spotter (ArrayIt, California, USA).
Dispensing mechanism	Contact printing using 946NS6 pins with a 6 pin in a 3 columns x 2 rows configuration.
Glycan deposition	Approximately 1.8 nl per spot is printed according to manufactures guidelines.<br>Glycan were at 500 µM in 50:50 DMF:DMSO.
Printing conditions	Array were printed with dehumidification at a maximum humidity of 60% relative humidity (Standard laboratory starting humidity of 75-90%) at 22ºC. Glycans were left to react with the slide for at least 8 hours after the print was completed.

5. Glycan Microarray with “Map”	
Array layout	The array consists of a single array of glycans split between 6 pins (3 columns x 2 rows) with 4500µm row and column spacing. Each pin printed a 20 columns x 16 rows with 200µm spot spacing (centre to centre) with a minimum spot size of 100µm. Each sample is printed in quadruplicate with each of the 6 print areas including at least three negative control samples (print solution only) and two positive control samples consisting of one sample of fluoroscienamine and one sample of a mixture of rabbit anti-mouse antibody labelled with Alexa 555 and Alexa 647. Positive controls provide proof of successful immobilization of the amine reagents and provides for orientation for analysis. The antibodies also can provide controls for secondary antibodies used in experiments (if applicable).
Glycan identification and quality control	Arrays are quality controlled by a range of measures. 1. Each printed array is post print scanned to confirm deposition of the glycans on the array surface prior to neutralization of the remaining slide surface. 2. Post neutralized slides are scanned again to monitor for remaining autofluorescence. 3. Slides are assayed with fluorescently labeled lectins: WGA-Texas Red (EY Laboratories) and ConA-FITC (EY Laboratories).
**6. Detector and Data Processing**	
Scanning hardware	ProScanArray 4 laser (488 nM, 532 nM, 595 nM, 647 nM) scanner (Perkin Elmer).
Scanner settings	Scanning resolution: 10µM<br>Laser channel: 532nM excitation / emission filter.<br>PMT: 70% gain<br>Scan powers: 100% laser power.
Image analysis software	ScanArray Express (Perkin Elmer).
Data processing	Data was exported as a CSV file and exported to Microsoft Excel.
**7. Glycan Microarray Data Presentation**	

Data presentation	Binding data is presented in Fig 5 together with SPR data. The yes/no binding including glycan identification is shown in Table E in S1 Text.
**8. Interpretation and Conclusion from Microarray Data**	
Data interpretation	We only use glycan arrays as a yes/no binding tool. Due to this we look only at binding that is unambiguously above background vs lack of binding above background. Average background + 3x standard deviation of the background of 20 sets of 4 spots of DMF:DMSO only spots is applied to determine if binding observed is significantly above background. Only spots with values equal to or greater than this value were considered as binding from data of any tested slide. These values are slide dependent.
Conclusions	UcaD^{LD} and UclD^{LD} both recognize glycans but even though they are highly similar the glycans recognized are different.

Supplementary Section References

1. Zhou Z, Alikhan NF, Mohamed K, Fan Y, Agama Study G, Achtman M. The EnteroBase user's guide, with case studies on Salmonella transmissions, Yersinia pestis phylogeny, and Escherichia core genomic diversity. Genome research. 2020;30(1):138-52. doi: 10.1101/gr.251678.119. PubMed PMID: 31809257.

2. Sievers F, Higgins DG. Clustal Omega for making accurate alignments of many protein sequences. Protein science : a publication of the Protein Society. 2018;27(1):135-45. doi: 10.1002/pro.3290. PubMed PMID: 28884485; PubMed Central PMCID: PMC5734385.

3. Kalyaanamoorthy S, Minh BQ, Wong TKF, von Haeseler A, Jermiin LS. ModelFinder: fast model selection for accurate phylogenetic estimates. Nature methods. 2017;14(6):587-9. doi: 10.1038/nmeth.4285. PubMed PMID: 28481363; PubMed Central PMCID: PMC5453245.

4. Zhang H, Gao S, Lercher MJ, Hu S, Chen WH. EvolView, an online tool for visualizing, annotating and managing phylogenetic trees. Nucleic acids research. 2012;40(Web Server issue):W569-72. doi: 10.1093/nar/gks576. PubMed PMID: 22695796; PubMed Central PMCID: PMC3394307.

5. Datsenko KA, Wanner BL. One-step inactivation of chromosomal genes in Escherichia coli K-12 using PCR products. Proceedings of the National Academy of Sciences of the United States of America. 2000;97(12):6640-5. Epub 2000/06/01. doi: 10.1073/pnas.120163297. PubMed PMID: 10829079; PubMed Central PMCID: PMC18686.

6. Totsika M, Beatson SA, Sarkar S, Phan MD, Petty NK, Bachmann N, et al. Insights into a multidrug resistant Escherichia coli pathogen of the globally disseminated ST131 lineage: genome analysis and virulence mechanisms. PLoS One. 2011;6(10):e26578. doi: 10.1371/journal.pone.0026578. PubMed PMID: 22053197; PubMed Central PMCID: PMCPMC3203889.

7. Nyerges A, Csorgo B, Nagy I, Balint B, Bihari P, Lazar V, et al. A highly precise and portable genome engineering method allows comparison of mutational effects across bacterial species. Proceedings of the National Academy of Sciences of the United States of America. 2016;113(9):2502-7. doi: 10.1073/pnas.1520040113. PubMed PMID: 26884157; PubMed Central PMCID: PMCPMC4780621.

8. Ulett GC, Webb RI, Schembri MA. Antigen-43-mediated autoaggregation impairs motility in Escherichia coli. Microbiology. 2006;152(Pt 7):2101-10. doi: 10.1099/mic.0.28607-0. PubMed PMID: 16804184.

9. Miller JH. A short course in bacterial genetics: a laboratory manual and handbook for Escherichia coli and related bacteria. Plainview, N.Y: Cold Spring Harbor Laboratory Press; 1992.

10. Rapid amplification of 5' complementary DNA ends (5' RACE). Nature methods. 2005;2(8):629-30. Epub 2005/09/09. PubMed PMID: 16145794.

11. Spaulding CN, Klein RD, Ruer S, Kau AL, Schreiber HL, Cusumano ZT, et al. Selective depletion of uropathogenic E. coli from the gut by a FimH antagonist. Nature. 2017;546(7659):528-32. Epub 2017/06/15. doi: 10.1038/nature22972. PubMed PMID: 28614296; PubMed Central PMCID: PMCPMC5654549.

12. Russell CW, Fleming BA, Jost CA, Tran A, Stenquist AT, Wambaugh MA, et al. Context-Dependent Requirements for FimH and Other Canonical Virulence Factors in Gut Colonization by Extraintestinal Pathogenic Escherichia coli. Infection and immunity. 2018;86(3). doi: 10.1128/IAI.00746-17. PubMed PMID: 29311232; PubMed Central PMCID: PMC5820936.

13. Kelley LA, Sternberg MJ. Protein structure prediction on the Web: a case study using the Phyre server. Nature Protocols. 2009;4(3):363-71. doi: 10.1038/nprot.2009.2. PubMed PMID: 19247286.

14. Furlong EJ, Lo AW, Kurth F, Premkumar L, Totsika M, Achard MES, et al. A shape-shifting redox foldase contributes to Proteus mirabilis copper resistance. Nature communications. 2017;8:16065. doi: 10.1038/ncomms16065. PubMed PMID: 28722010; PubMed Central PMCID: PMC5524982.

15. Shewell LK, Day CJ, Jen FE, Haselhorst T, Atack JM, Reijneveld JF, et al. All major cholesterol-dependent cytolysins use glycans as cellular receptors. Science advances. 2020;6(21):eaaz4926. doi: 10.1126/sciadv.aaz4926. PubMed PMID: 32494740; PubMed Central PMCID: PMC7244308.

16. Waespy M, Gbem TT, Elenschneider L, Jeck AP, Day CJ, Hartley-Tassell L, et al. Carbohydrate Recognition Specificity of Trans-sialidase Lectin Domain from Trypanosoma congolense. PLoS neglected tropical diseases. 2015;9(10):e0004120. doi: 10.1371/journal.pntd.0004120. PubMed PMID: 26474304; PubMed Central PMCID: PMC4608562.

17. McPhillips TM, McPhillips SE, Chiu HJ, Cohen AE, Deacon AM, Ellis PJ, et al. Blu-Ice and the distributed control system: software for data acquisition and instrument control at macromolecular crystallography beamlines. J Synchrotron Radiat. 2002;9(Pt 6):401-6. Epub 2002/11/01. PubMed PMID: 12409628.

18. Winn MD, Ballard CC, Cowtan KD, Dodson EJ, Emsley P, Evans PR, et al. Overview of the CCP4 suite and current developments. Acta Crystallographica Section D. 2011;67(Pt 4):235-42. Epub 2011/04/05. doi: 10.1107/S0907444910045749. PubMed PMID: 21460441; PubMed Central PMCID: PMC3069738.

19. Pannu NS, Waterreus WJ, Skubak P, Sikharulidze I, Abrahams JP, de Graaff RA. Recent advances in the CRANK software suite for experimental phasing. Acta Crystallographica Section D. 2011;67(Pt 4):331-7. doi: 10.1107/S0907444910052224. PubMed PMID: 21460451; PubMed Central PMCID: PMCPMC3069748.

20. Sheldrick GM. A short history of SHELX. Acta Crystallographica Section A. 2008;64(Pt 1):112-22. doi: 10.1107/S0108767307043930. PubMed PMID: 18156677.

21. Cowtan K. The Buccaneer software for automated model building. 1. Tracing protein chains. Acta Crystallographica Section D. 2006;62(Pt 9):1002-11. doi: 10.1107/S0907444906022116. PubMed PMID: 16929101.

22. Murshudov GN, Skubak P, Lebedev AA, Pannu NS, Steiner RA, Nicholls RA, et al. REFMAC5 for the refinement of macromolecular crystal structures. Acta Crystallographica Section D. 2011;67(Pt 4):355-67. doi: 10.1107/S0907444911001314. PubMed PMID: 21460454; PubMed Central PMCID: PMCPMC3069751.

23. McCoy AJ. Solving structures of protein complexes by molecular replacement with Phaser. Acta Crystallographica Section D. 2007;63(Pt 1):32-41. Epub 2006/12/14. doi: 10.1107/S0907444906045975. PubMed PMID: 17164524; PubMed Central PMCID: PMCPMC2483468.

24. Adams PD, Baker D, Brunger AT, Das R, DiMaio F, Read RJ, et al. Advances, interactions, and future developments in the CNS, Phenix, and Rosetta structural biology software systems. Annual review of biophysics. 2013;42:265-87. doi: 10.1146/annurev-biophys-083012-130253. PubMed PMID: 23451892.

25. Emsley P, Lohkamp B, Scott WG, Cowtan K. Features and development of Coot. Acta Crystallographica Section D. 2010;66(Pt 4):486-501. PubMed PMID: 20383002; PubMed Central PMCID: PMC2852313.

26. Chen VB, Arendall WB, 3rd, Headd JJ, Keedy DA, Immormino RM, Kapral GJ, et al. MolProbity: all-atom structure validation for macromolecular crystallography. Acta Crystallographica Section D. 2010;66(Pt 1):12-21. Epub 2010/01/09. doi: 10.1107/S0907444909042073. PubMed PMID: 20057044; PubMed Central PMCID: PMC2803126.

27. Kabsch W. XDS. Acta Crystallographica Section D. 2010;66(Pt 2):125-32. Epub 2010/02/04. doi: 10.1107/S0907444909047337. PubMed PMID: 20124692; PubMed Central PMCID: PMC2815665.

28. Trott O, Olson AJ. AutoDock Vina: improving the speed and accuracy of docking with a new scoring function, efficient optimization, and multithreading. Journal of computational chemistry. 2010;31(2):455-61. doi: 10.1002/jcc.21334. PubMed PMID: 19499576; PubMed Central PMCID: PMC3041641.

29. Krieger E, Koraimann G, Vriend G. Increasing the precision of comparative models with YASARA NOVA--a self-parameterizing force field. Proteins. 2002;47(3):393-402. PubMed PMID: 11948792.

30. Kjaergaard K, Schembri MA, Ramos C, Molin S, Klemm P. Antigen 43 facilitates formation of multispecies biofilms. Environ Microbiol. 2000;2(6):695-702. doi: 10.1046/j.1462-2920.2000.00152.x. PubMed PMID: 11214802.

31. Rasko DA, Rosovitz MJ, Myers GS, Mongodin EF, Fricke WF, Gajer P, et al. The pangenome structure of Escherichia coli: comparative genomic analysis of E. coli commensal and pathogenic isolates. J Bacteriol. 2008;190(20):6881-93. Epub 20080801. doi: 10.1128/JB.00619-08. PubMed PMID: 18676672; PubMed Central PMCID: PMCPMC2566221.

32. Stapleton A, Moseley S, Stamm WE. Urovirulence determinants in Escherichia coli isolates causing first-episode and recurrent cystitis in women. J Infect Dis. 1991;163(4):773-9. doi: 10.1093/infdis/163.4.773. PubMed PMID: 1672702.

33. Chen SL, Hung CS, Xu J, Reigstad CS, Magrini V, Sabo A, et al. Identification of genes subject to positive selection in uropathogenic strains of Escherichia coli: a comparative genomics

approach. Proc Natl Acad Sci U S A. 2006;103(15):5977-82. Epub 20060403. doi: 10.1073/pnas.0600938103. PubMed PMID: 16585510; PubMed Central PMCID: PMCPMC1424661.

34. Mulvey MA, Schilling JD, Hultgren SJ. Establishment of a persistent Escherichia coli reservoir during the acute phase of a bladder infection. Infect Immun. 2001;69(7):4572-9. doi: 10.1128/IAI.69.7.4572-4579.2001. PubMed PMID: 11402001; PubMed Central PMCID: PMCPMC98534.

35. Petty NK, Ben Zakour NL, Stanton-Cook M, Skippington E, Totsika M, Forde BM, et al. Global dissemination of a multidrug resistant Escherichia coli clone. Proc Natl Acad Sci USA. 2014;111(15):5694-9. doi: 10.1073/pnas.1322678111. PubMed PMID: 24706808; PubMed Central PMCID: PMC3992628.

36. Furlong EJ, Lo AW, Kurth F, Premkumar L, Totsika M, Achard MES, et al. A shape-shifting redox foldase contributes to Proteus mirabilis copper resistance. Nat Commun. 2017;8:16065. Epub 2017/07/20. doi: 10.1038/ncomms16065. PubMed PMID: 28722010; PubMed Central PMCID: PMCPMC5524982.

37. Datsenko KA, Wanner BL. One-step inactivation of chromosomal genes in Escherichia coli K-12 using PCR products. Proc Natl Acad Sci U S A. 2000;97(12):6640-5. doi: 10.1073/pnas.120163297. PubMed PMID: 10829079; PubMed Central PMCID: PMCPMC18686.

38. Martinez E, Bartolome B, de la Cruz F. pACYC184-derived cloning vectors containing the multiple cloning site and lacZ alpha reporter gene of pUC8/9 and pUC18/19 plasmids. Gene. 1988;68(1):159-62. doi: 10.1016/0378-1119(88)90608-7. PubMed PMID: 2851489.

39. Farinha MA, Kropinski AM. Construction of broad-host-range plasmid vectors for easy visible selection and analysis of promoters. J Bacteriol. 1990;172(6):3496-9. doi: 10.1128/jb.172.6.3496-3499.1990. PubMed PMID: 2111810; PubMed Central PMCID: PMCPMC209165.

40. Guzman LM, Belin D, Carson MJ, Beckwith J. Tight regulation, modulation, and high-level expression by vectors containing the arabinose PBAD promoter. J Bacteriol. 1995;177(14):4121-30. doi: 10.1128/jb.177.14.4121-4130.1995. PubMed PMID: 7608087; PubMed Central PMCID: PMCPMC177145.

41. Schembri MA, Hjerrild L, Gjermansen M, Klemm P. Differential expression of the Escherichia coli autoaggregation factor antigen 43. J Bacteriol. 2003;185(7):2236-42. Epub 2003/03/20. doi: 10.1128/jb.185.7.2236-2242.2003. PubMed PMID: 12644494; PubMed Central PMCID: PMCPMC151503.

42. Evans PR, Murshudov GN. How good are my data and what is the resolution? Acta Crystallogr D Biol Crystallogr. 2013;69(Pt 7):1204-14. doi: 10.1107/S0907444913000061. PubMed PMID: 23793146; PubMed Central PMCID: PMCPMC3689523.
